# Gradual Recovery of Building Plumbing-Associated Microbial Communities after Extended Periods of Altered Water Demand during the COVID-19 Pandemic

**DOI:** 10.1021/acs.est.2c07333

**Published:** 2023-02-16

**Authors:** Solize Vosloo, Linxuan Huo, Umang Chauhan, Irmarie Cotto, Benjamin Gincley, Katherine J. Vilardi, Bryan Yoon, Kaiqin Bian, Marco Gabrielli, Kelsey J. Pieper, Aron Stubbins, Ameet J. Pinto

**Affiliations:** † Department of Civil and Environmental Engineering, Northeastern University, 360 Huntington Avenue, Boston, Massachusetts 021115, United States; ‡ School of Civil and Environmental Engineering, Georgia Institute of Technology, 311 Ferst Drive, Atlanta, Georgia 30318, United States; § Dipartimento di Ingegneria Civile e Ambientale - Sezione Ambientale, Politecnico di Milano, 20133 Milan, Italy

**Keywords:** premise plumbing, stagnation, water quality, COVID-19 pandemic, flow cytometry

## Abstract

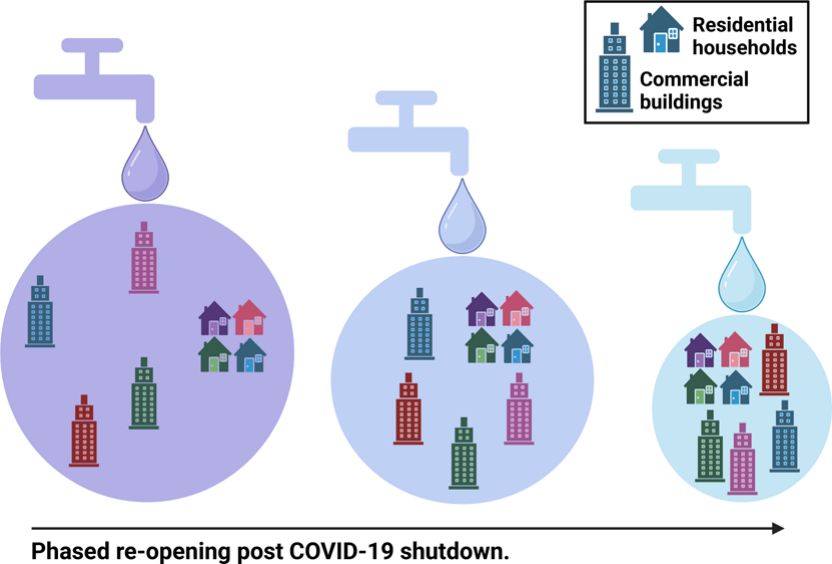

COVID-19 pandemic-related building restrictions heightened drinking water microbiological safety concerns post-reopening due to the unprecedented nature of commercial building closures. Starting with phased reopening (i.e., June 2020), we sampled drinking water for 6 months from three commercial buildings with reduced water usage and four occupied residential households. Samples were analyzed using flow cytometry and full-length 16S rRNA gene sequencing along with comprehensive water chemistry characterization. Prolonged building closures resulted in 10-fold higher microbial cell counts in the commercial buildings [(2.95 ± 3.67) × 10^5^ cells mL^–1^] than in residential households [(1.11 ± 0.58) × 10^4^ cells mL^–1^] with majority intact cells. While flushing reduced cell counts and increased disinfection residuals, microbial communities in commercial buildings remained distinct from those in residential households on the basis of flow cytometric fingerprinting [Bray–Curtis dissimilarity (*d*_BC_) = 0.33 ± 0.07] and 16S rRNA gene sequencing (*d*_BC_ = 0.72 ± 0.20). An increase in water demand post-reopening resulted in gradual convergence in microbial communities in water samples collected from commercial buildings and residential households. Overall, we find that the gradual recovery of water demand played a key role in the recovery of building plumbing-associated microbial communities as compared to short-term flushing after extended periods of reduced water demand.

## Abstract 2

Water demand recovery alleviated the impacts of extended stagnation due to COVID-19 response measures on building plumbing-associated microbial communities.

## Introduction

Social distancing policies enacted in March 2020 during the initial wave of the coronavirus (COVID-19) pandemic to limit disease spread^1,2^ caused widespread building closures in the nonresidential sector (i.e., commercial, industrial, etc.). This limited building occupancy and altered individual and societal behaviors (e.g., hygiene practices),^3^ which significantly impacted water demand.^4−10^ The impact of policy interventions related to the COVID-19 pandemic on water demand has recently been addressed.^4−10^ Several studies have reported significant reductions in water demand across buildings in nonresidential sectors.^5,8−10^ For example, Li et al. reported a decrease of 11.6% in water demand across buildings in California (United States), while Kalbusch et al. documented reductions in water demand, ranging between 30% and 53%, across buildings in Joinville, Brazil. In contrast, reports on residential sectors indicate increases in water demand,^6−9^ as well as anomalies in the timing and magnitude of water demand peaks.^7,10^

Reduced water demand and the resultant increase in stagnation times in building plumbing systems have been linked to water quality deterioration,^11^ microbial regrowth,^11−14^ and public health concerns, including opportunistic pathogen growth,^13,15−17^ metal leaching,^18^ and disinfectant byproduct formation.^19^ Unlike drinking water distribution systems (DWDSs), building plumbing systems can experience a significant loss of disinfectant residual, have large temperature gradients, and consist of unique site-specific features (e.g., variable materials, high surface area:volume ratios, etc.) that can promote microbial and opportunistic pathogen growth during periods of low water demand.^11,17,20^ In contrast to DWDSs that are subject to water monitoring plans, building plumbing systems rely on guidance documents to ensure safe water quality at point of use.^21^ Remedial actions in guidance documents to minimize water quality risks in buildings impacted by COVID-19 social distancing policies include continuous or shock disinfection, routine flushing, and diagnostic testing of opportunistic pathogens (e.g., *Legionella* spp. and nontuberculous *Mycobacterium* spp.).^21,22^ The scope and details of these remedial actions vary widely and depend on site-specific factors, as well as the risk(s) that needs to be mitigated.

Flushing replenishes disinfectant residual concentrations and reduces microbial loads in building plumbing systems.^11,12,16,17^ The effectiveness of flushing practices is typically assessed by performing intermittent testing of temperature and disinfectant residual concentrations,^21^ with stabilization of these measures being indicative of baseline water quality conditions;^14^ however, neither of these measures provides direct microbiological insight. Flow cytometry can provide rapid quantitative measures of cell concentrations as well as shifts in microbial community fingerprints.^23−26^ For example, it has been used to monitor pipe flushing after overnight stagnation in buildings^12,14^ and during maintenance operations in the DWDSs.^27^ Managing water in buildings is crucial to avoid waterborne microbial health risks; however, consensus in guidance documents and expert opinions on design and operational topics that are critical for water quality management are lacking.^21,22^ Data generated from field studies can drive the development of evidence-based best practices for water quality management, which can facilitate more informed and coherent decisions.

This study examines changes in drinking water microbial communities in response to altered water demand during the COVID-19 pandemic in Boston, Massachusetts. Drinking water samples from commercial building and residential household taps were collected monthly over a period of 6 months starting with the first week of the building reopening in June 2020 and analyzed for a suite of chemical and microbiological parameters. The study objectives were (1) to evaluate the impact of extended periods of altered water demand on drinking water microbial communities, (2) to assess the impact of flushing on them, and (3) to determine the impact of recovering water demand with building reopening on microbial communities in commercial buildings compared to residential households. With these three objectives our overall goal was to assess factors influencing the recovery of building-plumbing associated microbial communities post-reopening and time-scale of the recovery itself. Since our study does not include samples from pre-COVID-19-related shutdowns, we define recovery at commercial locations as (1) the convergence of their microbial communities with those at residential locations indicating overall attenuation of shutdown-related stagnation effects, (2) increased similarity in microbial communities between commercial locations indicating alleviation of stagnation-induced effects that may be unique to each location, and (3) reduction in the rate of change in microbial community structure and membership during short-term flushing indicating reduced impact of stagnation within the building plumbing.

## Materials and Methods

### Description of the Study Site and Sample Collection

College campuses across Massachusetts underwent the transition to remote learning in March 2020. As a result, classrooms, residence halls, and assembly buildings (e.g., dining services) were closed and non-essential research operations were halted on multiple campuses, including Northeastern University (NEU) in Boston. On June 1, NEU partially reopened for research activities at established laboratory capacity limits of 25% for the remainder of the year. For the fall 2020 semester, residence halls and classrooms opened at reduced capacity and NEU operated with hybrid learning, which included part in-person and part remote learning. In this study, three NEU buildings (hereafter, commercial buildings) and four residential households were included. These site types (commercial buildings and residential households) (i) had ranges of size, age, and functionality, (ii) were situated within 5 miles of each other, and (iii) were served with chloraminated water from the same DWDS. Cold water taps at each site type, i.e., commercial building (three sites, two taps per site) and residential household (four sites, one tap per site), were sampled during the first week of each month for 6 months starting the month of building reopening (i.e., June 2020). The sampled taps were in residential kitchens (*n* = 2), commercial kitchenettes (*n* = 3), residential bathrooms (*n* = 2), and research laboratories in commercial buildings (*n* = 3). Flush profiles were conducted after overnight stagnation at all 10 taps between 6:00 and 10:00 a.m. on two consecutive days (Table S1). Seven 10 mL samples were collected from each tap in sterile 15 mL polyethylene centrifuge tubes (Falcon, catalog no. 352196) for flow cytometric analyses; this included the first draw sample following overnight stagnation [time point 1 (TP_0_, 0 min)] and six samples collected at 5 min intervals over a flush period of 30 min (TP_5_, 5 min; TP_10_, 10 min; TP_15_, 15 min; TP_20_, 20 min; TP_25_, 25 min; and TP_30_, 30 min). An additional 2 L sample was collected at TP_0_ and TP_30_ in sterile narrow-mouth polycarbonate bottles (Thermo Scientific, catalog no. DS22050210) and used for DNA-based microbial community characterization, as well as 500 mL samples in wide-mouth HDPE bottles (Thermo Scientific, catalog no. 02-896-2E) for chemical analysis. Temperature measurements were obtained at 10 s intervals using an Elitech GSP-6 data logger during the flushing period and flow rates averaged at 4.10 ± 1.80 L min^–1^ (Table S1).

### Water Chemistry Analysis

Temperature, pH, conductivity, and dissolved oxygen were measured using the Orion Star A325 pH/Conductivity Portable Multiparameter Meter (Thermo Scientific, catalog no. STARA3250). Total chlorine was measured using HACH Method 8167 (HACH, catalog no. 2105669). Ammonium, nitrate, and nitrite were measured using the Nitrogen-Ammonia Reagent Set (method 10023, HACH, catalog no. 2604545), NitraVer X Nitrogen-Nitrate Reagent Set (method 10020, HACH, catalog no. 2605345), and NitriVer 3 TNT Reagent Set (method 10019, catalog no. 2608345), respectively. All HACH measurements were performed on the DR1900 Portable Spectrophotometer (HACH, catalog no. DR190001H). For metal analysis, samples were acidified with 2% nitric acid and digested for a minimum of 16 h before being analyzed via inductively coupled plasma mass spectrometry (ICP-MS) according to method 3125 B.^28^ Blanks and spikes of known concentrations were measured every 10 samples for quality control. Samples for dissolved organic carbon (DOC) and total dissolved nitrogen (TDN) were acidified to pH <2 using HCl before being analyzed using a Shimadzu TOC-V_CPH_ total organic carbon analyzer with a total nitrogen module attached.^29^ Certified DOC and TDN quality references from ULTRA Scientific (Agilent, catalog nos. QCI-731 and QCI-745) were measured to confirm precision and accuracy.

### Flow Cytometry Analysis

Samples were processed in triplicate to obtain flow cytometric (FCM) measurements of total cell concentrations (TCC) and intact cell concentrations (ICC) as described previously^30−32^ using SYBR Green I (SG) (Invitrogen, catalog no. S7585) at 10 μL mL^–1^ (TCC measurements) or SG combined with propidium iodide (PI) (Molecular Probes, catalog no. P3566) (3 μM final concentration) at 12 μL mL^–1^ (ICC measurements). Five negative controls were processed in parallel with the samples, including (i) unstained UltraPure DNase/RNase-Free Distilled Water (Thermo Fisher Scientific, catalog no. 10977015), (ii) SG-stained UltraPure DNase/RNase-Free Distilled Water, (iii) SGPI-stained UltraPure DNase/RNase-Free Distilled Water, (iv) a SG-stained 0.22 μm filtered drinking water sample, and (v) a SGPI-stained 0.22 μm filtered drinking water sample. FCM analysis was performed on 50 μL samples at a flow rate of 66 μL min^–1^ using a BD Accuri C6 flow cytometer (BD Accuri cytometers) equipped with a 50 mW solid-state laser emitting light at a fixed wavelength of 488 nm. Green and red fluorescence intensities were collected at FL1 = 533 ± 30 nm and FL3 > 670 nm, respectively, along with sideward (SSC) and forward (FCS) scatter light intensities. Flow cytometry standard (FCS) files were processed in R version 4.1.2^33^ using Phenoflow version 1.1^23^ and its dependencies (https://github.com/CMET-UGent/Phenoflow_package). Briefly, FCS files were imported into R using flowCore version 2.2.0,^34^ and then four parameters in signal height format (i.e., FL1-H, FL3-H, SSC-H, and FSC-H) were extracted and rescaled using hyperbolic arcsine transformations. A fixed polygonal gate was applied on the FL1-H and FL3-H graph to separate the signal (i.e., cells) and background noise [i.e., instrument noise and (in)organic background]^35,36^ and to estimate measures of TCC and ICC as the number of cells per microliter. The function flowBasis was used for advance fingerprinting using TCC data, with *n*_bin_ set to 128 and the bandwidth set to 0.01.^23^ From this, biodiversity estimates, i.e., phenotypic diversity index (*D*_2_) and evenness, were calculated as well as measures of β diversity through Bray–Curtis dissimilarity using vegan version 2.6-2.^37^ All biodiversity measures were performed after resampling of the sample with the smallest cell count.

### DNA Extraction and Full-Length 16S rRNA Gene Sequencing on the PacBio Platform

The microbial biomass was concentrated from a 1500 mL sample by filtration through a 0.22 μm poly(ether sulfone) membrane-enclosed filter unit (EMD Millipore, catalog no. SVGP01050) using the Geopump Peristaltic Pump and sterile silicone tubing (Geotech Environmental Equipment, Inc., catalog nos. 91350113 and 77050000, respectively). Extraction of DNA from the Sterivex filter membrane was performed as outlined previously.^38^ Three negative controls [control 1 (CON_1_), reagent blank; control 2 (CON_2_), unused poly(ether sulfone) membrane filter; and control 3 (CON_3_), poly(ether sulfone) membrane filter treated with autoclaved distilled water] were processed in parallel with the samples. DNA was quantified on the Qubit 4 Fluorometer (Thermo Fisher Scientific, catalog no. Q33238) using the Qubit dsDNA High Sensitivity Assay Kit (Thermo Fisher Scientific, catalog no. Q32851). All DNA extracts were stored at −80 °C until further analysis.

DNA extracts were sent for full-length 16S rRNA gene sequencing at the Georgia Genomics and Bioinformatics Core (University of Georgia, Athens, GA) on the PacBio Sequel II platform. Full-length 16S rRNA genes were amplified from the DNA extracts using the KAPA HiFi Hot Start ReadyMix PCR Kit (KAPA Biosystems) and a universal primer set (27F, AGRGTTYGATYMTGGCTCAG; and 1492R, RGYTACCTTGTTACGACTT) tailed with sample-specific barcode sequences to allow for multiplex sequencing. Polymerase chain reaction (PCR) conditions included an initial denaturation of 95 °C for 3 min and then 20 cycles at 95 °C for 30 s, 57 °C for 30 s, and 72 °C for 60 s. PCR products were pooled in equimolar concentrations across samples, and SMRTbell libraries were prepared using the SMRTbell Express Template Prep Kit 2.0 according to the manufacturer’s instructions. Purified SMRTbell libraries were sequenced on a single PacBio Sequel cell. Raw reads were demultiplexed, followed by the generation of circular consensus sequence (CCS) reads using applications of the SMRT Link software package. Processing of CCS reads in FASTQ format was performed using DADA2 version 1.14 R and its workflow as outlined previously.^39^ Subsequent analysis was performed using phyloseq version 3.14 R.^40^ Specifically, the ASV table, taxonomy table, and sample metadata were combined into a single object using phyloseq{phyloseq}. The phyloseq object was used as input in rarefy_even_depth{phyloseq} for subsampling without replacement. The rarefied phyloseq object was used to obtain α diversity measures (i.e., observed species, Shannon diversity index, and Pielou’s evenness) using the function estimate_richness{phyloseq}. For β diversity measures, the ASV table was extracted from the rarefied phyloseq object and used as input in vegdist{vegan} to determine Bray–Curtis dissimilarity distances.

### Phylogenetic Analysis

Full-length 16S rRNA gene sequences of ASVs belonging to genera *Nitrosomonas*, *Nitrospira*, *Mycobacterium*, *Legionella*, and *Pseudomonas* were extracted and independently aligned against publicly available ref 16S rRNA gene sequences downloaded from NCBI (Table S2) using MUSCLE version 5.1.^41^ The resulting alignments in FASTA format were trimmed using trimAL version 1.4^42^ and converted to PHYLIP format for phylogenetic inference using maximum likelihood in IQ-TREE version 2.0.3.^43^ All maximum likelihood phylogenetic trees were reconstructed in iTOL version 6.3.1.^44^

### Statistical Analysis

Statistical analysis was performed using packages in R version 4.1.2.^33^ Water chemistry measures and flow cytometric measurements were assessed for normality using the Shapiro–Wilks test provided in stats version 3.6.3,^33^ and then comparisons between groups were performed with either an independent *t* test (t.test{stats}; parametric test for independent samples) or a Mann–Whitney U test (wilcox.test{stats}; nonparametric test for independent samples). NMDS was performed using metaMDS provided in vegan version 2.6-2^37^ with Bray–Curtis dissimilarity distances, and permutational multivariate analysis of variance (PERMANOVA) was performed using adonis{vegan}. To identify factors associated with a change in the microbial community composition, water chemistry data were z-score standardized and considered for Bray–Curtis distance-based redundancy analysis (dbRDA) using dbrda{vegan}. To address bias associated with collinearity, colinear variables exhibiting significant Pearson correlation coefficients of more than 0.70 and less than −0.70 were identified and then placed into clusters, followed by the selection of one variable per cluster for dbRDA.^45^ Excluded colinear variables were considered for discussion and placed in brackets, with “POS” denoting positive correlations [Pearson correlation coefficient (*R*^2^) > 0.70] and “NEG” denoting negative correlations (*R*^2^ < −0.70). The significance of each response variable was tested with an ANOVA (anova{stats}), and only response variables with significance were kept in the model. The explanatory value of significant response variables was determined with variation partitioning analysis using varpart{vegan}. All plots were generated using ggplot2 version 3.3.6.^46^

### Data Availability

The raw FCS files have been deposited in FlowRepository and are publicly available under accession number FR-FCM-Z4SR, and 16S rRNA gene sequences are available on NCBI at BioProject accession number PRJNA885853.

## Results and Discussion

### Extended Periods of Altered Water Demand Significantly Impacted Water Quality Metrics in Commercial Buildings Compared to Residential Households

**Figure 1:**
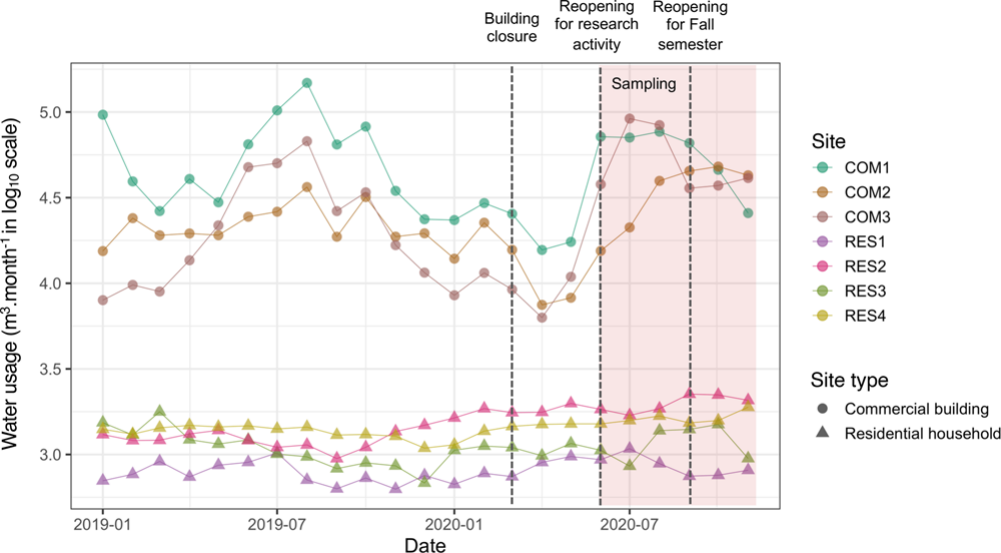
Water demand (cubic meters per month) at three commercial building (COM, ●) and four residential household (RES, ▲) sites from January 2019 to November 2020. The dashed lines indicate the closure of commercial building sites due to COVID-19 policy interventions, commercial building sites reopening for research activity, and commercial building sites reopening for the fall 2020 academic semester. The red shaded area indicates the 6 month sampling period (June–November 2020).

Extended stagnation in building plumbing systems due to low water demand can impact water quality.^22,47^ Under normal building operations, stagnation-induced effects over multiple time scales (i.e., overnight and over weekends) have been linked to water quality deterioration resulting in microbial regrowth,^11−14^ opportunistic pathogen growth,^13,15−17^ and metal (e.g., lead) leaching.^18^ In this study, the building plumbing systems of the commercial building sites had reduced water demand for approximately 14 weeks (Figure 1). Monthly water demand levels at the commercial and residential locations from January to February 2020 were similar to those in 2019 (Figure 1 and Table S3); however, the water demand at commercial buildings between March and May 2020 (12928 ± 5766 m^3^ month^–1^) was 39–46% lower than in 2019 (22088 ± 9076 m^3^ month^–1^). These observations concur with recent studies that reported reductions in water demand across commercial, industrial, and institutional buildings due to COVID-19 social distancing policies.^5,8−10^ In contrast, there was a modest (6%) increase in water demand at residential household sites between March and May 2020 [1320 ± 398 m^3^ month^–1^ (Table S3)] compared to 2019 [1245 ± 277 m^3^ month^–1^ (Table S3)], which also agrees with recent studies^1−3,6−9,11−18,22,47^ The TCC measures in commercial building [(2.95 ± 3.67) × 10^5^ cells mL^–1^] within the first week of building reopening were significantly higher than in residential household sites [(1.11 ± 0.58) × 10^4^ cells mL^–1^; with a Mann–Whitney U test, *p* = 3 × 10^–16^ (Table S4A)]. In addition, TCC measures for the commercial building first draw samples (TP_0_) [(8.07 ± 3.91) × 10^5^ cells mL^–1^] were approximately 8-fold higher than that of the final samples (TP_30_) [(1.00 ± 0.95) × 10^5^ cells mL^–1^; with a Mann–Whitney U test, *p* = 0.002 (Table S4B)], while smaller values (i.e., 2-fold differences) were observed between the TP_0_ and TP_30_ samples of the residential household sites [(2.07 ± 1.20) × 10^4^ and (1.18 ± 0.12) × 10^4^ cells mL^–1^, respectively (Table S4B)]. High TCC measures of the commercial building samples were associated with higher temperatures (22.42 ± 3.32 °C), lower total chlorine concentrations (0.51 ± 0.68 mg L^–1^), and increased levels of metals, particularly copper (123.64 ± 91.52 μg L^–1^), iron (24.01 ± 38.32 μg L^–1^), and manganese (6.09 ± 2.57 μg L^–1^) when compared to those of the residential household sites [via a Mann–Whitney U test, all *p* = 1.6 × 10^–5^ to 0.048 (Table S4A)]. High microbial cell concentrations along with low residual disinfectant residuals and increased temperatures concur with those of previous studies that were conducted over shorter stagnation time scales (i.e., overnight and over weekends)^11,12,14,48^ and confirm that reduced water demand and extended stagnation impacted the water quality in large commercial buildings subject to building closures during the COVID-19 pandemic. The increase in total cell concentrations in commercial buildings with reduced occupancy exceeded concentrations reported in DWDS and buildings subjected to shorter stagnation time scales by at least 10-fold. Although an increase in cell counts does not necessarily represent a health hazard, undesired microbial growth can alter water taste, odor, and color, which are leading causes of water quality complaints.

**Figure 2:**
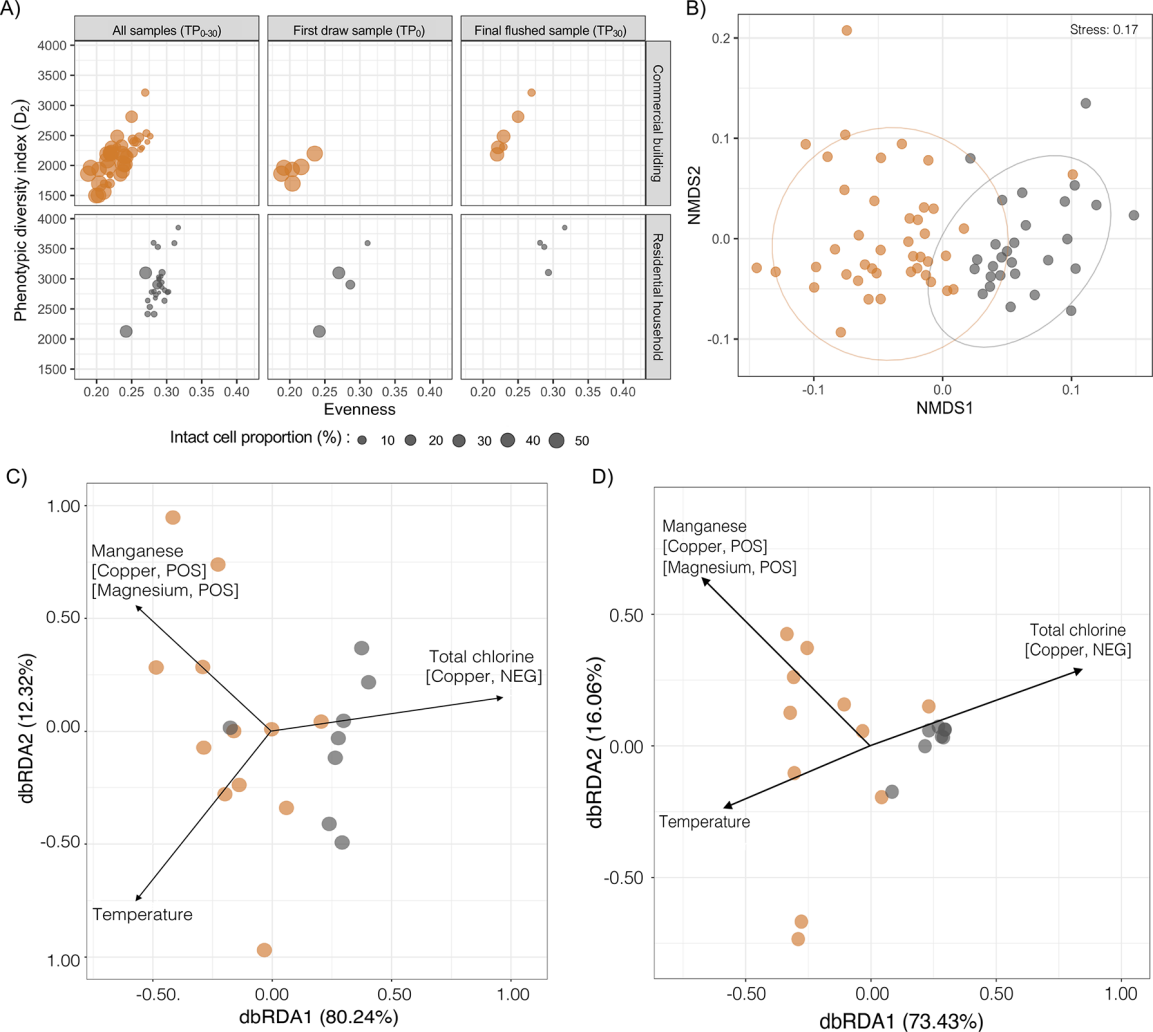
(A) Bubble plot showing the proportion of intact cells (depicted by size) with phenotypic diversity and evenness indices of TP_0–30_ samples that were collected during the first week of building reopening from the commercial building and residential household sites. Panels from the left to right represent measures related to (i) all samples (TP_0–30_), (ii) first draw samples (TP_0_), and (iii) final samples (TP_30_). (B) NMDS plot illustrating differences in microbial community composition among TP_0–30_ samples of commercial building and residential household sites estimated using Bray–Curtis distances derived from flow cytometric fingerprinting data. Ellipses drawn at a 95% confidence limit. (C and D) Bray–Curtis dissimilarity-based dbRDA biplot using flow cytometric and 16S rRNA gene sequencing data illustrating the relationship among the site type, water chemistry parameters, and microbial community structure of TP_0_ and TP_30_ samples of the commercial building (orange) and residential household (gray) sites. Water quality parameters that significantly explained differences in community composition based on PERMANOVA analysis are shown as black arrows. Colinear variables are shown in brackets, with “POS” and “NEG” denoting positive and negative correlations at Pearson’s correlation coefficients of >0.70 and <0.70, respectively.

Microbial communities in water samples collected from commercial building sites had greater proportions of intact cells, averaging 29.81 ± 20.29%, and were less diverse (*D*_2_ = 2126 ± 350) and even (evenness = 0.23 ± 0.02) compared to those in residential household samples (Table S4A and Figure 2A). Differences in intact cell proportions between the commercial building TP_0_ and TP_30_ samples were ∼2-fold (52.98 ± 3.42% and 24.6 ± 14.22% for TP_0_ and TP_30_, respectively), and the TP_30_ microbial communities were more diverse and even (*D*_2_ = 2551 ± 391, and evenness = 0.24 ± 0.02) than those of the TP_0_ samples (*D*_2_ = 1938 ± 163, and evenness = 0.21 ± 0.02) (Figure 2A). In contrast, microbial communities in TP_0_ and TP_30_ samples from residential household sites were not significantly different except for higher intact cell proportions in TP_0_ than in TP_30_ samples [17.06 ± 12.68% and 2.00 ± 1.19% for TP_0_ and TP_30_, respectively (Figure 2A and Table S4B)]. Higher intact cell proportions associated with the first draw TP_0_ samples of both site types likely relate to microbial growth because of stagnant conditions.^11−14^ α diversity indices derived from 16S rRNA gene sequencing data presented similar findings between site types (Table S4A) and between the TP_0_ and TP_30_ samples of each site type (Table S4B). The lower diversity of TP_0_ samples compared to that of TP_30_ samples (Table S4B) suggests proliferation of select taxa under stagnant water conditions.

Bray–Curtis dissimilarity derived from flow cytometric fingerprinting showed clear clustering of the samples on the basis of site type, which explained approximately 22% of the variation in the microbial community structure based on PERMANOVA [*F*(1, 68) = 18.65, *R*^2^ = 0.215, and *p* = 0.001 (Figure 2B)]. This distinct clustering was also clear with 16S rRNA gene sequencing data (Figure S4). The results from dbRDA analyses were similar for both flow cytometry and 16S rRNA gene sequencing data (Figure 2C,D) where samples clustered by site type and where total chlorine (copper, NEG), temperature, and manganese (copper, POS, and magnesium, POS) were significantly associated with differences in microbial community structure between the site types (Table S8A) [via a permutation test for Bray–Curtis dbRDA, all *p* = 0.01–0.02 (Table S8B,C)]. Variance partitioning analysis identified total chlorine as the primary explanatory water chemistry parameter associated with differences in microbial community structure for both flow cytometric data (adj. *R*^2^ = 0.116) and 16S rRNA gene sequencing data (adj. *R*^2^ = 0.143), while temperature and manganese had smaller effect sizes (Table S8B,C). Total chlorine concentrations were significantly lower at the commercial building sites (0.51 ± 0.68 mg L^–1^) than at the residential household sites [1.57 ± 0.6 mg L^–1^; via a Mann–Whitney U test, *p* = 0.005 (Table S4A)], while temperature and selected metal concentrations, including manganese, were significantly increased [via a Mann–Whitney U test, *p* = 1.58 × 10^–5^ to 0.04 (Table S4A)], though still below the regulatory/advisory limits. Loss of disinfectant residual accompanied by increased temperatures during intermittent periods of stagnation is known to impact the microbial community structure,^11,20,47,48^ while increased metal concentrations at the commercial building sites likely are related to metal leaching from the piping materials or scale formation during stagnation and subsequent release.^49^

**Figure 3:**
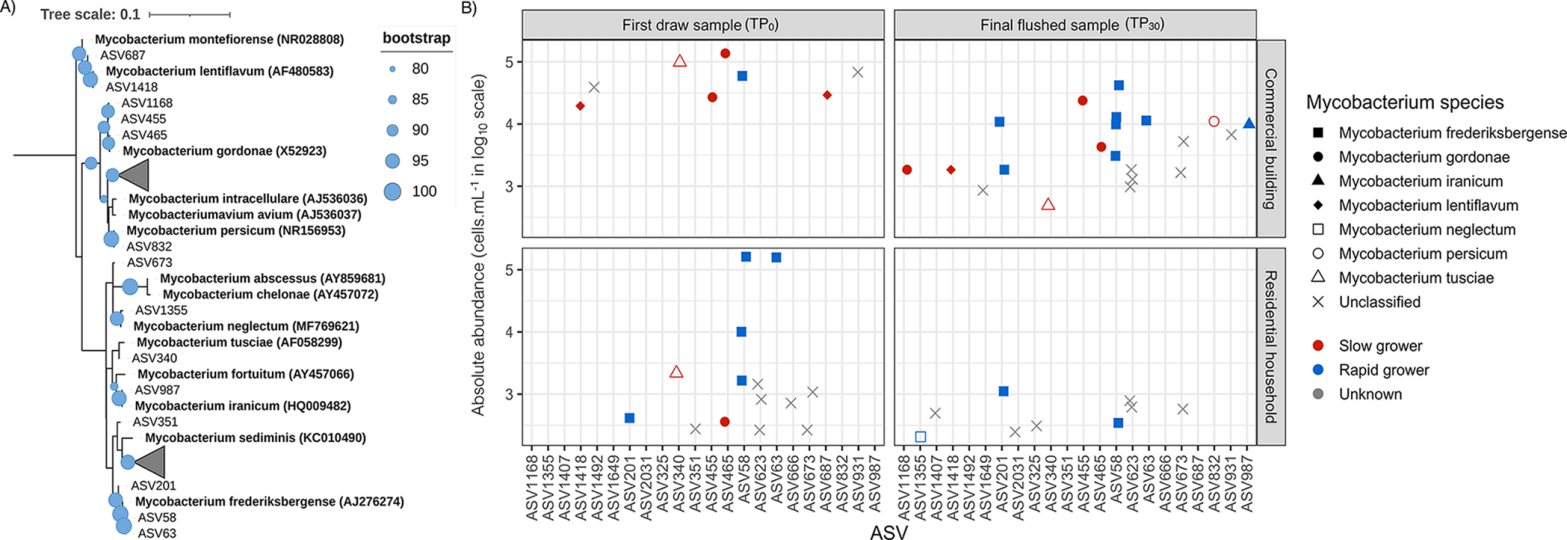
(A) Maximum likelihood phylogenetic tree showing the grouping of *Mycobacterium* ASVs with 16S rRNA gene sequence of *Mycobacterium* reference strains. Bootstrap analysis of 1000 replicates was performed, and bootstrap values were reported as percentages (depicted by size). (B) Scatter plot showing the absolute abundance on a log scale of 22 *Mycobacterium* ASVs detected in the TP_0_ and TP_30_ samples of the commercial buildings and residential households. *Mycobacterium* ASVs classified at the species level had >99% relatedness with reference *Mycobacterium* 16S rRNA strains (Table S6) and were grouped as slow growers (red) or rapid growers (blue).

While *Proteobacteria* constituted >75% of the microbial community for both site types (Figure S1A and Table S5), *Alphaproteobacteria* were more abundant in the commercial building TP_0_ samples [mean relative abundance (RA) = 54.52 ± 16.08%] while *Beta*- and *Gammaproteobacteria* were more abundant in the commercial building TP_30_ samples and residential household samples (Figure S1B and Table S5). *Pseudorhodoferax* (family Comamonadaceae) had notably higher abundance in the commercial building TP_0_ samples (mean RA = 11.62 ± 10.43%) compared to the commercial building TP_30_ samples (mean RA = 3.2 ± 3.5%) and residential household samples [mean RA = 0.01 ± 0.02 for both TP_0_ and TP_30_ samples (Figure S1D)]. Other genera linked to drinking water biofilms, including *Sphingomonas* (family Sphingomonadaceae) and *Hydrogenophaga* (family *Comamonadaceae*),^50−53^ as well as *Phreatobacter* (family *Phreatobacteraceae*) isolated from a water storage tank^54^ were also detected at higher RAs in the commercial building TP_0_ samples compared to the TP_30_ samples and residential household samples (Figure S1D and Table S5). *Nitrosomonas* and *Nitrospira* ASVs sharing >99% relatedness to *Nitrosomonas urea*, *Nitrosomonas oligotropha,* and *Nitrospira lenta* (Figure S1C, Table S5, Figure S2A,B, and Table S6) were also detected at higher abundances in commercial than in residential buildings. These species are associated with nitrification in chloraminated drinking water systems and building plumbing systems that accelerate chloramine decay and promote microbial growth.^55−57^ ASVs related to opportunistic pathogens *Legionella pneumophila, Pseudomonas aeruginosa*, and the *Mycobacterium avium* complex were not detected (Figure 3A and Figure S3A,B). *Mycobacterium* ASVs with >99% similarity to slow-growing *Mycobacterium* species, including *Mycobacterium gordonae*, *Mycobacterium tusciae*, *Mycobacterium lentiflavum*, and *Mycobacterium persicum* (Table S6), were more prevalent and abundant in the commercial building samples than in the residential household samples (Figure 3B and Table S7).

Concerns regarding opportunistic pathogen proliferation, specifically *L. pneumophila*, in building plumbing systems and potential exposure following reopening were heightened with the enforcement of COVID-19-related building closures.^22^ However, as reported here and elsewhere, multiple opportunistic pathogens of concern were either undetected or detected at very low levels.^16,47^ Systems with chloramine as a residual disinfectant typically do not experience *L. pneumophila* proliferation,^58,59^ even under stagnant conditions where levels are diminished or low;^16,47^ this likely explains their absence at commercial building sites post-reopening. Thus, the risk of exposure to these opportunistic waterborne pathogens post-reopening was minimal. Nonetheless, the extended stagnation in commercial buildings did result in bulk water communities in commercial locations that were distinct from residential households that were marked by low diversity and evenness, suggesting proliferation of taxa adapted to stagnant conditions. Indeed, the microbial community in the first draw as well as the final flush samples at the commercial locations was markedly enriched in bacterial taxa typically persistent in biofilm or low-flow environments or slow growers. For instance, *Pseudorhodofera, Sphingomonas, Hydrogenophaga*, and *Phreatobacter*-like species are often detected in drinking water biofilms^50−53^ and water storage.^16,47^ Similarly, slow-growing *Mycobacterium* species (*M. gordonae*, *M. lentiflavum*, etc.) as well as autotrophic nitrifiers were far more prevalent in commercial locations than in residential locations, indicating a substantial portion of the microbial communities in the commercial locations was dominated by slow-growing or typically biofilm-associated taxa.

### Flushing Profiles Presented Differential Impacts of Stagnation in Commercial Buildings and Residential Households That Varied with Phased Reopening

**Figure 4:**
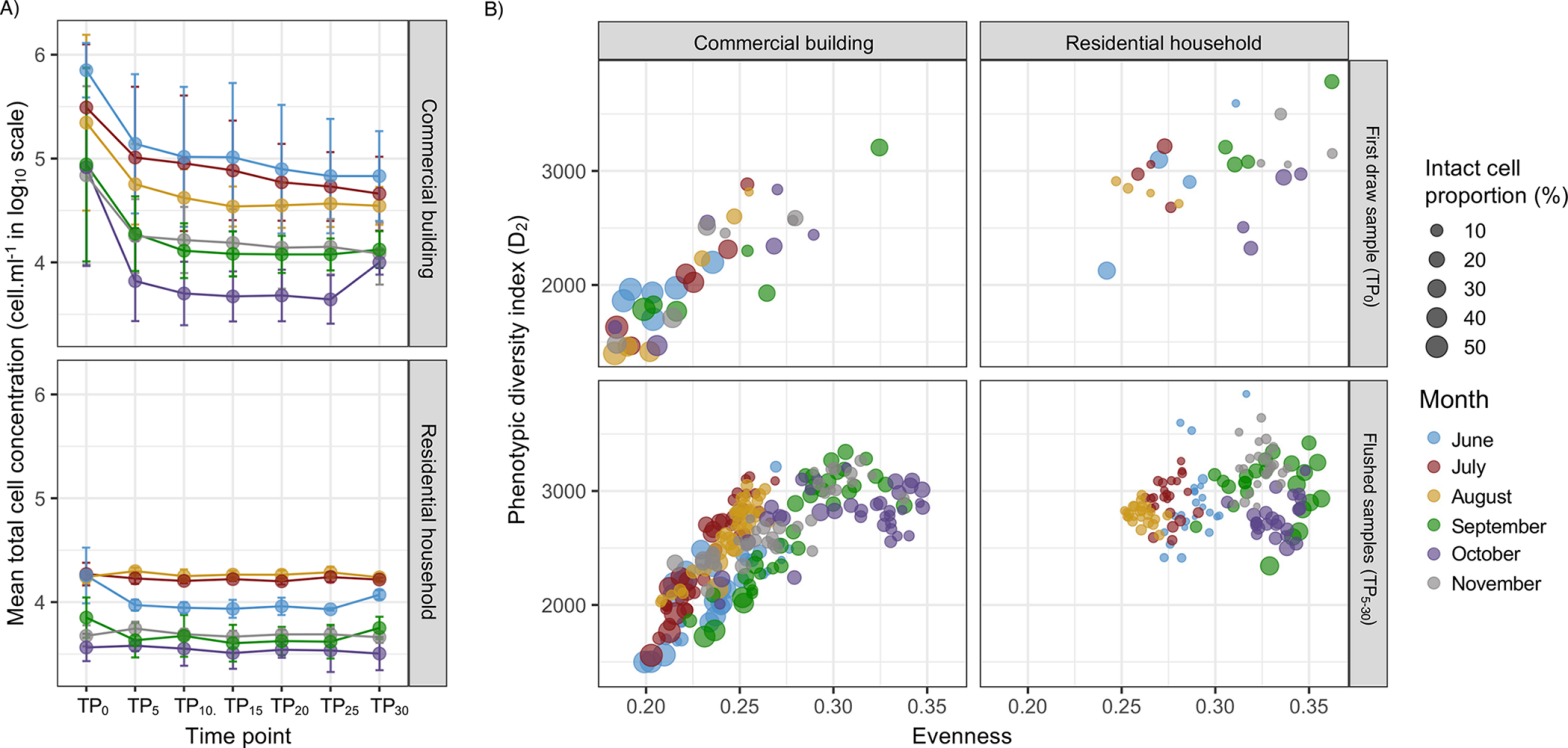
(A) Monthly flush profiles based on TCC measurements that were averaged for individual time points within each month across all commercial building and residential household sites. Bars represent the standard deviation, indicating the dispersion of individual TCC measures in relation to the mean. (B) Bubble plot showing intact cell proportions (depicted by size) with the phenotypic diversity index (*D*_2_) and evenness of monthly samples that were collected from the commercial building and residential household sites over 6 months. Panels from the top to bottom represent measures related to first draw samples (TP_0_), and subsequent samples (TP_5–30_), respectively.

This difference between the TCC of TP_0_ and TP_5–30_ samples at commercial buildings was 5–50-fold depending on the sampling month and was significantly higher than that observed at residential sampling sites [via a Mann–Whitney U test, most *p* = 0.02–0.006 (Table S9)]. Flushing at the residential household sites rapidly mitigated stagnation-induced effects compared to the commercial building sites with TCC measures stabilizing between 10^3^ and 10^4^ cells mL^–1^ after a 10 L flushing in 5 min (Figure 4A and Table S10). Similarly, the temperature is measured at the residential household sites stabilized after an initial decrease between TP_0_ and TP_5_ (Figure S5 and Table S10). These congruent patterns imply that freshwater from the distribution main reached the point of use at residential household sites within the first 5 min of flushing. In contrast, TCC measures of the commercial building sites did not stabilize during the flushing period and were associated with large 10–100-fold differences between the TP_0_ and TP_5_ time points, while successive time points had smaller differences that decreased in sequential order (Figure 4A and Table S10). These observations were associated with increased temperature measures that did not stabilize during the flushing period (Figure S5 and Table S10). This suggests that freshwater from the distribution main did not reach the point of use fixtures of the commercial building sites over the flush period.

The TP_0_ samples of the commercial building and residential household sites had greater intact cell proportions and less diverse and even microbial communities compared to the TP_5–30_ samples (Figure 4B and Tables S9 and S11). These differences were greater between the TP_0_ and TP_5–30_ samples of the commercial building sites than the residential household sites with dissimilarities averaging 33 ± 3% and 26 ± 2%, respectively (Table S11). The 16S rRNA gene sequencing results were concordant with flow cytometric observations with differences between TP_0_ and TP_5–30_ samples for commercial building samples (*d*_BC_ = 0.65 ± 0.07) being notably higher than for residential household sites [*d*_BC_ = 0.36 ± 0.04 (Table S12)]. Differences in microbial community structure between the TP_0_ and TP_30_ samples of the residential household sites were insignificant on the basis of PERMANOVA [all *p* = 0.26–0.96 (Table S12)]. Compositional profiles at the phylum, class, family, and genus levels confirmed higher degrees of similarity between the TP_0_ and TP_30_ samples of the residential household sites (Figure S6). Likewise, a high degree of community similarity was observed between the TP_0_ and TP_30_ samples of the commercial building sites that were collected during the first 2 months post-building reopening (i.e., June and July), with insignificant differences in community structure on the basis of PERMANOVA [all *p* = 0.08–0.14 (Table S12)]. In contrast to residential household sites, these similarities were likely due to the impact of extended stagnation that was not sufficiently remedied by short-term flushing and likely exacerbated by large-scale building size and plumbing complexity. The microbial communities of the commercial building TP_0_ and TP_30_ samples that were collected in subsequent months (i.e., August, September, October, and November) were significantly different [via PERMANOVA, all *p* = 0.01–0.02 (Table S12)]. These findings were confirmed by shifts in family- and genus-level compositional profiles of the commercial building TP_0_ and TP_30_ samples (Figure S6). These differences were likely due to the reduced impact of stagnation with monthly water demand recovery.

**Figure 5:**
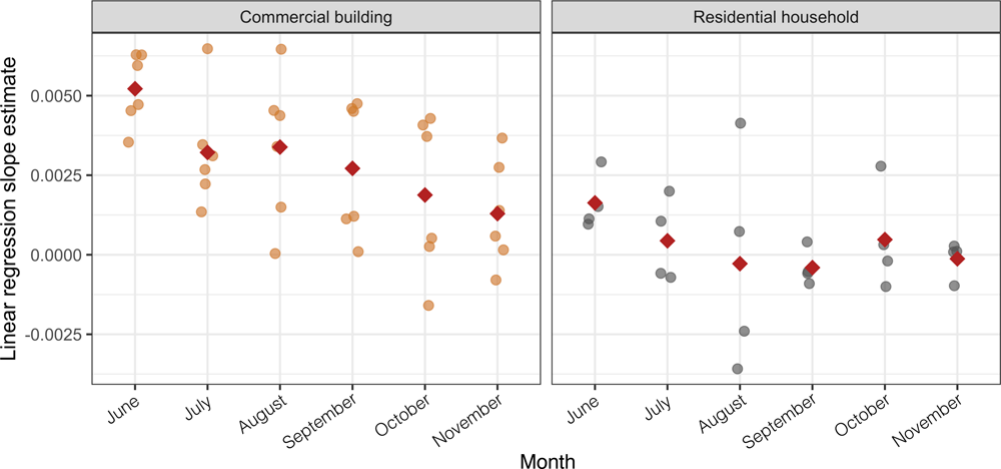
Linear regression slope estimated for Bray–Curtis dissimilarity distances of all time point combinations over the flush duration for commercial building (orange) and residential household (gray) sites. The red diamond represents the mean calculated across the linear regression slope estimates for each month.

We used flow cytometry-based pairwise Bray–Curtis dissimilarities between all time points within a flushing profile to determine the extent of change in community composition during a flushing profile and compared this change between sampling locations across months (Table S13). While microbial communities changed with the flushing period at both site types, flush duration was more strongly correlated with microbial community change at the commercial building sites and the change was significantly larger than at residential locations where the changes over flushing period were largely insignificant (Table S13). This observation could be associated with not only stagnation time but also building size and plumbing complexity. Interestingly, the extent of change over flushing period at the commercial building sites decreased in successive months, while demonstrating no significant trend at the residential household sites (Figure 5), suggesting that with successive building reopening and water demand recovery, flushing had a weaker impact on the changes in microbial communities.

Flushing replenishes disinfectant residual concentrations and reduces microbial loads in building plumbing systems^11,12,16,17,47^ but only temporarily improves water quality.^16,17,47^ For example, Hozalski et al. reported improved water quality in unoccupied nonresidential buildings after flushing for 30 min that returned to preflushed stagnation levels after 6–7 days.^16^ Similarly, Greenwald et al. reported that the changes in microbial concentrations postflushing were sustained for only a short period of time with cell counts returning to preflush levels within 7 days.^47^ In fact, they conclude that short-term flushing was insufficient in its ability to remediate the effects of long-term stagnation. The findings of our study are largely consistent with these reports in that while flushing over the short term does reduce cell counts, a long-term sustained increase in water demand over a period of months has a more substantive restorative impact. For instance, while flushing demonstrated a similar rate of change in community structure over the flushing period (i.e., regression slope in Figure 5) for residential locations, it took several months for the rate of change in community structure at commercial locations to become like that of the residential locations. Thus, while our study demonstrates consistent results with recent work on the short-lived benefits of flushing post-extended stagnation,^16,47^ we also demonstrate that a sustained and gradual increase in water demand plays an important role in the recovery of building plumbing-associated microbial communities.

### Microbial Communities of the Commercial Buildings Became More Similar to Those of Residential Households with the Relaxation of Building Closures

**Figure 6:**
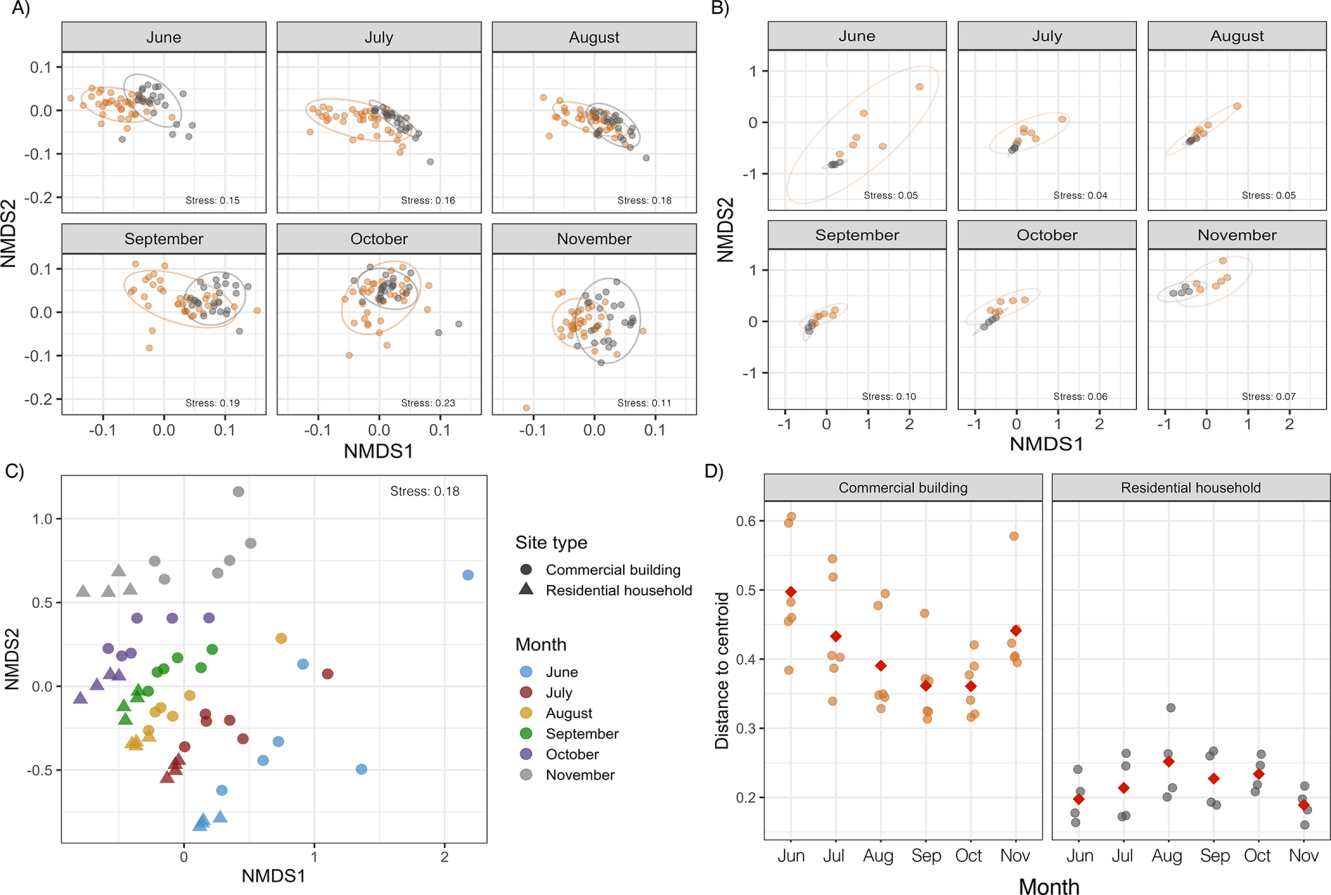
(A and B) NMDS plot illustrating differences in microbial community composition between samples of commercial buildings (orange) and residential households (gray) sites, using Bray–Curtis dissimilarity distances on flow cytometric fingerprinting and 16S rRNA gene sequencing data, respectively. The ellipse is drawn at a 95% confidence limit. (C) NMDS plot based on Bray–Curtis dissimilarity distances on 16S rRNA sequencing data illustrating differences in microbial community composition between TP_30_ samples of commercial buildings (●) and residential households (▲) between months. (D) Structure-based Bray–Curtis distance to the group centroid of commercial building and residential household microbial communities grouped by month. The red diamond represents the mean centroid calculated within each month.

The TP_5–30_ samples clustered by site type in June (Figure 6A); here, site type explained approximately 28% of the variation in the microbial community structure [via PERMANOVA, *F*(1, 58) = 22.41, *R*^2^ = 0.28, and *p* = 0.001]. The distinct clustering of commercial building and residential household samples decreased in successive months, and the contribution of site type on microbial community structure, while significant, was less pronounced [all *p* = 0.001–0.003 (Table S14A)]. June commercial building and residential household samples presented the greatest dissimilarity in community structure (*d*_BC_ = 0.33 ± 0.07) and were significantly more dissimilar when compared to the other months [via a Tukey HSD test, all *p* = 6.61 × 10^–11^ to 4.27 × 10^–3^ (Figure S14B)]. Interestingly, average dissimilarities gradually decreased over time with October and November commercial building and residential household samples exhibiting the lowest dissimilarities in community structure [*d*_BC_ = 0.27 ± 0.06 and 0.28 ± 0.05 for October and November, respectively (Table S14A)]. Comparable observations were made with 16S rRNA gene sequencing data of the final samples (TP_30_) (Figure 6B). Distinct clustering of the commercial building and residential household samples in June was significant, and site type explained approximately 28% of the variation in community structure [via PERMANOVA, *F*(1, 8) = 3.08, *R*^2^ = 0.28, and *p* = 0.014 (Table S15)]. The microbial communities of the commercial building and residential households exhibited higher similarities in community structure in July (*d*_BC_ = 0.63 ± 0.17), August (*d*_BC_ = 0.58 ± 0.14), September (*d*_BC_ = 0.54 ± 0.11), October (*d*_BC_ = 0.56 ± 0.19), and November (*d*_BC_ = 0.66 ± 0.19) relative to June (*d*_BC_ = 0.72 ± 0.20) (Figure 6B). These increases in similarity are likely related to the weakened impact of extended stagnation with monthly water demand recovery. The cause of the increase in dissimilarity between commercial and residential locations in November relative to July–October, while not definitive, may be associated with a change in environmental conditions compared to the previous months [i.e., lower water temperatures (Figure S5)]. In addition, the differences in β dispersivity were assessed to estimate the variation in microbial communities grouped by site type within each month (Figure 6C). The residential household samples consistently demonstrated low β dispersivity, suggesting that the microbial communities at residential locations were largely similar to each other; this despite changes temporal changes in microbial community structure [Tukey HSD test, all *p* > 0.05 (Table S16A,B)]. However, large differences in β dispersity were observed between the commercial building samples (Figure 6D and Table S16A,B) immediately after reopening in June (0.5 ± 0.08) with β dispersivity values decreasing over subsequent months (0.43–0.36) followed by a brief increase in November (0.44 ± 0.07) likely due to a change in environmental conditions.

This study was motivated by the primary concern of the proliferation of opportunistic waterborne pathogens, in particular *L. pneumophila*, in premise plumbing during the COVID-19 shutdown. The findings of our study are consistent with previous reports that the COVID-19 shutdown-related stagnation did not result in increased pathogen exposure risks in commercial buildings.^16,47^ Nonetheless, the extended period of this work post-reopening (6 months follow-up compared to 2 months in previous studies) as well as comparative analyses between commercial and residential locations provides a unique opportunity to build on findings from previous work.^16,47^ Our study design not only provides insights into temporal changes at these two site types post-reopening but also enables estimation of the differences in temporal changes between the two site types over time. The latter is critical to determine if the impact of extended stagnation on commercial building microbial communities diminishes with increasing building occupancy and water use; previous drinking water microbiology studies related to the COVID-19 shutdown largely focused on the impact of the shutdown itself and the ability to mitigate this impact by flushing^16,47^ and prevalence of *L. pneumophila* post-reopening across multiple drinking water systems.^59^

It is important to note here that pre-shutdown baseline measurements of microbial community composition at both the commercial and residential locations would have been ideal, and the lack of these data is an important limitation of this study. In addition, the changes in microbial community composition at both the commercial and residential locations are impacted by a host of factors, beyond just the shutdown and reopening dynamics, including very commonly reported temporal cycling and how these temporal changes are manifest in plumbing systems of different sizes and complexities.^13,38,48,53^ Nonetheless, we show that the shutdown resulted in not only impacts at commercial locations that could not be mitigated through short-term flushing, which is consistent with prior work, but also using multiple lines of evidence to show that the impact of this extended stagnation at commercial locations diminished over time. The three lines of evidence showing (1) temporal convergence between commercial and residential locations (Figure 6A,C), (2) temporal convergence between commercial locations as marked by a substantial decrease in β dispersivity (Figure 6D), and (3) a greater change during flushing immediately after reopening compared to later months (Figure 5) for commercial locations suggests that gradual and sustained increase in water post-reopening helped mitigate the impact of the COVID-19-related shutdown.

A key outcome of this study was that a gradual increase in water demand at the building level played a more substantial role than short-term fixture flushing in terms of recovery. While this study uses community convergence and the rate of change in community during flushing as indicators of recovery, it would be ideal to develop quantitative metrics for estimating the extent of recovery that directly inform water quality management practices. Such quantitative recovery metrics will very likely require integrating measurements of temporal dynamics of the drinking water microbiome achievable through long-term monitoring^60,61^ with building-specific water use patterns and plumbing design.

## Supporting Information Available

Figures and tables providing additional supporting data and output of statistical tests discussed herein (PDF)
