# Supplementary material for: Gradual Recovery of Building Plumbing-Associated Microbial Communities after Extended Periods of Altered Water Demand during the COVID-19 Pandemic
Source: Environ Sci Technol. 2023 Feb 16;57(8):3248–59. doi: 10.1021/acs.est.2c07333 (PMC9969676; doi:10.1021/acs.est.2c07333)
Supplement: Supplementary file 1 — es2c07333_si_001.pdf [file es2c07333_si_001.pdf]

Supplementary information

Gradual recovery of building plumbing-associated microbial communities after extended periods of altered water demand during the COVID-19 pandemic.

Solize Vosloo[1], Linxuan Huo[2], Umang Chauhan[1], Irmarie Cotto[1], Benjamin Gincley[1], Katherine J Vilardi[1], Bryan Yoon[1], Kaiqin Bian[2], Marco Gabrielli[3], Kelsey J Pieper[1], Aron Stubbins[1], and Ameet Pinto[2]*

1 Department of Civil and Environmental Engineering, Northeastern University, 360 Huntington Avenue, Boston, Massachusetts, 021115, USA

2 School of Civil and Environmental Engineering, Georgia Institute of Technology, 311 Ferst Drive Atlanta, Georgia 30318, USA

3 Dipartimento di Ingegneria Civile e Ambientale – Sezione Ambientale, Politecnico di Milano, Milan, Italy.

*Corresponding author: Ameet Pinto (ameet.pinto@ce.gatech.edu)

Table of contents

This document consists of 82 pages, including 6 figures and 16 tables

		Pages
1	Figures S1-S6	S2-S6
2	Tables S1-S16	S7-S82

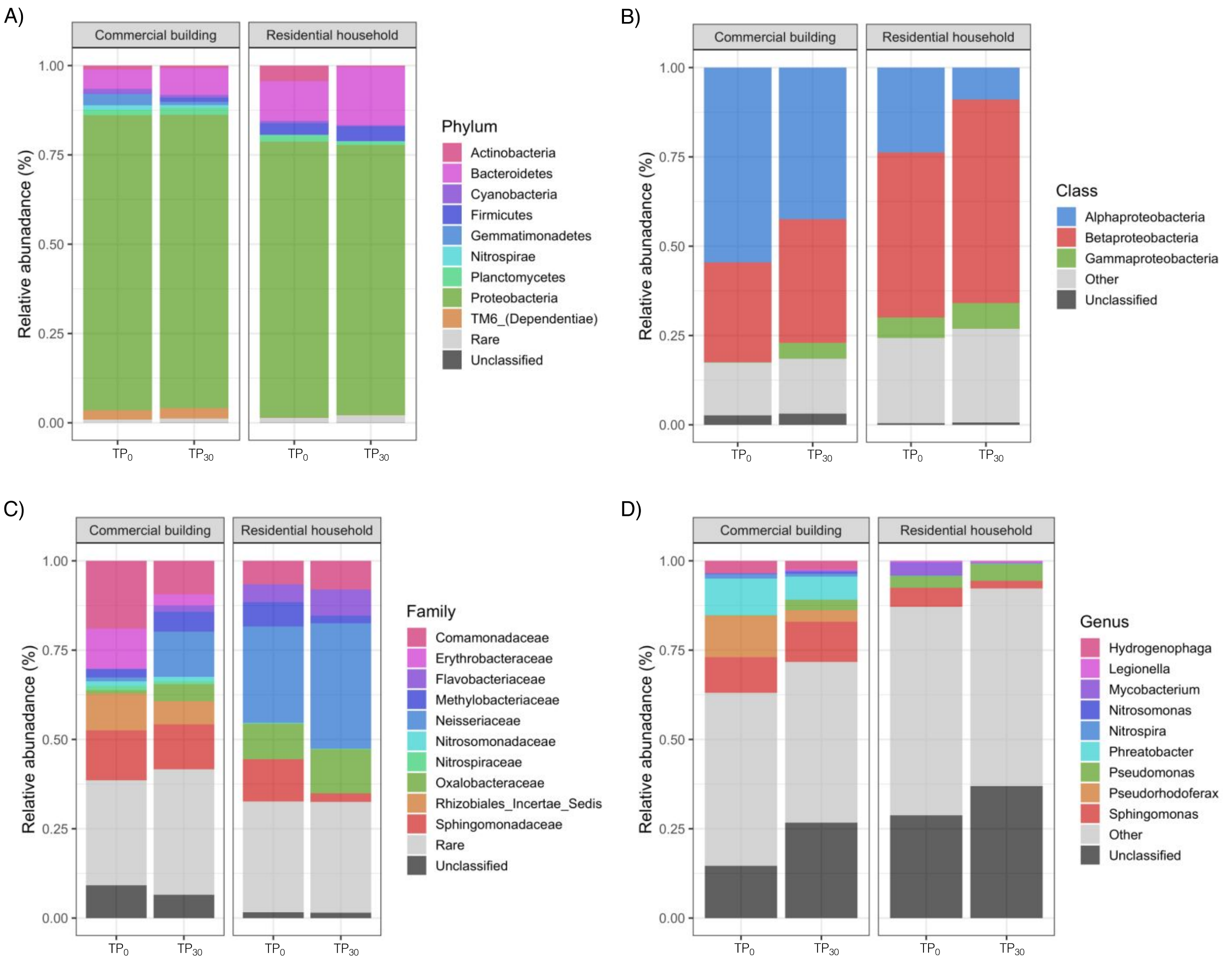

Figure S1: Stacked bar charts showing relative abundances at (A) phyla, (B) class, (C) family, and (D) genus levels that were classified from 16S rRNA gene sequences of TP_0 and TP_{30} commercial building and residential household June samples.

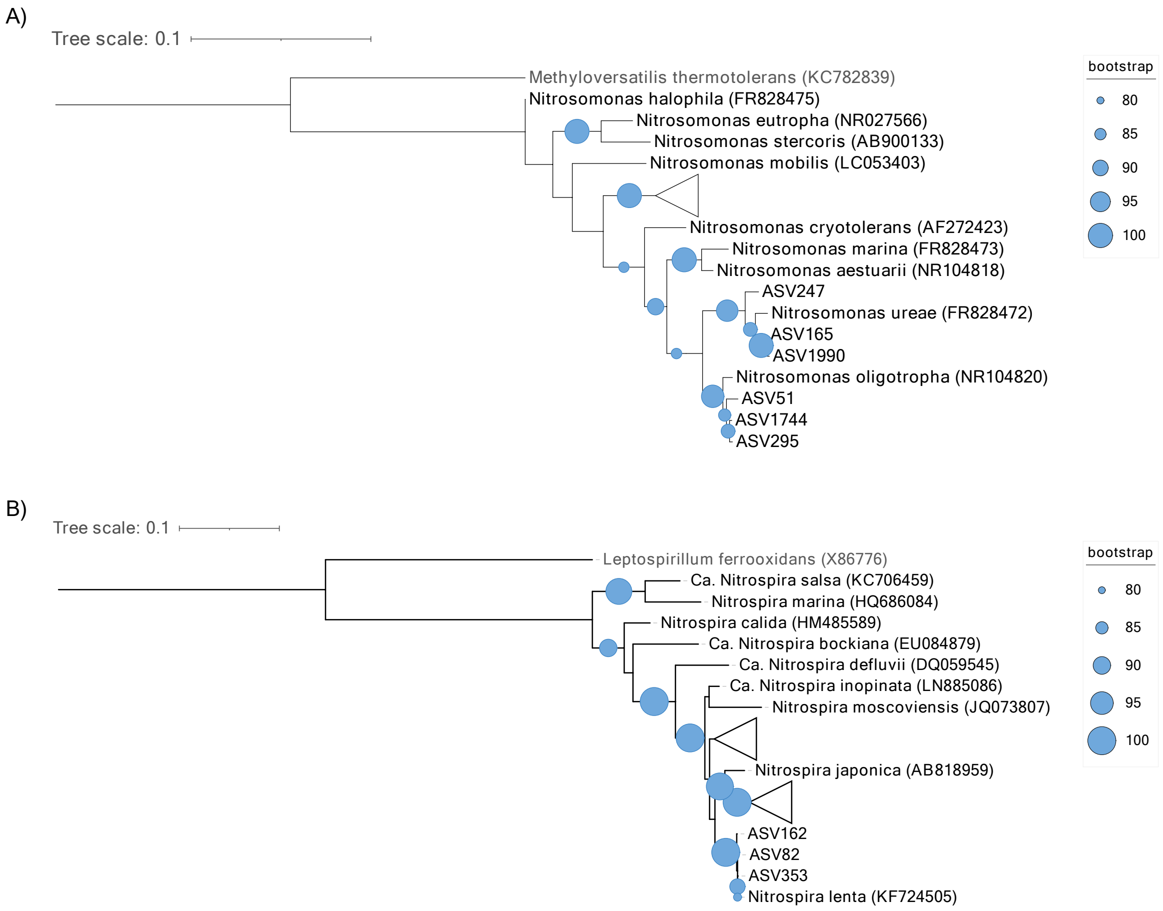

Figure S2: (A and B) Maximum likelihood phylogenetic tree showing the grouping of *Nitrosomonas* and *Nitrospira* ASVs with reference strains and outgroups. Bootstrap analysis of 1,000 replicates was performed and bootstrap values were reported as percentages (depicted by size).

A)

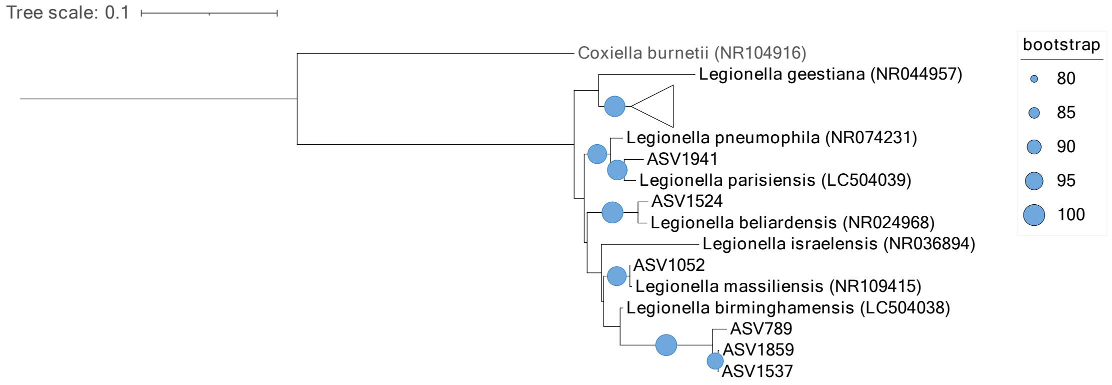

B)

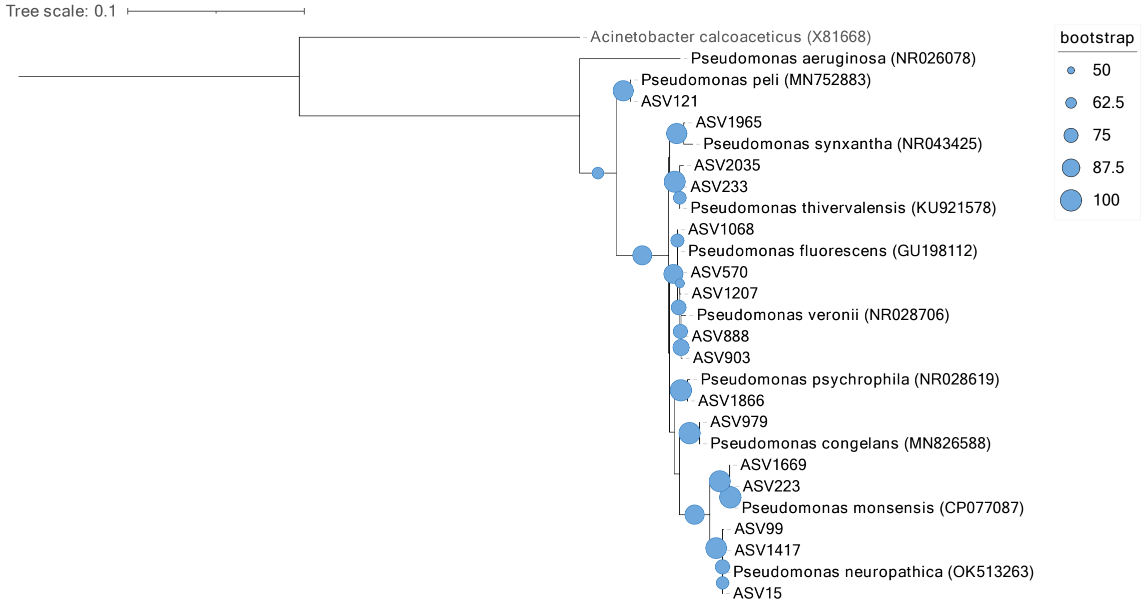

Figure S3: (A and B) Maximum likelihood phylogenetic tree showing the grouping of *Legionella* and *Pseudomonas* ASVs with reference strains and outgroups. Bootstrap analysis of 1,000 replicates was performed and bootstrap values were reported as percentages (depicted by size).

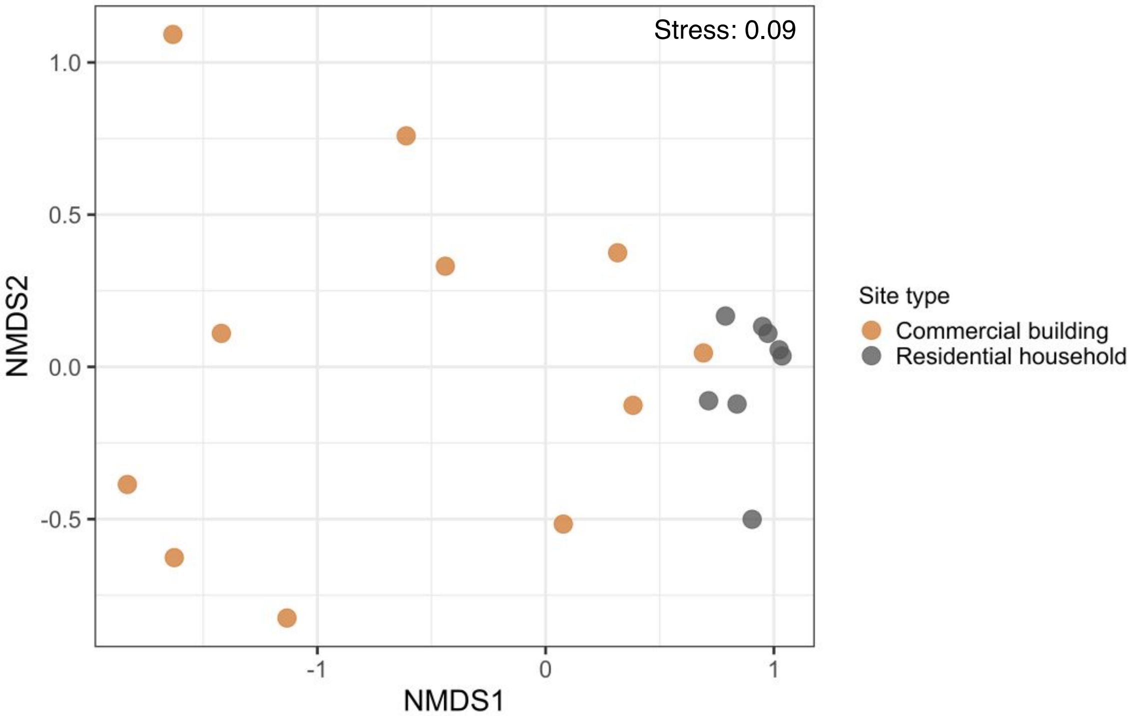

Figure S4: NMDS plot illustrating differences in microbial community composition between TP_0 and TP_{30} samples of commercial building and residential household sites using Bray-Curtis dissimilarity distances derived from 16S rRNA gene sequencing data.

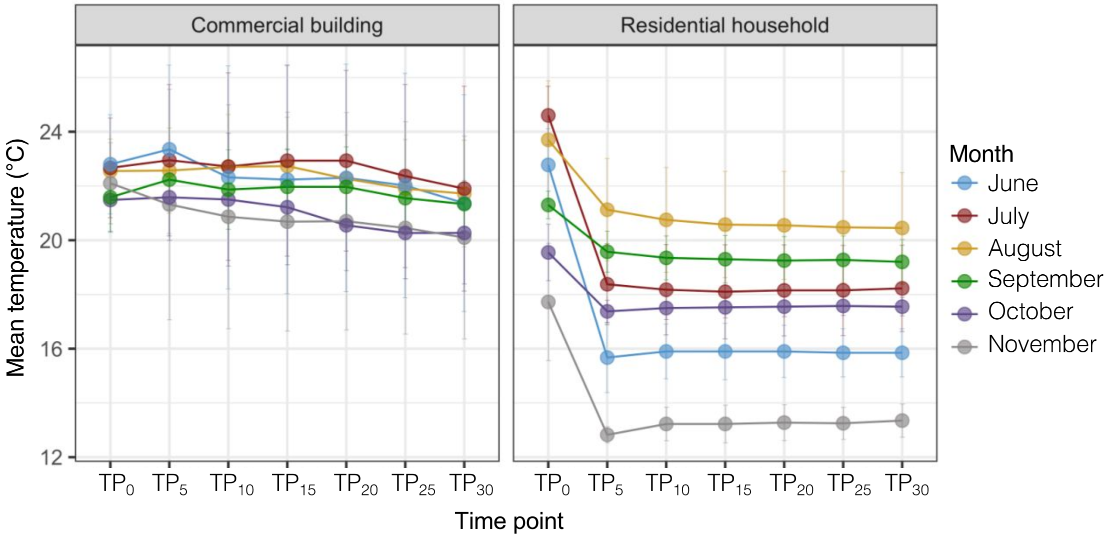

Figure S5: Monthly flush profiles based on temperature measurements that were averaged for individual time points (TP_0, 0 min; TP_5, 5 min; TP_{10}, 10 min; TP_{15}, 15 min; TP_{20}, 20 min; TP_{25}, 25 min; and TP_{30}, 30 min) within each month across all commercial building and residential household sites, respectively.

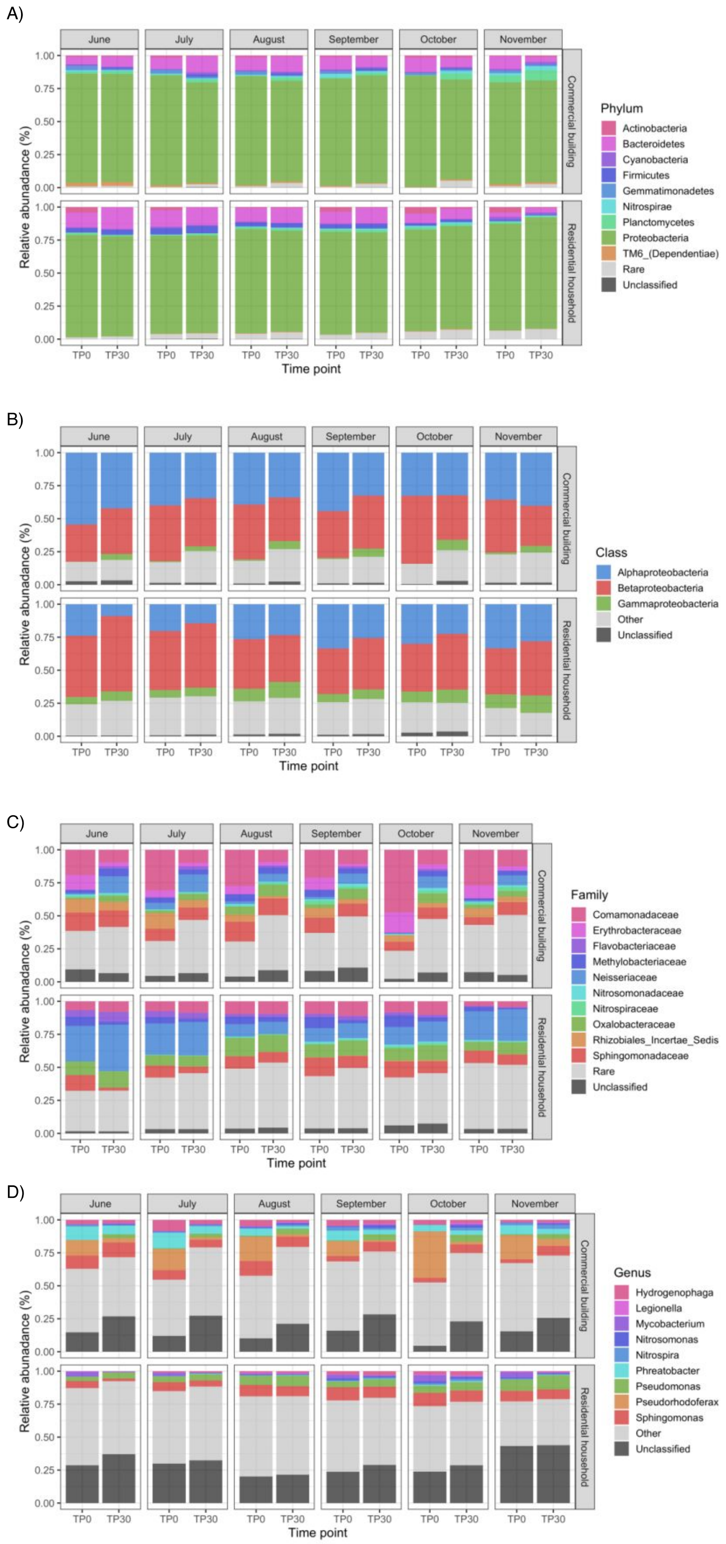

Figure S6: Stacked bar charts showing relative abundances at (A) phyla, (B) class, (C) family, and (D) genus levels that were classified from 16S rRNA gene sequences of TP_0 and TP_{30} commercial building and residential household June to November samples.

Table S1: Sample information including sampling logistics, water chemistry data, and direct and derived flow cytometric measures, and 16S rRNA gene sequencing statistics of 420 samples that were collected over six months from 10 sites situated in three commercial buildings and four residential households.

**Sample Id***	**Site type**	**Site**	**Tap type**	**Time point**	**Sampling date**	**Month**	**Season**	**Water usage (m^3/mth)**	**Sampling time (hh:mm)**	**Flush duration (min)**	**Flush flow rate ($L.min^{-1}$)**	**Flushed volume (L)**	**Volume collected (ml)**
COM1.1.1.3	COM	COM1.1	Bathroom	TP_0	2020-08-06	Aug	Summer	70950	5:45	0	0.00	0.00	2000
COM1.1.2.3	COM	COM1.1	Bathroom	TP_5	2020-08-06	Aug	Summer	70950	5:50	5	2.09	10.44	15
COM1.1.3.3	COM	COM1.1	Bathroom	TP_{10}	2020-08-06	Aug	Summer	70950	5:55	10	2.09	20.87	15
COM1.1.4.3	COM	COM1.1	Bathroom	TP_{15}	2020-08-06	Aug	Summer	70950	6:00	15	2.09	31.31	15
COM1.1.5.3	COM	COM1.1	Bathroom	TP_{20}	2020-08-06	Aug	Summer	70950	6:05	20	2.09	41.75	15
COM1.1.6.3	COM	COM1.1	Bathroom	TP_{25}	2020-08-06	Aug	Summer	70950	6:10	25	2.09	52.19	15
COM1.1.7.3	COM	COM1.1	Bathroom	TP_{30}	2020-08-06	Aug	Summer	70950	6:15	30	2.09	62.62	2000
COM1.2.1.3	COM	COM1.2	Laboratory	TP_0	2020-08-06	Aug	Summer	70950	6:20	0	0.00	0.00	2000
COM1.2.2.3	COM	COM1.2	Laboratory	TP_5	2020-08-06	Aug	Summer	70950	6:25	5	3.10	15.49	15
COM1.2.3.3	COM	COM1.2	Laboratory	TP_{10}	2020-08-06	Aug	Summer	70950	6:30	10	3.10	30.97	15
COM1.2.4.3	COM	COM1.2	Laboratory	TP_{15}	2020-08-06	Aug	Summer	70950	6:35	15	3.10	46.46	15
COM1.2.5.3	COM	COM1.2	Laboratory	TP_{20}	2020-08-06	Aug	Summer	70950	6:40	20	3.10	61.94	15
COM1.2.6.3	COM	COM1.2	Laboratory	TP_{25}	2020-08-06	Aug	Summer	70950	6:45	25	3.10	77.43	15
COM1.2.7.3	COM	COM1.2	Laboratory	TP_{30}	2020-08-06	Aug	Summer	70950	6:50	30	3.10	92.91	2000
COM2.1.1.3	COM	COM2.1	Laboratory	TP_0	2020-08-07	Aug	Summer	21190	8:18	0	0.00	0.00	2000
COM2.1.2.3	COM	COM2.1	Laboratory	TP_5	2020-08-07	Aug	Summer	21190	8:23	5	4.71	23.56	15
COM2.1.3.3	COM	COM2.1	Laboratory	TP_{10}	2020-08-07	Aug	Summer	21190	8:28	10	4.71	47.11	15
COM2.1.4.3	COM	COM2.1	Laboratory	TP_{15}	2020-08-07	Aug	Summer	21190	8:33	15	4.71	70.67	15
COM2.1.5.3	COM	COM2.1	Laboratory	TP_{20}	2020-08-07	Aug	Summer	21190	8:38	20	4.71	94.22	15
COM2.1.6.3	COM	COM2.1	Laboratory	TP_{25}	2020-08-07	Aug	Summer	21190	8:43	25	4.71	117.78	15
COM2.1.7.3	COM	COM2.1	Laboratory	TP_{30}	2020-08-07	Aug	Summer	21190	8:48	30	4.71	141.33	2000
COM2.2.1.3	COM	COM2.2	Kitchenette	TP_0	2020-08-07	Aug	Summer	21190	8:48	0	0.00	0.00	2000
COM2.2.2.3	COM	COM2.2	Kitchenette	TP_5	2020-08-07	Aug	Summer	21190	8:53	5	3.58	17.92	15

COM2.2.3.3	COM	COM2.2	Kitchenette	TP_{10}	2020-08-07	Aug	Summer	21190	8:58	10	3.58	35.85	15
COM2.2.4.3	COM	COM2.2	Kitchenette	TP_{15}	2020-08-07	Aug	Summer	21190	9:03	15	3.58	53.77	15
COM2.2.5.3	COM	COM2.2	Kitchenette	TP_{20}	2020-08-07	Aug	Summer	21190	9:08	20	3.58	71.69	15
COM2.2.6.3	COM	COM2.2	Kitchenette	TP_{25}	2020-08-07	Aug	Summer	21190	9:13	25	3.58	89.61	15
COM2.2.7.3	COM	COM2.2	Kitchenette	TP_{30}	2020-08-07	Aug	Summer	21190	9:18	30	3.58	107.54	2000
COM3.1.1.3	COM	COM3.1	Laboratory	TP_{0}	2020-08-07	Aug	Summer	91540	8:41	0	0.00	0.00	2000
COM3.1.2.3	COM	COM3.1	Laboratory	TP_{5}	2020-08-07	Aug	Summer	91540	8:46	5	7.06	35.29	15
COM3.1.3.3	COM	COM3.1	Laboratory	TP_{10}	2020-08-07	Aug	Summer	91540	8:51	10	7.06	70.59	15
COM3.1.4.3	COM	COM3.1	Laboratory	TP_{15}	2020-08-07	Aug	Summer	91540	8:56	15	7.06	105.88	15
COM3.1.5.3	COM	COM3.1	Laboratory	TP_{20}	2020-08-07	Aug	Summer	91540	9:01	20	7.06	141.18	15
COM3.1.6.3	COM	COM3.1	Laboratory	TP_{25}	2020-08-07	Aug	Summer	91540	9:06	25	7.06	176.47	15
COM3.1.7.3	COM	COM3.1	Laboratory	TP_{30}	2020-08-07	Aug	Summer	91540	9:11	30	7.06	211.76	2000
COM3.2.1.3	COM	COM3.2	Kitchenette	TP_{0}	2020-08-07	Aug	Summer	91540	9:32	0	0.00	0.00	2000
COM3.2.2.3	COM	COM3.2	Kitchenette	TP_{5}	2020-08-07	Aug	Summer	91540	9:37	5	2.86	14.31	15
COM3.2.3.3	COM	COM3.2	Kitchenette	TP_{10}	2020-08-07	Aug	Summer	91540	9:42	10	2.86	28.62	15
COM3.2.4.3	COM	COM3.2	Kitchenette	TP_{15}	2020-08-07	Aug	Summer	91540	9:47	15	2.86	42.93	15
COM3.2.5.3	COM	COM3.2	Kitchenette	TP_{20}	2020-08-07	Aug	Summer	91540	9:52	20	2.86	57.24	15
COM3.2.6.3	COM	COM3.2	Kitchenette	TP_{25}	2020-08-07	Aug	Summer	91540	9:57	25	2.86	71.54	15
COM3.2.7.3	COM	COM3.2	Kitchenette	TP_{30}	2020-08-07	Aug	Summer	91540	10:02	30	2.86	85.85	2000
RES1.1.3	RES	RES1	Kitchen	TP_{0}	2020-08-06	Aug	Summer	1081	9:06	0	0.00	0.00	2000
RES1.2.3	RES	RES1	Kitchen	TP_{5}	2020-08-06	Aug	Summer	1081	9:11	5	6.67	33.33	15
RES1.3.3	RES	RES1	Kitchen	TP_{10}	2020-08-06	Aug	Summer	1081	9:16	10	6.67	66.67	15
RES1.4.3	RES	RES1	Kitchen	TP_{15}	2020-08-06	Aug	Summer	1081	9:21	15	6.67	100.00	15
RES1.5.3	RES	RES1	Kitchen	TP_{20}	2020-08-06	Aug	Summer	1081	9:26	20	6.67	133.33	15
RES1.6.3	RES	RES1	Kitchen	TP_{25}	2020-08-06	Aug	Summer	1081	9:31	25	6.67	166.67	15
RES1.7.3	RES	RES1	Kitchen	TP_{30}	2020-08-06	Aug	Summer	1081	9:36	30	6.67	200.00	2000
RES2.1.3	RES	RES2	Bathroom	TP_{0}	2020-08-06	Aug	Summer	1693	8:55	0	0.00	0.00	2000
RES2.2.3	RES	RES2	Bathroom	TP_{5}	2020-08-06	Aug	Summer	1693	9:00	5	2.68	13.41	15
RES2.3.3	RES	RES2	Bathroom	TP_{10}	2020-08-06	Aug	Summer	1693	9:05	10	2.68	26.82	15
RES2.4.3	RES	RES2	Bathroom	TP_{15}	2020-08-06	Aug	Summer	1693	9:10	15	2.68	40.23	15
RES2.5.3	RES	RES2	Bathroom	TP_{20}	2020-08-06	Aug	Summer	1693	9:15	20	2.68	53.64	15

RES2.6.3	RES	RES2	Bathroom	TP_{25}	2020-08-06	Aug	Summer	1693	9:20	25	2.68	67.05	15
RES2.7.3	RES	RES2	Bathroom	TP_{30}	2020-08-06	Aug	Summer	1693	9:25	30	2.68	80.45	2000
RES3.1.3	RES	RES3	Bathroom	TP_{0}	2020-08-06	Aug	Summer	856	6:46	0	0.00	0.00	2000
RES3.2.3	RES	RES3	Bathroom	TP_{5}	2020-08-06	Aug	Summer	856	6:51	5	2.04	10.21	15
RES3.3.3	RES	RES3	Bathroom	TP_{10}	2020-08-06	Aug	Summer	856	6:56	10	2.04	20.43	15
RES3.4.3	RES	RES3	Bathroom	TP_{15}	2020-08-06	Aug	Summer	856	7:01	15	2.04	30.64	15
RES3.5.3	RES	RES3	Bathroom	TP_{20}	2020-08-06	Aug	Summer	856	7:06	20	2.04	40.85	15
RES3.6.3	RES	RES3	Bathroom	TP_{25}	2020-08-06	Aug	Summer	856	7:11	25	2.04	51.07	15
RES3.7.3	RES	RES3	Bathroom	TP_{30}	2020-08-06	Aug	Summer	856	7:16	30	2.04	61.28	2000
RES4.1.3	RES	RES4	Kitchen	TP_{0}	2020-08-06	Aug	Summer	1680	7:25	0	0.00	0.00	2000
RES4.2.3	RES	RES4	Kitchen	TP_{5}	2020-08-06	Aug	Summer	1680	7:30	5	3.00	15.00	15
RES4.3.3	RES	RES4	Kitchen	TP_{10}	2020-08-06	Aug	Summer	1680	7:35	10	3.00	30.00	15
RES4.4.3	RES	RES4	Kitchen	TP_{15}	2020-08-06	Aug	Summer	1680	7:40	15	3.00	45.00	15
RES4.5.3	RES	RES4	Kitchen	TP_{20}	2020-08-06	Aug	Summer	1680	7:45	20	3.00	60.00	15
RES4.6.3	RES	RES4	Kitchen	TP_{25}	2020-08-06	Aug	Summer	1680	7:50	25	3.00	75.00	15
RES4.7.3	RES	RES4	Kitchen	TP_{30}	2020-08-06	Aug	Summer	1680	7:55	30	3.00	90.00	2000
COM1.1.1.2	COM	COM1.1	Bathroom	TP_{0}	2020-07-02	Jul	Summer	71750	6:36	0	0.00	0.00	2000
COM1.1.2.2	COM	COM1.1	Bathroom	TP_{5}	2020-07-02	Jul	Summer	71750	6:41	5	2.47	12.34	15
COM1.1.3.2	COM	COM1.1	Bathroom	TP_{10}	2020-07-02	Jul	Summer	71750	6:46	10	2.47	24.67	15
COM1.1.4.2	COM	COM1.1	Bathroom	TP_{15}	2020-07-02	Jul	Summer	71750	6:51	15	2.47	37.01	15
COM1.1.5.2	COM	COM1.1	Bathroom	TP_{20}	2020-07-02	Jul	Summer	71750	6:56	20	2.47	49.35	15
COM1.1.6.2	COM	COM1.1	Bathroom	TP_{25}	2020-07-02	Jul	Summer	71750	7:01	25	2.47	61.68	15
COM1.1.7.2	COM	COM1.1	Bathroom	TP_{30}	2020-07-02	Jul	Summer	71750	7:06	30	2.47	74.02	2000
COM1.2.1.2	COM	COM1.2	Laboratory	TP_{0}	2020-07-02	Jul	Summer	71750	10:10	0	0.00	0.00	2000
COM1.2.2.2	COM	COM1.2	Laboratory	TP_{5}	2020-07-02	Jul	Summer	71750	10:15	5	3.31	16.55	15
COM1.2.3.2	COM	COM1.2	Laboratory	TP_{10}	2020-07-02	Jul	Summer	71750	10:20	10	3.31	33.11	15
COM1.2.4.2	COM	COM1.2	Laboratory	TP_{15}	2020-07-02	Jul	Summer	71750	10:25	15	3.31	49.66	15
COM1.2.5.2	COM	COM1.2	Laboratory	TP_{20}	2020-07-02	Jul	Summer	71750	10:30	20	3.31	66.22	15
COM1.2.6.2	COM	COM1.2	Laboratory	TP_{25}	2020-07-02	Jul	Summer	71750	10:35	25	3.31	82.77	15
COM1.2.7.2	COM	COM1.2	Laboratory	TP_{30}	2020-07-02	Jul	Summer	71750	10:40	30	3.31	99.32	2000
COM2.1.1.2	COM	COM2.1	Laboratory	TP_{0}	2020-07-03	Jul	Summer	15470	5:56	0	0.00	0.00	2000

COM2.1.2.2	COM	COM2.1	Laboratory	TP_5	2020-07-03	Jul	Summer	15470	6:01	5	2.16	10.78	15
COM2.1.3.2	COM	COM2.1	Laboratory	TP_{10}	2020-07-03	Jul	Summer	15470	6:06	10	2.16	21.56	15
COM2.1.4.2	COM	COM2.1	Laboratory	TP_{15}	2020-07-03	Jul	Summer	15470	6:11	15	2.16	32.34	15
COM2.1.5.2	COM	COM2.1	Laboratory	TP_{20}	2020-07-03	Jul	Summer	15470	6:16	20	2.16	43.12	15
COM2.1.6.2	COM	COM2.1	Laboratory	TP_{25}	2020-07-03	Jul	Summer	15470	6:21	25	2.16	53.90	15
COM2.1.7.2	COM	COM2.1	Laboratory	TP_{30}	2020-07-03	Jul	Summer	15470	6:26	30	2.16	64.68	2000
COM2.2.1.2	COM	COM2.2	Ktchenette	TP_0	2020-07-03	Jul	Summer	15470	7:37	0	0.00	0.00	2000
COM2.2.2.2	COM	COM2.2	Ktchenette	TP_5	2020-07-03	Jul	Summer	15470	7:42	5	4.63	23.17	15
COM2.2.3.2	COM	COM2.2	Ktchenette	TP_{10}	2020-07-03	Jul	Summer	15470	7:47	10	4.63	46.34	15
COM2.2.4.2	COM	COM2.2	Ktchenette	TP_{15}	2020-07-03	Jul	Summer	15470	7:52	15	4.63	69.52	15
COM2.2.5.2	COM	COM2.2	Ktchenette	TP_{20}	2020-07-03	Jul	Summer	15470	7:57	20	4.63	92.69	15
COM2.2.6.2	COM	COM2.2	Ktchenette	TP_{25}	2020-07-03	Jul	Summer	15470	8:02	25	4.63	115.86	15
COM2.2.7.2	COM	COM2.2	Ktchenette	TP_{30}	2020-07-03	Jul	Summer	15470	8:07	30	4.63	139.03	2000
COM3.1.1.2	COM	COM3.1	Laboratory	TP_0	2020-07-03	Jul	Summer	37840	7:59	0	0.00	0.00	2000
COM3.1.2.2	COM	COM3.1	Laboratory	TP_5	2020-07-03	Jul	Summer	37840	8:04	5	5.11	25.57	15
COM3.1.3.2	COM	COM3.1	Laboratory	TP_{10}	2020-07-03	Jul	Summer	37840	8:09	10	5.11	51.13	15
COM3.1.4.2	COM	COM3.1	Laboratory	TP_{15}	2020-07-03	Jul	Summer	37840	8:14	15	5.11	76.70	15
COM3.1.5.2	COM	COM3.1	Laboratory	TP_{20}	2020-07-03	Jul	Summer	37840	8:19	20	5.11	102.26	15
COM3.1.6.2	COM	COM3.1	Laboratory	TP_{25}	2020-07-03	Jul	Summer	37840	8:24	25	5.11	127.83	15
COM3.1.7.2	COM	COM3.1	Laboratory	TP_{30}	2020-07-03	Jul	Summer	37840	8:29	30	5.11	153.39	2000
COM3.2.1.2	COM	COM3.2	Ktchenette	TP_0	2020-07-03	Jul	Summer	37840	9:02	0	0.00	0.00	2000
COM3.2.2.2	COM	COM3.2	Ktchenette	TP_5	2020-07-03	Jul	Summer	37840	9:07	5	5.34	26.71	15
COM3.2.3.2	COM	COM3.2	Ktchenette	TP_{10}	2020-07-03	Jul	Summer	37840	9:12	10	5.34	53.42	15
COM3.2.4.2	COM	COM3.2	Ktchenette	TP_{15}	2020-07-03	Jul	Summer	37840	9:17	15	5.34	80.12	15
COM3.2.5.2	COM	COM3.2	Ktchenette	TP_{20}	2020-07-03	Jul	Summer	37840	9:22	20	5.34	106.83	15
COM3.2.6.2	COM	COM3.2	Ktchenette	TP_{25}	2020-07-03	Jul	Summer	37840	9:27	25	5.34	133.54	15
COM3.2.7.2	COM	COM3.2	Ktchenette	TP_{30}	2020-07-03	Jul	Summer	37840	9:32	30	5.34	160.25	2000
RES1.1.2	RES	RES1	Kitchen	TP_0	2020-07-02	Jul	Summer	933	9:02	0	0.00	0.00	2000
RES1.2.2	RES	RES1	Kitchen	TP_5	2020-07-02	Jul	Summer	933	9:07	5	7.17	35.85	15
RES1.3.2	RES	RES1	Kitchen	TP_{10}	2020-07-02	Jul	Summer	933	9:12	10	7.17	71.69	15
RES1.4.2	RES	RES1	Kitchen	TP_{15}	2020-07-02	Jul	Summer	933	9:17	15	7.17	107.54	15

RES1.5.2	RES	RES1	Kitchen	TP_{20}	2020-07-02	Jul	Summer	933	9:22	20	7.17	143.38	15
RES1.6.2	RES	RES1	Kitchen	TP_{25}	2020-07-02	Jul	Summer	933	9:27	25	7.17	179.23	15
RES1.7.2	RES	RES1	Kitchen	TP_{30}	2020-07-02	Jul	Summer	933	9:32	30	7.17	215.07	2000
RES2.1.2	RES	RES2	Bathroom	TP_{0}	2020-07-02	Jul	Summer	1835	9:27	0	0.00	0.00	2000
RES2.2.2	RES	RES2	Bathroom	TP_{5}	2020-07-02	Jul	Summer	1835	9:32	5	2.74	13.72	15
RES2.3.2	RES	RES2	Bathroom	TP_{10}	2020-07-02	Jul	Summer	1835	9:37	10	2.74	27.43	15
RES2.4.2	RES	RES2	Bathroom	TP_{15}	2020-07-02	Jul	Summer	1835	9:42	15	2.74	41.15	15
RES2.5.2	RES	RES2	Bathroom	TP_{20}	2020-07-02	Jul	Summer	1835	9:47	20	2.74	54.86	15
RES2.6.2	RES	RES2	Bathroom	TP_{25}	2020-07-02	Jul	Summer	1835	9:52	25	2.74	68.58	15
RES2.7.2	RES	RES2	Bathroom	TP_{30}	2020-07-02	Jul	Summer	1835	9:57	30	2.74	82.29	2000
RES3.1.2	RES	RES3	Bathroom	TP_{0}	2020-07-02	Jul	Summer	1058	8:08	0	0.00	0.00	2000
RES3.2.2	RES	RES3	Bathroom	TP_{5}	2020-07-02	Jul	Summer	1058	8:13	5	5.11	25.57	15
RES3.3.2	RES	RES3	Bathroom	TP_{10}	2020-07-02	Jul	Summer	1058	8:18	10	5.11	51.13	15
RES3.4.2	RES	RES3	Bathroom	TP_{15}	2020-07-02	Jul	Summer	1058	8:23	15	5.11	76.70	15
RES3.5.2	RES	RES3	Bathroom	TP_{20}	2020-07-02	Jul	Summer	1058	8:28	20	5.11	102.26	15
RES3.6.2	RES	RES3	Bathroom	TP_{25}	2020-07-02	Jul	Summer	1058	8:33	25	5.11	127.83	15
RES3.7.2	RES	RES3	Bathroom	TP_{30}	2020-07-02	Jul	Summer	1058	8:38	30	5.11	153.39	2000
RES4.1.2	RES	RES4	Kitchen	TP_{0}	2020-07-02	Jul	Summer	1580	9:23	0	0.00	0.00	2000
RES4.2.2	RES	RES4	Kitchen	TP_{5}	2020-07-02	Jul	Summer	1580	9:28	5	8.19	40.97	15
RES4.3.2	RES	RES4	Kitchen	TP_{10}	2020-07-02	Jul	Summer	1580	9:33	10	8.19	81.93	15
RES4.4.2	RES	RES4	Kitchen	TP_{15}	2020-07-02	Jul	Summer	1580	9:38	15	8.19	122.90	15
RES4.5.2	RES	RES4	Kitchen	TP_{20}	2020-07-02	Jul	Summer	1580	9:43	20	8.19	163.87	15
RES4.6.2	RES	RES4	Kitchen	TP_{25}	2020-07-02	Jul	Summer	1580	9:48	25	8.19	204.83	15
RES4.7.2	RES	RES4	Kitchen	TP_{30}	2020-07-02	Jul	Summer	1580	9:53	30	8.19	245.80	2000
COM1.1.1.1	COM	COM1.1	Bathroom	TP_{0}	2020-06-04	Jun	Summer	17450	6:50	0	0.00	0.00	2000
COM1.1.2.1	COM	COM1.1	Bathroom	TP_{5}	2020-06-04	Jun	Summer	17450	6:55	5	2.03	10.15	15
COM1.1.3.1	COM	COM1.1	Bathroom	TP_{10}	2020-06-04	Jun	Summer	17450	7:00	10	2.03	20.30	15
COM1.1.4.1	COM	COM1.1	Bathroom	TP_{15}	2020-06-04	Jun	Summer	17450	7:05	15	2.03	30.45	15
COM1.1.5.1	COM	COM1.1	Bathroom	TP_{20}	2020-06-04	Jun	Summer	17450	7:10	20	2.03	40.60	15
COM1.1.6.1	COM	COM1.1	Bathroom	TP_{25}	2020-06-04	Jun	Summer	17450	7:15	25	2.03	50.75	15
COM1.1.7.1	COM	COM1.1	Bathroom	TP_{30}	2020-06-04	Jun	Summer	17450	7:20	30	2.03	60.90	2000

COM1.2.1.1	COM	COM1.2	Laboratory	TP_0	2020-06-04	Jun	Summer	17450	9:38	0	0.00	0.00	2000
COM1.2.2.1	COM	COM1.2	Laboratory	TP_5	2020-06-04	Jun	Summer	17450	9:43	5	4.17	20.85	15
COM1.2.3.1	COM	COM1.2	Laboratory	TP_{10}	2020-06-04	Jun	Summer	17450	9:48	10	4.17	41.70	15
COM1.2.4.1	COM	COM1.2	Laboratory	TP_{15}	2020-06-04	Jun	Summer	17450	9:53	15	4.17	62.55	15
COM1.2.5.1	COM	COM1.2	Laboratory	TP_{20}	2020-06-04	Jun	Summer	17450	9:58	20	4.17	83.40	15
COM1.2.6.1	COM	COM1.2	Laboratory	TP_{25}	2020-06-04	Jun	Summer	17450	10:03	25	4.17	104.25	15
COM1.2.7.1	COM	COM1.2	Laboratory	TP_{30}	2020-06-04	Jun	Summer	17450	10:08	30	4.17	125.10	2000
COM2.1.1.1	COM	COM2.1	Laboratory	TP_0	2020-06-05	Jun	Summer	8230	5:53	0	0.00	0.00	2000
COM2.1.2.1	COM	COM2.1	Laboratory	TP_5	2020-06-05	Jun	Summer	8230	5:58	5	3.51	17.55	15
COM2.1.3.1	COM	COM2.1	Laboratory	TP_{10}	2020-06-05	Jun	Summer	8230	6:03	10	3.51	35.10	15
COM2.1.4.1	COM	COM2.1	Laboratory	TP_{15}	2020-06-05	Jun	Summer	8230	6:08	15	3.51	52.65	15
COM2.1.5.1	COM	COM2.1	Laboratory	TP_{20}	2020-06-05	Jun	Summer	8230	6:13	20	3.51	70.20	15
COM2.1.6.1	COM	COM2.1	Laboratory	TP_{25}	2020-06-05	Jun	Summer	8230	6:18	25	3.51	87.75	15
COM2.1.7.1	COM	COM2.1	Laboratory	TP_{30}	2020-06-05	Jun	Summer	8230	6:23	30	3.51	105.30	2000
COM2.2.1.1	COM	COM2.2	Ktchenette	TP_0	2020-06-05	Jun	Summer	8230	8:00	0	0.00	0.00	2000
COM2.2.2.1	COM	COM2.2	Ktchenette	TP_5	2020-06-05	Jun	Summer	8230	8:05	5	5.11	25.55	15
COM2.2.3.1	COM	COM2.2	Ktchenette	TP_{10}	2020-06-05	Jun	Summer	8230	8:10	10	5.11	51.10	15
COM2.2.4.1	COM	COM2.2	Ktchenette	TP_{15}	2020-06-05	Jun	Summer	8230	8:15	15	5.11	76.65	15
COM2.2.5.1	COM	COM2.2	Ktchenette	TP_{20}	2020-06-05	Jun	Summer	8230	8:20	20	5.11	102.20	15
COM2.2.6.1	COM	COM2.2	Ktchenette	TP_{25}	2020-06-05	Jun	Summer	8230	8:25	25	5.11	127.75	15
COM2.2.7.1	COM	COM2.2	Ktchenette	TP_{30}	2020-06-05	Jun	Summer	8230	8:30	30	5.11	153.30	2000
COM3.1.1.1	COM	COM3.1	Laboratory	TP_0	2020-06-05	Jun	Summer	10910	6:29	0	0.00	0.00	2000
COM3.1.2.1	COM	COM3.1	Laboratory	TP_5	2020-06-05	Jun	Summer	10910	6:34	5	3.31	16.55	15
COM3.1.3.1	COM	COM3.1	Laboratory	TP_{10}	2020-06-05	Jun	Summer	10910	6:39	10	3.31	33.10	15
COM3.1.4.1	COM	COM3.1	Laboratory	TP_{15}	2020-06-05	Jun	Summer	10910	6:44	15	3.31	49.65	15
COM3.1.5.1	COM	COM3.1	Laboratory	TP_{20}	2020-06-05	Jun	Summer	10910	6:49	20	3.31	66.20	15
COM3.1.6.1	COM	COM3.1	Laboratory	TP_{25}	2020-06-05	Jun	Summer	10910	6:54	25	3.31	82.75	15
COM3.1.7.1	COM	COM3.1	Laboratory	TP_{30}	2020-06-05	Jun	Summer	10910	6:59	30	3.31	99.30	2000
COM3.2.1.1	COM	COM3.2	Ktchenette	TP_0	2020-06-05	Jun	Summer	10910	7:34	0	0.00	0.00	2000
COM3.2.2.1	COM	COM3.2	Ktchenette	TP_5	2020-06-05	Jun	Summer	10910	7:39	5	3.00	15.00	15
COM3.2.3.1	COM	COM3.2	Ktchenette	TP_{10}	2020-06-05	Jun	Summer	10910	7:44	10	3.00	30.00	15

COM3.2.4.1	COM	COM3.2	Ktchenette	TP_{15}	2020-06-05	Jun	Summer	10910	7:49	15	3.00	45.00	15
COM3.2.5.1	COM	COM3.2	Ktchenette	TP_{20}	2020-06-05	Jun	Summer	10910	7:54	20	3.00	60.00	15
COM3.2.6.1	COM	COM3.2	Ktchenette	TP_{25}	2020-06-05	Jun	Summer	10910	7:59	25	3.00	75.00	15
COM3.2.7.1	COM	COM3.2	Ktchenette	TP_{30}	2020-06-05	Jun	Summer	10910	8:04	30	3.00	90.00	2000
RES1.1.1	RES	RES1	Kitchen	TP_{0}	2020-06-04	Jun	Summer	974	7:15	0	0.00	0.00	2000
RES1.2.1	RES	RES1	Kitchen	TP_{5}	2020-06-04	Jun	Summer	974	7:20	5	2.30	11.50	15
RES1.3.1	RES	RES1	Kitchen	TP_{10}	2020-06-04	Jun	Summer	974	7:25	10	2.30	23.00	15
RES1.4.1	RES	RES1	Kitchen	TP_{15}	2020-06-04	Jun	Summer	974	7:30	15	2.30	34.50	15
RES1.5.1	RES	RES1	Kitchen	TP_{20}	2020-06-04	Jun	Summer	974	7:35	20	2.30	46.00	15
RES1.6.1	RES	RES1	Kitchen	TP_{25}	2020-06-04	Jun	Summer	974	7:40	25	2.30	57.50	15
RES1.7.1	RES	RES1	Kitchen	TP_{30}	2020-06-04	Jun	Summer	974	7:45	30	2.30	69.00	2000
RES2.1.1	RES	RES2	Bathroom	TP_{0}	2020-06-04	Jun	Summer	1987	7:29	0	0.00	0.00	2000
RES2.2.1	RES	RES2	Bathroom	TP_{5}	2020-06-04	Jun	Summer	1987	7:34	5	3.00	15.00	15
RES2.3.1	RES	RES2	Bathroom	TP_{10}	2020-06-04	Jun	Summer	1987	7:39	10	3.00	30.00	15
RES2.4.1	RES	RES2	Bathroom	TP_{15}	2020-06-04	Jun	Summer	1987	7:44	15	3.00	45.00	15
RES2.5.1	RES	RES2	Bathroom	TP_{20}	2020-06-04	Jun	Summer	1987	7:49	20	3.00	60.00	15
RES2.6.1	RES	RES2	Bathroom	TP_{25}	2020-06-04	Jun	Summer	1987	7:54	25	3.00	75.00	15
RES2.7.1	RES	RES2	Bathroom	TP_{30}	2020-06-04	Jun	Summer	1987	7:59	30	3.00	90.00	2000
RES3.1.1	RES	RES3	Bathroom	TP_{0}	2020-06-04	Jun	Summer	1160	6:20	0	0.00	0.00	2000
RES3.2.1	RES	RES3	Bathroom	TP_{5}	2020-06-04	Jun	Summer	1160	6:25	5	2.31	11.55	15
RES3.3.1	RES	RES3	Bathroom	TP_{10}	2020-06-04	Jun	Summer	1160	6:30	10	2.31	23.10	15
RES3.4.1	RES	RES3	Bathroom	TP_{15}	2020-06-04	Jun	Summer	1160	6:35	15	2.31	34.65	15
RES3.5.1	RES	RES3	Bathroom	TP_{20}	2020-06-04	Jun	Summer	1160	6:40	20	2.31	46.20	15
RES3.6.1	RES	RES3	Bathroom	TP_{25}	2020-06-04	Jun	Summer	1160	6:45	25	2.31	57.75	15
RES3.7.1	RES	RES3	Bathroom	TP_{30}	2020-06-04	Jun	Summer	1160	6:50	30	2.31	69.30	2000
RES4.1.1	RES	RES4	Kitchen	TP_{0}	2020-06-04	Jun	Summer	1510	8:04	0	0.00	0.00	2000
RES4.2.1	RES	RES4	Kitchen	TP_{5}	2020-06-04	Jun	Summer	1510	8:09	5	4.40	22.00	15
RES4.3.1	RES	RES4	Kitchen	TP_{10}	2020-06-04	Jun	Summer	1510	8:14	10	4.40	44.00	15
RES4.4.1	RES	RES4	Kitchen	TP_{15}	2020-06-04	Jun	Summer	1510	8:19	15	4.40	66.00	15
RES4.5.1	RES	RES4	Kitchen	TP_{20}	2020-06-04	Jun	Summer	1510	8:24	20	4.40	88.00	15
RES4.6.1	RES	RES4	Kitchen	TP_{25}	2020-06-04	Jun	Summer	1510	8:29	25	4.40	110.00	15

RES4.7.1	RES	RES4	Kitchen	TP_{30}	2020-06-04	Jun	Summer	1510	8:34	30	4.40	132.00	2000
COM1.1.1.6	COM	COM1.1	Bathroom	TP_0	2020-10-01	Nov	Fall	46000	5:30	0	0.00	0.00	2000
COM1.1.2.6	COM	COM1.1	Bathroom	TP_5	2020-11-05	Nov	Fall	46000	5:35	5	5.22	26.09	15
COM1.1.3.6	COM	COM1.1	Bathroom	TP_{10}	2020-11-05	Nov	Fall	46000	5:40	10	5.22	52.17	15
COM1.1.4.6	COM	COM1.1	Bathroom	TP_{15}	2020-11-05	Nov	Fall	46000	5:45	15	5.22	78.26	15
COM1.1.5.6	COM	COM1.1	Bathroom	TP_{20}	2020-11-05	Nov	Fall	46000	5:50	20	5.22	104.35	15
COM1.1.6.6	COM	COM1.1	Bathroom	TP_{25}	2020-11-05	Nov	Fall	46000	5:55	25	5.22	130.43	15
COM1.1.7.6	COM	COM1.1	Bathroom	TP_{30}	2020-11-05	Nov	Fall	46000	6:00	30	5.22	156.52	2000
COM1.2.1.6	COM	COM1.2	Laboratory	TP_0	2020-11-05	Nov	Fall	46000	7:07	0	0.00	0.00	2000
COM1.2.2.6	COM	COM1.2	Laboratory	TP_5	2020-11-05	Nov	Fall	46000	7:12	5	3.00	15.00	15
COM1.2.3.6	COM	COM1.2	Laboratory	TP_{10}	2020-11-05	Nov	Fall	46000	7:17	10	3.00	30.00	15
COM1.2.4.6	COM	COM1.2	Laboratory	TP_{15}	2020-11-05	Nov	Fall	46000	7:22	15	3.00	45.00	15
COM1.2.5.6	COM	COM1.2	Laboratory	TP_{20}	2020-11-05	Nov	Fall	46000	7:27	20	3.00	60.00	15
COM1.2.6.6	COM	COM1.2	Laboratory	TP_{25}	2020-11-05	Nov	Fall	46000	7:32	25	3.00	75.00	15
COM1.2.7.6	COM	COM1.2	Laboratory	TP_{30}	2020-11-05	Nov	Fall	46000	7:37	30	3.00	90.00	2000
COM2.1.1.6	COM	COM2.1	Laboratory	TP_0	2020-11-06	Nov	Fall	48020	5:38	0	0.00	0.00	2000
COM2.1.2.6	COM	COM2.1	Laboratory	TP_5	2020-11-06	Nov	Fall	48020	5:43	5	6.10	30.50	15
COM2.1.3.6	COM	COM2.1	Laboratory	TP_{10}	2020-11-06	Nov	Fall	48020	5:48	10	6.10	61.00	15
COM2.1.4.6	COM	COM2.1	Laboratory	TP_{15}	2020-11-06	Nov	Fall	48020	5:53	15	6.10	91.51	15
COM2.1.5.6	COM	COM2.1	Laboratory	TP_{20}	2020-11-06	Nov	Fall	48020	5:58	20	6.10	122.01	15
COM2.1.6.6	COM	COM2.1	Laboratory	TP_{25}	2020-11-06	Nov	Fall	48020	6:03	25	6.10	152.51	15
COM2.1.7.6	COM	COM2.1	Laboratory	TP_{30}	2020-11-06	Nov	Fall	48020	6:08	30	6.10	183.01	2000
COM2.2.1.6	COM	COM2.2	Ktchenette	TP_0	2020-11-06	Nov	Fall	48020	8:04	0	0.00	0.00	2000
COM2.2.2.6	COM	COM2.2	Ktchenette	TP_5	2020-11-06	Nov	Fall	48020	8:09	5	4.10	20.52	15
COM2.2.3.6	COM	COM2.2	Ktchenette	TP_{10}	2020-11-06	Nov	Fall	48020	8:14	10	4.10	41.03	15
COM2.2.4.6	COM	COM2.2	Ktchenette	TP_{15}	2020-11-06	Nov	Fall	48020	8:19	15	4.10	61.55	15
COM2.2.5.6	COM	COM2.2	Ktchenette	TP_{20}	2020-11-06	Nov	Fall	48020	8:24	20	4.10	82.07	15
COM2.2.6.6	COM	COM2.2	Ktchenette	TP_{25}	2020-11-06	Nov	Fall	48020	8:29	25	4.10	102.59	15
COM2.2.7.6	COM	COM2.2	Ktchenette	TP_{30}	2020-11-06	Nov	Fall	48020	8:34	30	4.10	123.10	2000
COM3.1.1.6	COM	COM3.1	Laboratory	TP_0	2020-11-06	Nov	Fall	37210	6:54	0	0.00	0.00	2000
COM3.1.2.6	COM	COM3.1	Laboratory	TP_5	2020-11-06	Nov	Fall	37210	6:59	5	4.80	23.98	15

COM3.1.3.6	COM	COM3.1	Laboratory	TP_{10}	2020-11-06	Nov	Fall	37210	7:04	10	4.80	47.95	15
COM3.1.4.6	COM	COM3.1	Laboratory	TP_{15}	2020-11-06	Nov	Fall	37210	7:09	15	4.80	71.93	15
COM3.1.5.6	COM	COM3.1	Laboratory	TP_{20}	2020-11-06	Nov	Fall	37210	7:14	20	4.80	95.91	15
COM3.1.6.6	COM	COM3.1	Laboratory	TP_{25}	2020-11-06	Nov	Fall	37210	7:19	25	4.80	119.88	15
COM3.1.7.6	COM	COM3.1	Laboratory	TP_{30}	2020-11-06	Nov	Fall	37210	7:24	30	4.80	143.86	2000
COM3.2.1.6	COM	COM3.2	Ktchenette	TP_{0}	2020-11-06	Nov	Fall	37210	7:52	0	0.00	0.00	2000
COM3.2.2.6	COM	COM3.2	Ktchenette	TP_{5}	2020-11-06	Nov	Fall	37210	7:57	5	5.34	26.70	15
COM3.2.3.6	COM	COM3.2	Ktchenette	TP_{10}	2020-11-06	Nov	Fall	37210	8:02	10	5.34	53.41	15
COM3.2.4.6	COM	COM3.2	Ktchenette	TP_{15}	2020-11-06	Nov	Fall	37210	8:07	15	5.34	80.11	15
COM3.2.5.6	COM	COM3.2	Ktchenette	TP_{20}	2020-11-06	Nov	Fall	37210	8:12	20	5.34	106.82	15
COM3.2.6.6	COM	COM3.2	Ktchenette	TP_{25}	2020-11-06	Nov	Fall	37210	8:17	25	5.34	133.52	15
COM3.2.7.6	COM	COM3.2	Ktchenette	TP_{30}	2020-11-06	Nov	Fall	37210	8:22	30	5.34	160.23	2000
RES1.1.6	RES	RES1	Kitchen	TP_{0}	2020-11-05	Nov	Fall	757	6:43	0	0.00	0.00	2000
RES1.2.6	RES	RES1	Kitchen	TP_{5}	2020-11-05	Nov	Fall	757	6:48	5	4.04	20.20	15
RES1.3.6	RES	RES1	Kitchen	TP_{10}	2020-11-05	Nov	Fall	757	6:53	10	4.04	40.41	15
RES1.4.6	RES	RES1	Kitchen	TP_{15}	2020-11-05	Nov	Fall	757	6:58	15	4.04	60.61	15
RES1.5.6	RES	RES1	Kitchen	TP_{20}	2020-11-05	Nov	Fall	757	7:03	20	4.04	80.82	15
RES1.6.6	RES	RES1	Kitchen	TP_{25}	2020-11-05	Nov	Fall	757	7:08	25	4.04	101.02	15
RES1.7.6	RES	RES1	Kitchen	TP_{30}	2020-11-05	Nov	Fall	757	7:13	30	4.04	121.23	2000
RES2.1.6	RES	RES2	Bathroom	TP_{0}	2020-11-05	Nov	Fall	2230	8:29	0	0.00	0.00	2000
RES2.2.6	RES	RES2	Bathroom	TP_{5}	2020-11-05	Nov	Fall	2230	8:34	5	8.14	40.71	15
RES2.3.6	RES	RES2	Bathroom	TP_{10}	2020-11-05	Nov	Fall	2230	8:39	10	8.14	81.43	15
RES2.4.6	RES	RES2	Bathroom	TP_{15}	2020-11-05	Nov	Fall	2230	8:44	15	8.14	122.14	15
RES2.5.6	RES	RES2	Bathroom	TP_{20}	2020-11-05	Nov	Fall	2230	8:49	20	8.14	162.86	15
RES2.6.6	RES	RES2	Bathroom	TP_{25}	2020-11-05	Nov	Fall	2230	8:54	25	8.14	203.57	15
RES2.7.6	RES	RES2	Bathroom	TP_{30}	2020-11-05	Nov	Fall	2230	8:59	30	8.14	244.29	2000
RES3.1.6	RES	RES3	Bathroom	TP_{0}	2020-11-05	Nov	Fall	1499	7:05	0	0.00	0.00	2000
RES3.2.6	RES	RES3	Bathroom	TP_{5}	2020-11-05	Nov	Fall	1499	7:10	5	2.96	14.82	15
RES3.3.6	RES	RES3	Bathroom	TP_{10}	2020-11-05	Nov	Fall	1499	7:15	10	2.96	29.64	15
RES3.4.6	RES	RES3	Bathroom	TP_{15}	2020-11-05	Nov	Fall	1499	7:20	15	2.96	44.47	15
RES3.5.6	RES	RES3	Bathroom	TP_{20}	2020-11-05	Nov	Fall	1499	7:25	20	2.96	59.29	15

RES3.6.6	RES	RES3	Bathroom	TP_{25}	2020-11-05	Nov	Fall	1499	7:30	25	2.96	74.11	15
RES3.7.6	RES	RES3	Bathroom	TP_{30}	2020-11-05	Nov	Fall	1499	7:35	30	2.96	88.93	2000
RES4.1.6	RES	RES4	Kitchen	TP_{0}	2020-11-05	Nov	Fall	1890	8:07	0	0.00	0.00	2000
RES4.2.6	RES	RES4	Kitchen	TP_{5}	2020-11-05	Nov	Fall	1890	8:12	5	2.08	10.39	15
RES4.3.6	RES	RES4	Kitchen	TP_{10}	2020-11-05	Nov	Fall	1890	8:17	10	2.08	20.79	15
RES4.4.6	RES	RES4	Kitchen	TP_{15}	2020-11-05	Nov	Fall	1890	8:22	15	2.08	31.18	15
RES4.5.6	RES	RES4	Kitchen	TP_{20}	2020-11-05	Nov	Fall	1890	8:27	20	2.08	41.58	15
RES4.6.6	RES	RES4	Kitchen	TP_{25}	2020-11-05	Nov	Fall	1890	8:32	25	2.08	51.97	15
RES4.7.6	RES	RES4	Kitchen	TP_{30}	2020-11-05	Nov	Fall	1890	8:37	30	2.08	62.37	2000
COM1.1.1.5	COM	COM1.1	Bathroom	TP_{0}	2020-10-01	Oct	Fall	65850	8:54	0	0.00	0.00	2000
COM1.1.2.5	COM	COM1.1	Bathroom	TP_{5}	2020-10-01	Oct	Fall	65850	8:59	5	7.39	36.95	15
COM1.1.3.5	COM	COM1.1	Bathroom	TP_{10}	2020-10-01	Oct	Fall	65850	9:04	10	7.39	73.90	15
COM1.1.4.5	COM	COM1.1	Bathroom	TP_{15}	2020-10-01	Oct	Fall	65850	9:09	15	7.39	110.85	15
COM1.1.5.5	COM	COM1.1	Bathroom	TP_{20}	2020-10-01	Oct	Fall	65850	9:14	20	7.39	147.79	15
COM1.1.6.5	COM	COM1.1	Bathroom	TP_{25}	2020-10-01	Oct	Fall	65850	9:19	25	7.39	184.74	15
COM1.1.7.5	COM	COM1.1	Bathroom	TP_{30}	2020-10-01	Oct	Fall	65850	9:24	30	7.39	221.69	2000
COM1.2.1.5	COM	COM1.2	Laboratory	TP_{0}	2020-10-01	Oct	Fall	65850	9:28	0	0.00	0.00	2000
COM1.2.2.5	COM	COM1.2	Laboratory	TP_{5}	2020-10-01	Oct	Fall	65850	9:33	5	3.00	15.00	15
COM1.2.3.5	COM	COM1.2	Laboratory	TP_{10}	2020-10-01	Oct	Fall	65850	9:38	10	3.00	30.00	15
COM1.2.4.5	COM	COM1.2	Laboratory	TP_{15}	2020-10-01	Oct	Fall	65850	9:43	15	3.00	45.00	15
COM1.2.5.5	COM	COM1.2	Laboratory	TP_{20}	2020-10-01	Oct	Fall	65850	9:48	20	3.00	60.00	15
COM1.2.6.5	COM	COM1.2	Laboratory	TP_{25}	2020-10-01	Oct	Fall	65850	9:53	25	3.00	75.00	15
COM1.2.7.5	COM	COM1.2	Laboratory	TP_{30}	2020-10-01	Oct	Fall	65850	9:58	30	3.00	90.00	2000
COM2.1.1.5	COM	COM2.1	Laboratory	TP_{0}	2020-10-02	Oct	Fall	45240	5:25	0	0.00	0.00	2000
COM2.1.2.5	COM	COM2.1	Laboratory	TP_{5}	2020-10-02	Oct	Fall	45240	5:30	5	5.54	27.68	15
COM2.1.3.5	COM	COM2.1	Laboratory	TP_{10}	2020-10-02	Oct	Fall	45240	5:35	10	5.54	55.36	15
COM2.1.4.5	COM	COM2.1	Laboratory	TP_{15}	2020-10-02	Oct	Fall	45240	5:40	15	5.54	83.04	15
COM2.1.5.5	COM	COM2.1	Laboratory	TP_{20}	2020-10-02	Oct	Fall	45240	5:45	20	5.54	110.71	15
COM2.1.6.5	COM	COM2.1	Laboratory	TP_{25}	2020-10-02	Oct	Fall	45240	5:50	25	5.54	138.39	15
COM2.1.7.5	COM	COM2.1	Laboratory	TP_{30}	2020-10-02	Oct	Fall	45240	5:55	30	5.54	166.07	2000
COM2.2.1.5	COM	COM2.2	Ktchenette	TP_{0}	2020-10-02	Oct	Fall	45240	6:17	0	0.00	0.00	2000

COM2.2.2.5	COM	COM2.2	Ktchenette	TP_5	2020-10-02	Oct	Fall	45240	6:22	5	2.64	13.19	15
COM2.2.3.5	COM	COM2.2	Ktchenette	TP_{10}	2020-10-02	Oct	Fall	45240	6:27	10	2.64	26.38	15
COM2.2.4.5	COM	COM2.2	Ktchenette	TP_{15}	2020-10-02	Oct	Fall	45240	6:32	15	2.64	39.57	15
COM2.2.5.5	COM	COM2.2	Ktchenette	TP_{20}	2020-10-02	Oct	Fall	45240	6:37	20	2.64	52.77	15
COM2.2.6.5	COM	COM2.2	Ktchenette	TP_{25}	2020-10-02	Oct	Fall	45240	6:42	25	2.64	65.96	15
COM2.2.7.5	COM	COM2.2	Ktchenette	TP_{30}	2020-10-02	Oct	Fall	45240	6:47	30	2.64	79.15	2000
COM3.1.1.5	COM	COM3.1	Laboratory	TP_0	2020-10-02	Oct	Fall	35910	8:23	0	0.00	0.00	2000
COM3.1.2.5	COM	COM3.1	Laboratory	TP_5	2020-10-02	Oct	Fall	35910	8:28	5	7.39	36.95	15
COM3.1.3.5	COM	COM3.1	Laboratory	TP_{10}	2020-10-02	Oct	Fall	35910	8:33	10	7.39	73.90	15
COM3.1.4.5	COM	COM3.1	Laboratory	TP_{15}	2020-10-02	Oct	Fall	35910	8:38	15	7.39	110.85	15
COM3.1.5.5	COM	COM3.1	Laboratory	TP_{20}	2020-10-02	Oct	Fall	35910	8:43	20	7.39	147.79	15
COM3.1.6.5	COM	COM3.1	Laboratory	TP_{25}	2020-10-02	Oct	Fall	35910	8:48	25	7.39	184.74	15
COM3.1.7.5	COM	COM3.1	Laboratory	TP_{30}	2020-10-02	Oct	Fall	35910	8:53	30	7.39	221.69	2000
COM3.2.1.5	COM	COM3.2	Ktchenette	TP_0	2020-10-02	Oct	Fall	35910	7:22	0	0.00	0.00	2000
COM3.2.2.5	COM	COM3.2	Ktchenette	TP_5	2020-10-02	Oct	Fall	35910	7:27	5	5.29	26.45	15
COM3.2.3.5	COM	COM3.2	Ktchenette	TP_{10}	2020-10-02	Oct	Fall	35910	7:32	10	5.29	52.91	15
COM3.2.4.5	COM	COM3.2	Ktchenette	TP_{15}	2020-10-02	Oct	Fall	35910	7:37	15	5.29	79.36	15
COM3.2.5.5	COM	COM3.2	Ktchenette	TP_{20}	2020-10-02	Oct	Fall	35910	7:42	20	5.29	105.82	15
COM3.2.6.5	COM	COM3.2	Ktchenette	TP_{25}	2020-10-02	Oct	Fall	35910	7:47	25	5.29	132.27	15
COM3.2.7.5	COM	COM3.2	Ktchenette	TP_{30}	2020-10-02	Oct	Fall	35910	7:52	30	5.29	158.73	2000
RES1.1.5	RES	RES1	Kitchen	TP_0	2020-10-01	Oct	Fall	748	8:49	0	0.00	0.00	2000
RES1.2.5	RES	RES1	Kitchen	TP_5	2020-10-01	Oct	Fall	748	8:54	5	7.67	38.33	15
RES1.3.5	RES	RES1	Kitchen	TP_{10}	2020-10-01	Oct	Fall	748	8:59	10	7.67	76.67	15
RES1.4.5	RES	RES1	Kitchen	TP_{15}	2020-10-01	Oct	Fall	748	9:04	15	7.67	115.00	15
RES1.5.5	RES	RES1	Kitchen	TP_{20}	2020-10-01	Oct	Fall	748	9:09	20	7.67	153.33	15
RES1.6.5	RES	RES1	Kitchen	TP_{25}	2020-10-01	Oct	Fall	748	9:14	25	7.67	191.67	15
RES1.7.5	RES	RES1	Kitchen	TP_{30}	2020-10-01	Oct	Fall	748	9:19	30	7.67	230.00	2000
RES2.1.5	RES	RES2	Bathroom	TP_0	2020-10-01	Oct	Fall	2253	8:45	0	0.00	0.00	2000
RES2.2.5	RES	RES2	Bathroom	TP_5	2020-10-01	Oct	Fall	2253	8:50	5	3.43	17.14	15
RES2.3.5	RES	RES2	Bathroom	TP_{10}	2020-10-01	Oct	Fall	2253	8:55	10	3.43	34.29	15
RES2.4.5	RES	RES2	Bathroom	TP_{15}	2020-10-01	Oct	Fall	2253	9:00	15	3.43	51.43	15

RES2.5.5	RES	RES2	Bathroom	TP_{20}	2020-10-01	Oct	Fall	2253	9:05	20	3.43	68.57	15
RES2.6.5	RES	RES2	Bathroom	TP_{25}	2020-10-01	Oct	Fall	2253	9:10	25	3.43	85.71	15
RES2.7.5	RES	RES2	Bathroom	TP_{30}	2020-10-01	Oct	Fall	2253	9:15	30	3.43	102.86	2000
RES3.1.5	RES	RES3	Bathroom	TP_{0}	2020-10-01	Oct	Fall	1402	6:01	0	0.00	0.00	2000
RES3.2.5	RES	RES3	Bathroom	TP_{5}	2020-10-01	Oct	Fall	1402	6:06	5	5.71	28.57	15
RES3.3.5	RES	RES3	Bathroom	TP_{10}	2020-10-01	Oct	Fall	1402	6:11	10	5.71	57.14	15
RES3.4.5	RES	RES3	Bathroom	TP_{15}	2020-10-01	Oct	Fall	1402	6:16	15	5.71	85.71	15
RES3.5.5	RES	RES3	Bathroom	TP_{20}	2020-10-01	Oct	Fall	1402	6:21	20	5.71	114.29	15
RES3.6.5	RES	RES3	Bathroom	TP_{25}	2020-10-01	Oct	Fall	1402	6:26	25	5.71	142.86	15
RES3.7.5	RES	RES3	Bathroom	TP_{30}	2020-10-01	Oct	Fall	1402	6:31	30	5.71	171.43	2000
RES4.1.5	RES	RES4	Kitchen	TP_{0}	2020-10-01	Oct	Fall	1580	9:24	0	0.00	0.00	2000
RES4.2.5	RES	RES4	Kitchen	TP_{5}	2020-10-01	Oct	Fall	1580	9:29	5	2.06	10.31	15
RES4.3.5	RES	RES4	Kitchen	TP_{10}	2020-10-01	Oct	Fall	1580	9:34	10	2.06	20.61	15
RES4.4.5	RES	RES4	Kitchen	TP_{15}	2020-10-01	Oct	Fall	1580	9:39	15	2.06	30.92	15
RES4.5.5	RES	RES4	Kitchen	TP_{20}	2020-10-01	Oct	Fall	1580	9:44	20	2.06	41.22	15
RES4.6.5	RES	RES4	Kitchen	TP_{25}	2020-10-01	Oct	Fall	1580	9:49	25	2.06	51.53	15
RES4.7.5	RES	RES4	Kitchen	TP_{30}	2020-10-01	Oct	Fall	1580	9:54	30	2.06	61.83	2000
COM1.1.1.4	COM	COM1.1	Bathroom	TP_{0}	2020-08-06	Sep	Fall	76770	9:03	0	0.00	0.00	2000
COM1.1.2.4	COM	COM1.1	Bathroom	TP_{5}	2020-09-03	Sep	Fall	76770	9:08	5	4.49	22.44	15
COM1.1.3.4	COM	COM1.1	Bathroom	TP_{10}	2020-09-03	Sep	Fall	76770	9:13	10	4.49	44.87	15
COM1.1.4.4	COM	COM1.1	Bathroom	TP_{15}	2020-09-03	Sep	Fall	76770	9:18	15	4.49	67.31	15
COM1.1.5.4	COM	COM1.1	Bathroom	TP_{20}	2020-09-03	Sep	Fall	76770	9:23	20	4.49	89.74	15
COM1.1.6.4	COM	COM1.1	Bathroom	TP_{25}	2020-09-03	Sep	Fall	76770	9:28	25	4.49	112.18	15
COM1.1.7.4	COM	COM1.1	Bathroom	TP_{30}	2020-09-03	Sep	Fall	76770	9:33	30	4.49	134.62	2000
COM1.2.1.4	COM	COM1.2	Laboratory	TP_{0}	2020-09-03	Sep	Fall	76770	8:11	0	0.00	0.00	2000
COM1.2.2.4	COM	COM1.2	Laboratory	TP_{5}	2020-09-03	Sep	Fall	76770	8:16	5	6.08	30.39	15
COM1.2.3.4	COM	COM1.2	Laboratory	TP_{10}	2020-09-03	Sep	Fall	76770	8:21	10	6.08	60.79	15
COM1.2.4.4	COM	COM1.2	Laboratory	TP_{15}	2020-09-03	Sep	Fall	76770	8:26	15	6.08	91.18	15
COM1.2.5.4	COM	COM1.2	Laboratory	TP_{20}	2020-09-03	Sep	Fall	76770	8:31	20	6.08	121.58	15
COM1.2.6.4	COM	COM1.2	Laboratory	TP_{25}	2020-09-03	Sep	Fall	76770	8:36	25	6.08	151.97	15
COM1.2.7.4	COM	COM1.2	Laboratory	TP_{30}	2020-09-03	Sep	Fall	76770	8:41	30	6.08	182.37	2000

COM2.1.1.4	COM	COM2.1	Laboratory	TP_0	2020-09-04	Sep	Fall	39620	6:57	0	0.00	0.00	2000
COM2.1.2.4	COM	COM2.1	Laboratory	TP_5	2020-09-04	Sep	Fall	39620	7:02	5	2.00	10.00	15
COM2.1.3.4	COM	COM2.1	Laboratory	TP_{10}	2020-09-04	Sep	Fall	39620	7:07	10	2.00	20.00	15
COM2.1.4.4	COM	COM2.1	Laboratory	TP_{15}	2020-09-04	Sep	Fall	39620	7:12	15	2.00	30.00	15
COM2.1.5.4	COM	COM2.1	Laboratory	TP_{20}	2020-09-04	Sep	Fall	39620	7:17	20	2.00	40.00	15
COM2.1.6.4	COM	COM2.1	Laboratory	TP_{25}	2020-09-04	Sep	Fall	39620	7:22	25	2.00	50.00	15
COM2.1.7.4	COM	COM2.1	Laboratory	TP_{30}	2020-09-04	Sep	Fall	39620	7:27	30	2.00	60.00	2000
COM2.2.1.4	COM	COM2.2	Ktchenette	TP_0	2020-09-04	Sep	Fall	39620	7:47	0	0.00	0.00	2000
COM2.2.2.4	COM	COM2.2	Ktchenette	TP_5	2020-09-04	Sep	Fall	39620	7:52	5	3.00	15.00	15
COM2.2.3.4	COM	COM2.2	Ktchenette	TP_{10}	2020-09-04	Sep	Fall	39620	7:57	10	3.00	30.00	15
COM2.2.4.4	COM	COM2.2	Ktchenette	TP_{15}	2020-09-04	Sep	Fall	39620	8:02	15	3.00	45.00	15
COM2.2.5.4	COM	COM2.2	Ktchenette	TP_{20}	2020-09-04	Sep	Fall	39620	8:07	20	3.00	60.00	15
COM2.2.6.4	COM	COM2.2	Ktchenette	TP_{25}	2020-09-04	Sep	Fall	39620	8:12	25	3.00	75.00	15
COM2.2.7.4	COM	COM2.2	Ktchenette	TP_{30}	2020-09-04	Sep	Fall	39620	8:17	30	3.00	90.00	2000
COM3.1.1.4	COM	COM3.1	Laboratory	TP_0	2020-09-04	Sep	Fall	83840	5:11	0	0.00	0.00	2000
COM3.1.2.4	COM	COM3.1	Laboratory	TP_5	2020-09-04	Sep	Fall	83840	5:16	5	3.33	16.67	15
COM3.1.3.4	COM	COM3.1	Laboratory	TP_{10}	2020-09-04	Sep	Fall	83840	5:21	10	3.33	33.33	15
COM3.1.4.4	COM	COM3.1	Laboratory	TP_{15}	2020-09-04	Sep	Fall	83840	5:26	15	3.33	50.00	15
COM3.1.5.4	COM	COM3.1	Laboratory	TP_{20}	2020-09-04	Sep	Fall	83840	5:31	20	3.33	66.67	15
COM3.1.6.4	COM	COM3.1	Laboratory	TP_{25}	2020-09-04	Sep	Fall	83840	5:36	25	3.33	83.33	15
COM3.1.7.4	COM	COM3.1	Laboratory	TP_{30}	2020-09-04	Sep	Fall	83840	5:41	30	3.33	100.00	2000
COM3.2.1.4	COM	COM3.2	Ktchenette	TP_0	2020-09-04	Sep	Fall	83840	7:43	0	0.00	0.00	2000
COM3.2.2.4	COM	COM3.2	Ktchenette	TP_5	2020-09-04	Sep	Fall	83840	7:48	5	4.71	23.56	15
COM3.2.3.4	COM	COM3.2	Ktchenette	TP_{10}	2020-09-04	Sep	Fall	83840	7:53	10	4.71	47.11	15
COM3.2.4.4	COM	COM3.2	Ktchenette	TP_{15}	2020-09-04	Sep	Fall	83840	7:58	15	4.71	70.67	15
COM3.2.5.4	COM	COM3.2	Ktchenette	TP_{20}	2020-09-04	Sep	Fall	83840	8:03	20	4.71	94.22	15
COM3.2.6.4	COM	COM3.2	Ktchenette	TP_{25}	2020-09-04	Sep	Fall	83840	8:08	25	4.71	117.78	15
COM3.2.7.4	COM	COM3.2	Ktchenette	TP_{30}	2020-09-04	Sep	Fall	83840	8:13	30	4.71	141.33	2000
RES1.1.4	RES	RES1	Kitchen	TP_0	2020-09-03	Sep	Fall	885	6:01	0	0.00	0.00	2000
RES1.2.4	RES	RES1	Kitchen	TP_5	2020-09-03	Sep	Fall	885	6:06	5	3.08	15.38	15
RES1.3.4	RES	RES1	Kitchen	TP_{10}	2020-09-03	Sep	Fall	885	6:11	10	3.08	30.77	15

RES1.4.4	RES	RES1	Kitchen	TP_{15}	2020-09-03	Sep	Fall	885	6:16	15	3.08	46.15	15
RES1.5.4	RES	RES1	Kitchen	TP_{20}	2020-09-03	Sep	Fall	885	6:21	20	3.08	61.54	15
RES1.6.4	RES	RES1	Kitchen	TP_{25}	2020-09-03	Sep	Fall	885	6:26	25	3.08	76.92	15
RES1.7.4	RES	RES1	Kitchen	TP_{30}	2020-09-03	Sep	Fall	885	6:31	30	3.08	92.31	2000
RES2.1.4	RES	RES2	Bathroom	TP_{0}	2020-09-03	Sep	Fall	1853	6:19	0	0.00	0.00	2000
RES2.2.4	RES	RES2	Bathroom	TP_{5}	2020-09-03	Sep	Fall	1853	6:24	5	2.42	12.12	15
RES2.3.4	RES	RES2	Bathroom	TP_{10}	2020-09-03	Sep	Fall	1853	6:29	10	2.42	24.25	15
RES2.4.4	RES	RES2	Bathroom	TP_{15}	2020-09-03	Sep	Fall	1853	6:34	15	2.42	36.37	15
RES2.5.4	RES	RES2	Bathroom	TP_{20}	2020-09-03	Sep	Fall	1853	6:39	20	2.42	48.50	15
RES2.6.4	RES	RES2	Bathroom	TP_{25}	2020-09-03	Sep	Fall	1853	6:44	25	2.42	60.62	15
RES2.7.4	RES	RES2	Bathroom	TP_{30}	2020-09-03	Sep	Fall	1853	6:49	30	2.42	72.75	2000
RES3.1.4	RES	RES3	Bathroom	TP_{0}	2020-09-03	Sep	Fall	1379	6:00	0	0.00	0.00	2000
RES3.2.4	RES	RES3	Bathroom	TP_{5}	2020-09-03	Sep	Fall	1379	6:05	5	2.08	10.40	15
RES3.3.4	RES	RES3	Bathroom	TP_{10}	2020-09-03	Sep	Fall	1379	6:10	10	2.08	20.81	15
RES3.4.4	RES	RES3	Bathroom	TP_{15}	2020-09-03	Sep	Fall	1379	6:15	15	2.08	31.21	15
RES3.5.4	RES	RES3	Bathroom	TP_{20}	2020-09-03	Sep	Fall	1379	6:20	20	2.08	41.62	15
RES3.6.4	RES	RES3	Bathroom	TP_{25}	2020-09-03	Sep	Fall	1379	6:25	25	2.08	52.02	15
RES3.7.4	RES	RES3	Bathroom	TP_{30}	2020-09-03	Sep	Fall	1379	6:30	30	2.08	62.43	2000
RES4.1.4	RES	RES4	Kitchen	TP_{0}	2020-09-03	Sep	Fall	1530	6:38	0	0.00	0.00	2000
RES4.2.4	RES	RES4	Kitchen	TP_{5}	2020-09-03	Sep	Fall	1530	6:43	5	2.14	10.69	15
RES4.3.4	RES	RES4	Kitchen	TP_{10}	2020-09-03	Sep	Fall	1530	6:48	10	2.14	21.38	15
RES4.4.4	RES	RES4	Kitchen	TP_{15}	2020-09-03	Sep	Fall	1530	6:53	15	2.14	32.07	15
RES4.5.4	RES	RES4	Kitchen	TP_{20}	2020-09-03	Sep	Fall	1530	6:58	20	2.14	42.77	15
RES4.6.4	RES	RES4	Kitchen	TP_{25}	2020-09-03	Sep	Fall	1530	7:03	25	2.14	53.46	15
RES4.7.4	RES	RES4	Kitchen	TP_{30}	2020-09-03	Sep	Fall	1530	7:08	30	2.14	64.15	2000

Abbreviation: COM, commercial building; RES, residential household; Jun, June; Jul, July; Aug, August; Sep, September; Oct, October; Nov, November.
*Naming convention encompass (i) site type, (ii) site, (iii) time point, and (iv) month. See Example 1 and Example 2 below.
Example 1: COM3.2.1.6 (i) Commercial building (ii) site 3.2, (iii) time point 1, (iv) month 6
Example 2: RES4.7.6 (i) Residential household (ii) site 4, (iii) time point 7, (iv) month 6

Table S1: Continue.

Sample Id*	Total cell counts (TCC, cells.ml^{-1})	Intact cell counts (ICC, cells.ml^{-1})	High nucleic acid bacteria (HNA, cells.ml^{-1})	Phenotypic diversity index (D2)	Evenness
COM1.1.1.3	1.05E+05	2.08E+04	5.42E+04	2232	0.23
COM1.1.2.3	9.51E+04	1.21E+04	4.51E+04	2294	0.23
COM1.1.3.3	7.27E+04	6.29E+03	3.39E+04	2132	0.22
COM1.1.4.3	6.21E+04	3.67E+03	2.70E+04	2103	0.22
COM1.1.5.3	8.51E+04	4.45E+03	3.60E+04	2025	0.21
COM1.1.6.3	9.53E+04	5.14E+03	4.11E+04	2046	0.21
COM1.1.7.3	7.42E+04	3.69E+03	2.71E+04	2087	0.21
COM1.2.1.3	1.03E+06	3.62E+05	3.62E+05	1459	0.19
COM1.2.2.3	6.27E+04	4.37E+03	3.41E+04	2301	0.23
COM1.2.3.3	4.16E+04	3.25E+03	2.24E+04	2480	0.25
COM1.2.4.3	3.54E+04	2.42E+03	1.88E+04	2598	0.24
COM1.2.5.3	2.81E+04	2.24E+03	1.41E+04	2778	0.26
COM1.2.6.3	3.36E+04	3.10E+03	1.71E+04	2626	0.24
COM1.2.7.3	3.34E+04	3.00E+03	1.73E+04	2571	0.25
COM2.1.1.3	6.76E+05	3.72E+05	2.95E+05	1399	0.18
COM2.1.2.3	3.68E+04	5.67E+03	1.58E+04	2724	0.25
COM2.1.3.3	3.13E+04	4.82E+03	1.33E+04	2795	0.25
COM2.1.4.3	3.05E+04	4.82E+03	1.36E+04	2840	0.25
COM2.1.5.3	3.21E+04	5.11E+03	1.39E+04	2805	0.26
COM2.1.6.3	3.29E+04	5.21E+03	1.44E+04	2719	0.25
COM2.1.7.3	3.12E+04	5.26E+03	1.30E+04	2864	0.25
COM2.2.1.3	3.25E+04	6.13E+03	1.84E+04	2601	0.25
COM2.2.2.3	2.68E+04	2.85E+03	1.20E+04	2967	0.26
COM2.2.3.3	2.81E+04	2.45E+03	1.25E+04	2937	0.25
COM2.2.4.3	2.81E+04	2.29E+03	1.16E+04	3050	0.25

COM2.2.5.3	2.62E+04	1.95E+03	1.09E+04	3017	0.26
COM2.2.6.3	2.51E+04	1.69E+03	1.02E+04	2913	0.26
COM2.2.7.3	2.57E+04	1.22E+03	1.12E+04	2952	0.25
COM3.1.1.3	2.37E+06	9.54E+05	9.76E+05	1416	0.20
COM3.1.2.3	2.45E+05	1.17E+05	1.43E+05	2148	0.24
COM3.1.3.3	1.05E+05	3.65E+04	5.10E+04	2373	0.24
COM3.1.4.3	5.03E+04	1.58E+04	2.20E+04	2473	0.24
COM3.1.5.3	4.61E+04	1.36E+04	2.11E+04	2562	0.24
COM3.1.6.3	4.24E+04	1.29E+04	1.98E+04	2503	0.25
COM3.1.7.3	4.07E+04	9.38E+03	2.38E+04	2653	0.25
COM3.2.1.3	2.10E+04	5.12E+02	7.89E+03	2816	0.25
COM3.2.2.3	2.28E+04	7.99E+02	8.24E+03	2845	0.26
COM3.2.3.3	1.92E+04	7.26E+02	6.60E+03	2723	0.27
COM3.2.4.3	1.78E+04	6.86E+02	6.33E+03	2815	0.26
COM3.2.5.3	2.13E+04	5.13E+02	7.51E+03	2817	0.25
COM3.2.6.3	2.23E+04	5.79E+02	8.06E+03	2686	0.25
COM3.2.7.3	2.29E+04	7.26E+02	7.92E+03	2812	0.25
RES1.1.3	1.52E+04	4.13E+02	7.20E+03	2713	0.28
RES1.2.3	2.14E+04	1.17E+03	1.11E+04	2723	0.26
RES1.3.3	1.99E+04	1.16E+03	1.01E+04	2659	0.26
RES1.4.3	1.99E+04	1.03E+03	1.02E+04	2866	0.26
RES1.5.3	2.08E+04	9.33E+02	1.08E+04	2613	0.27
RES1.6.3	2.09E+04	1.04E+03	1.05E+04	2707	0.27
RES1.7.3	1.81E+04	5.66E+02	8.81E+03	2752	0.27
RES2.1.3	1.60E+04	2.67E+02	5.41E+03	2806	0.27
RES2.2.3	1.80E+04	3.59E+02	5.67E+03	2814	0.25
RES2.3.3	1.51E+04	2.40E+02	5.01E+03	2774	0.26
RES2.4.3	1.66E+04	2.46E+02	5.52E+03	2774	0.25

RES2.5.3	1.64E+04	3.06E+02	5.58E+03	2815	0.27
RES2.6.3	1.64E+04	3.66E+02	5.57E+03	2768	0.25
RES2.7.3	1.58E+04	2.46E+02	5.04E+03	2767	0.26
RES3.1.3	2.06E+04	7.67E+02	8.72E+03	2910	0.25
RES3.2.3	1.95E+04	8.32E+02	7.64E+03	2865	0.26
RES3.3.3	1.64E+04	4.52E+02	7.19E+03	2922	0.26
RES3.4.3	1.78E+04	9.65E+02	7.52E+03	2865	0.26
RES3.5.3	1.79E+04	3.52E+02	7.54E+03	2869	0.26
RES3.6.3	1.90E+04	8.06E+02	7.40E+03	2734	0.26
RES3.7.3	1.90E+04	5.26E+02	7.56E+03	2826	0.25
RES4.1.3	1.87E+04	8.53E+02	7.50E+03	2848	0.25
RES4.2.3	2.07E+04	1.18E+03	7.84E+03	2827	0.26
RES4.3.3	2.04E+04	8.32E+02	7.88E+03	2965	0.26
RES4.4.3	1.94E+04	7.19E+02	7.65E+03	2799	0.25
RES4.5.3	1.84E+04	6.19E+02	7.09E+03	2826	0.26
RES4.6.3	2.16E+04	8.19E+02	7.92E+03	2790	0.26
RES4.7.3	1.65E+04	5.79E+02	6.24E+03	2786	0.27
COM1.1.1.2	3.84E+05	1.51E+05	2.15E+05	2097	0.22
COM1.1.2.2	2.24E+05	5.66E+04	1.14E+05	2114	0.22
COM1.1.3.2	2.15E+05	4.67E+04	1.16E+05	1952	0.22
COM1.1.4.2	1.98E+05	2.38E+04	9.39E+04	2053	0.22
COM1.1.5.2	2.07E+05	1.53E+04	9.17E+04	1968	0.21
COM1.1.6.2	1.90E+05	1.36E+04	8.16E+04	1992	0.21
COM1.1.7.2	1.90E+05	1.48E+04	7.14E+04	2002	0.21
COM1.2.1.2	1.01E+06	4.27E+05	6.46E+05	2023	0.23
COM1.2.2.2	1.55E+05	1.61E+04	1.01E+05	1708	0.21
COM1.2.3.2	1.15E+05	1.11E+04	7.33E+04	1830	0.21
COM1.2.4.2	8.81E+04	8.42E+03	5.46E+04	1957	0.22

COM1.2.5.2	5.85E+04	5.34E+03	3.70E+04	2109	0.23
COM1.2.6.2	4.52E+04	4.87E+03	2.84E+04	2220	0.23
COM1.2.7.2	3.78E+04	4.70E+03	2.24E+04	2483	0.24
COM2.1.1.2	3.48E+05	1.87E+05	1.95E+05	1629	0.18
COM2.1.2.2	3.22E+04	6.58E+03	1.67E+04	2728	0.24
COM2.1.3.2	3.23E+04	5.37E+03	1.59E+04	2739	0.24
COM2.1.4.2	5.14E+04	1.42E+04	2.89E+04	2627	0.24
COM2.1.5.2	4.56E+04	1.03E+04	2.51E+04	2663	0.24
COM2.1.6.2	4.51E+04	9.14E+03	2.46E+04	2704	0.23
COM2.1.7.2	3.97E+04	8.61E+03	2.12E+04	2762	0.25
COM2.2.1.2	7.51E+04	2.53E+04	5.21E+04	2313	0.24
COM2.2.2.2	3.03E+04	2.86E+03	1.60E+04	2609	0.25
COM2.2.3.2	2.64E+04	2.77E+03	1.41E+04	2621	0.25
COM2.2.4.2	2.66E+04	2.17E+03	1.41E+04	2700	0.25
COM2.2.5.2	2.63E+04	2.33E+03	1.36E+04	2841	0.25
COM2.2.6.2	2.62E+04	1.87E+03	1.33E+04	2921	0.25
COM2.2.7.2	2.52E+04	2.14E+03	1.30E+04	2816	0.26
COM3.1.1.2	4.99E+04	5.96E+03	2.61E+04	2883	0.25
COM3.1.2.2	2.49E+04	1.47E+03	1.09E+04	3123	0.26
COM3.1.3.2	2.35E+04	1.57E+03	1.06E+04	2981	0.25
COM3.1.4.2	2.34E+04	1.07E+03	9.62E+03	2978	0.26
COM3.1.5.2	2.39E+04	9.26E+02	9.44E+03	2977	0.25
COM3.1.6.2	2.67E+04	8.80E+02	1.11E+04	3105	0.25
COM3.1.7.2	1.91E+04	5.06E+02	7.55E+03	3088	0.27
COM3.2.1.2	1.76E+06	5.24E+05	7.53E+05	1466	0.19
COM3.2.2.2	1.36E+06	7.13E+05	5.72E+05	1558	0.20
COM3.2.3.2	1.07E+06	5.44E+05	5.27E+05	1765	0.21
COM3.2.4.2	3.71E+05	1.92E+05	1.93E+05	1924	0.22

COM3.2.5.2	1.22E+05	4.98E+04	6.25E+04	2153	0.21
COM3.2.6.2	8.89E+04	3.00E+04	4.29E+04	2248	0.22
COM3.2.7.2	6.80E+04	2.13E+04	3.33E+04	2211	0.22
RES1.1.2	1.37E+04	8.12E+02	7.65E+03	2681	0.28
RES1.2.2	1.55E+04	7.66E+02	9.58E+03	2592	0.27
RES1.3.2	1.72E+04	1.12E+03	1.03E+04	2686	0.28
RES1.4.2	1.66E+04	6.79E+02	9.73E+03	2568	0.28
RES1.5.2	1.62E+04	8.40E+02	9.40E+03	2811	0.29
RES1.6.2	1.67E+04	7.32E+02	9.45E+03	2808	0.28
RES1.7.2	1.57E+04	1.01E+03	9.38E+03	2740	0.28
RES2.1.2	1.68E+04	3.47E+02	5.97E+03	3057	0.27
RES2.2.2	1.63E+04	3.67E+02	5.83E+03	2934	0.27
RES2.3.2	1.60E+04	2.53E+02	5.76E+03	3006	0.27
RES2.4.2	1.66E+04	2.46E+02	6.14E+03	3086	0.28
RES2.5.2	1.72E+04	2.07E+02	5.85E+03	2928	0.27
RES2.6.2	2.04E+04	3.07E+02	6.39E+03	2992	0.29
RES2.7.2	1.71E+04	2.33E+02	5.67E+03	2866	0.27
RES3.1.2	2.21E+04	2.26E+03	1.06E+04	2972	0.26
RES3.2.2	1.60E+04	5.13E+02	7.70E+03	2941	0.26
RES3.3.2	1.53E+04	4.26E+02	7.50E+03	2957	0.28
RES3.4.2	1.60E+04	4.33E+02	7.75E+03	2947	0.27
RES3.5.2	1.56E+04	3.66E+02	7.77E+03	3090	0.28
RES3.6.2	1.57E+04	4.32E+02	7.83E+03	2849	0.27
RES3.7.2	1.70E+04	6.32E+02	7.92E+03	3088	0.27
RES4.1.2	2.36E+04	4.25E+03	1.34E+04	3217	0.27
RES4.2.2	2.02E+04	4.80E+02	7.87E+03	2942	0.27
RES4.3.2	1.55E+04	4.67E+02	7.08E+03	3072	0.27
RES4.4.2	1.76E+04	2.80E+02	7.62E+03	3262	0.28

RES4.5.2	1.45E+04	2.60E+02	6.52E+03	2974	0.27
RES4.6.2	1.72E+04	2.47E+02	7.63E+03	3175	0.28
RES4.7.2	1.62E+04	4.86E+02	6.77E+03	3155	0.28
COM1.1.1.1	8.83E+05	5.02E+05	6.07E+05	1975	0.22
COM1.1.2.1	5.74E+05	2.83E+05	3.99E+05	2152	0.24
COM1.1.3.1	3.68E+05	1.76E+05	2.52E+05	2020	0.24
COM1.1.4.1	4.49E+05	2.42E+05	3.12E+05	2033	0.24
COM1.1.5.1	2.31E+05	6.40E+04	1.32E+05	1989	0.24
COM1.1.6.1	3.37E+05	1.26E+05	2.12E+05	1848	0.23
COM1.1.7.1	2.63E+05	9.12E+04	1.50E+05	2303	0.22
COM1.2.1.1	9.50E+05	4.45E+05	5.76E+05	1933	0.20
COM1.2.2.1	1.06E+05	3.54E+04	6.62E+04	2141	0.24
COM1.2.3.1	5.50E+04	3.79E+03	2.17E+04	1698	0.21
COM1.2.4.1	4.51E+04	1.65E+03	1.79E+04	1679	0.22
COM1.2.5.1	3.78E+04	1.24E+03	1.60E+04	1841	0.22
COM1.2.6.1	3.62E+04	1.07E+03	1.57E+04	1847	0.22
COM1.2.7.1	4.30E+04	3.23E+03	2.46E+04	2311	0.23
COM2.1.1.1	9.76E+05	5.22E+05	4.88E+05	1862	0.19
COM2.1.2.1	1.36E+05	7.29E+04	9.66E+04	2214	0.22
COM2.1.3.1	1.46E+05	7.43E+04	1.05E+05	2189	0.21
COM2.1.4.1	1.38E+05	7.41E+04	9.93E+04	2085	0.22
COM2.1.5.1	9.74E+04	4.23E+04	6.55E+04	2207	0.22
COM2.1.6.1	7.34E+04	2.24E+04	4.26E+04	2328	0.24
COM2.1.7.1	6.14E+04	2.11E+04	4.15E+04	2486	0.23
COM2.2.1.1	2.70E+05	1.43E+05	1.92E+05	2199	0.24
COM2.2.2.1	3.83E+04	7.19E+03	2.36E+04	2219	0.25
COM2.2.3.1	3.36E+04	4.44E+03	1.98E+04	2468	0.26
COM2.2.4.1	3.61E+04	4.49E+03	2.14E+04	2436	0.25

COM2.2.5.1	3.77E+04	4.13E+03	2.21E+04	2397	0.25
COM2.2.6.1	3.69E+04	4.10E+03	2.21E+04	2393	0.26
COM2.2.7.1	5.18E+04	1.33E+04	3.39E+04	2812	0.25
COM3.1.1.1	1.34E+06	7.37E+05	6.39E+05	1699	0.20
COM3.1.2.1	1.13E+06	6.05E+05	4.32E+05	1562	0.21
COM3.1.3.1	8.99E+05	4.44E+05	3.31E+05	1500	0.20
COM3.1.4.1	9.60E+05	4.65E+05	3.77E+05	1502	0.20
COM3.1.5.1	6.32E+05	2.47E+05	2.89E+05	1902	0.24
COM3.1.6.1	2.47E+05	3.28E+04	9.42E+04	1699	0.22
COM3.1.7.1	1.66E+05	6.42E+04	8.08E+04	2184	0.22
COM3.2.1.1	4.29E+05	2.25E+05	2.73E+05	1962	0.19
COM3.2.2.1	1.99E+04	1.17E+03	1.15E+04	2539	0.27
COM3.2.3.1	1.41E+04	3.59E+02	7.31E+03	2490	0.28
COM3.2.4.1	1.23E+04	3.99E+02	6.46E+03	2269	0.26
COM3.2.5.1	1.20E+04	2.07E+02	6.49E+03	2296	0.26
COM3.2.6.1	1.19E+04	1.73E+02	6.64E+03	2394	0.27
COM3.2.7.1	1.63E+04	1.08E+03	9.23E+03	3213	0.27
RES1.1.1	1.38E+04	1.61E+03	9.23E+03	2904	0.29
RES1.2.1	1.04E+04	2.93E+02	6.77E+03	2413	0.28
RES1.3.1	1.05E+04	2.47E+02	6.92E+03	2415	0.27
RES1.4.1	1.14E+04	2.33E+02	7.34E+03	2533	0.28
RES1.5.1	1.15E+04	1.40E+02	7.39E+03	2639	0.27
RES1.6.1	8.68E+03	1.60E+02	5.46E+03	2636	0.30
RES1.7.1	1.24E+04	4.60E+02	7.59E+03	3104	0.29
RES2.1.1	8.83E+03	1.47E+02	4.48E+03	3593	0.31
RES2.2.1	8.22E+03	4.70E+01	4.12E+03	3044	0.29
RES2.3.1	8.00E+03	8.00E+01	4.04E+03	3029	0.29
RES2.4.1	7.40E+03	1.00E+02	3.96E+03	2899	0.29

RES2.5.1	7.19E+03	6.70E+01	3.92E+03	2774	0.30
RES2.6.1	8.30E+03	6.00E+01	4.33E+03	2859	0.29
RES2.7.1	1.03E+04	1.07E+02	4.79E+03	3852	0.32
RES3.1.1	3.60E+04	9.77E+03	2.51E+04	2126	0.24
RES3.2.1	1.03E+04	1.20E+02	5.41E+03	2811	0.30
RES3.3.1	8.33E+03	5.30E+01	4.91E+03	2681	0.28
RES3.4.1	8.65E+03	1.13E+02	4.85E+03	2780	0.28
RES3.5.1	9.10E+03	1.07E+02	5.38E+03	2790	0.28
RES3.6.1	8.76E+03	3.30E+01	5.19E+03	2770	0.29
RES3.7.1	1.15E+04	2.07E+02	6.30E+03	3530	0.29
RES4.1.1	2.42E+04	6.71E+03	1.72E+04	3100	0.27
RES4.2.1	8.67E+03	9.30E+01	4.26E+03	2941	0.29
RES4.3.1	8.63E+03	6.00E+01	4.48E+03	3000	0.29
RES4.4.1	7.57E+03	1.13E+02	4.13E+03	2784	0.30
RES4.5.1	9.18E+03	9.30E+01	4.74E+03	2993	0.29
RES4.6.1	8.40E+03	1.00E+02	4.68E+03	2733	0.28
RES4.7.1	1.31E+04	1.87E+02	6.59E+03	3598	0.28
COM1.1.1.6	1.08E+06	4.10E+05	6.99E+05	1711	0.21
COM1.1.2.6	2.85E+04	3.32E+03	1.43E+04	2126	0.23
COM1.1.3.6	2.06E+04	2.15E+03	1.09E+04	2566	0.26
COM1.1.4.6	1.98E+04	2.08E+03	1.12E+04	2529	0.25
COM1.1.5.6	1.93E+04	2.50E+03	1.11E+04	2679	0.26
COM1.1.6.6	1.75E+04	1.78E+03	9.41E+03	2588	0.27
COM1.1.7.6	1.81E+04	1.99E+03	1.06E+04	2679	0.27
COM1.2.1.6	2.92E+04	1.49E+03	1.44E+04	2455	0.24
COM1.2.2.6	2.24E+04	1.32E+03	1.06E+04	2520	0.25
COM1.2.3.6	2.53E+04	1.38E+03	1.17E+04	2470	0.24
COM1.2.4.6	2.55E+04	1.13E+03	1.12E+04	2338	0.24

COM1.2.5.6	2.55E+04	7.92E+02	1.13E+04	2564	0.25
COM1.2.6.6	2.21E+04	7.59E+02	1.03E+04	2653	0.26
COM1.2.7.6	2.56E+04	6.39E+02	1.31E+04	2756	0.26
COM2.1.1.6	7.06E+04	1.94E+04	3.01E+04	2512	0.23
COM2.1.2.6	5.71E+04	1.28E+04	2.52E+04	2368	0.22
COM2.1.3.6	4.51E+04	9.38E+03	2.01E+04	2375	0.23
COM2.1.4.6	4.61E+04	8.54E+03	1.97E+04	2380	0.24
COM2.1.5.6	4.87E+04	1.01E+04	2.14E+04	2398	0.23
COM2.1.6.6	3.19E+04	6.10E+03	1.45E+04	2434	0.24
COM2.1.7.6	2.38E+04	4.73E+03	1.00E+04	2341	0.25
COM2.2.1.6	1.05E+04	2.09E+03	5.87E+03	2586	0.28
COM2.2.2.6	7.39E+03	7.20E+02	3.59E+03	3198	0.30
COM2.2.3.6	6.73E+03	6.99E+02	3.36E+03	3155	0.31
COM2.2.4.6	7.04E+03	5.72E+02	3.70E+03	3008	0.30
COM2.2.5.6	7.42E+03	6.79E+02	3.74E+03	3063	0.29
COM2.2.6.6	7.44E+03	5.06E+02	3.60E+03	3192	0.30
COM2.2.7.6	6.80E+03	5.32E+02	3.24E+03	3266	0.31
COM3.1.1.6	4.79E+05	1.69E+05	1.69E+05	1483	0.18
COM3.1.2.6	1.84E+04	1.84E+03	6.24E+03	2502	0.27
COM3.1.3.6	1.68E+04	1.43E+03	6.01E+03	2549	0.26
COM3.1.4.6	1.11E+04	7.79E+02	4.53E+03	2687	0.28
COM3.1.5.6	1.01E+04	7.79E+02	4.32E+03	2742	0.29
COM3.1.6.6	1.30E+04	9.59E+02	6.71E+03	2530	0.27
COM3.1.7.6	8.23E+03	5.32E+02	3.71E+03	2473	0.29
COM3.2.1.6	9.54E+03	4.93E+02	4.13E+03	2567	0.28
COM3.2.2.6	6.70E+03	3.79E+02	3.12E+03	2981	0.30
COM3.2.3.6	7.43E+03	3.53E+02	3.29E+03	3076	0.31
COM3.2.4.6	7.40E+03	1.80E+02	3.06E+03	3172	0.29

COM3.2.5.6	3.93E+03	2.47E+02	1.89E+03	2936	0.34
COM3.2.6.6	6.71E+03	2.27E+02	3.29E+03	3008	0.30
COM3.2.7.6	5.25E+03	1.33E+02	2.21E+03	3137	0.32
RES1.1.6	4.06E+03	1.93E+02	1.80E+03	3153	0.36
RES1.2.6	5.54E+03	2.07E+02	2.72E+03	3385	0.33
RES1.3.6	4.64E+03	1.60E+02	2.21E+03	3230	0.33
RES1.4.6	4.43E+03	1.80E+02	2.08E+03	3198	0.33
RES1.5.6	4.46E+03	1.73E+02	2.10E+03	3146	0.33
RES1.6.6	4.25E+03	1.80E+02	2.18E+03	2902	0.32
RES1.7.6	4.98E+03	9.30E+01	2.48E+03	2887	0.31
RES2.1.6	3.89E+03	4.70E+01	1.84E+03	3057	0.34
RES2.2.6	4.49E+03	9.30E+01	2.17E+03	3318	0.33
RES2.3.6	4.33E+03	6.70E+01	2.09E+03	3159	0.33
RES2.4.6	4.04E+03	1.20E+02	1.90E+03	3159	0.34
RES2.5.6	4.51E+03	8.70E+01	2.19E+03	3434	0.32
RES2.6.6	4.35E+03	6.00E+01	2.14E+03	3060	0.32
RES2.7.6	3.86E+03	1.20E+02	1.75E+03	3062	0.33
RES3.1.6	6.50E+03	5.46E+02	3.62E+03	3499	0.33
RES3.2.6	6.33E+03	1.80E+02	2.90E+03	3642	0.32
RES3.3.6	5.59E+03	2.46E+02	2.72E+03	3457	0.33
RES3.4.6	5.53E+03	1.40E+02	2.72E+03	3194	0.32
RES3.5.6	5.22E+03	2.13E+02	2.44E+03	3271	0.33
RES3.6.6	6.19E+03	1.80E+02	3.02E+03	3285	0.32
RES3.7.6	5.11E+03	1.33E+02	2.46E+03	3304	0.32
RES4.1.6	4.90E+03	8.70E+01	2.35E+03	3068	0.32
RES4.2.6	6.07E+03	1.13E+02	2.68E+03	3516	0.31
RES4.3.6	5.19E+03	1.13E+02	2.45E+03	3219	0.33
RES4.4.6	4.71E+03	1.13E+02	2.33E+03	3199	0.34

RES4.5.6	5.45E+03	8.00E+01	2.70E+03	3198	0.31
RES4.6.6	5.02E+03	8.00E+01	2.70E+03	2978	0.32
RES4.7.6	4.54E+03	4.00E+01	2.09E+03	2866	0.32
COM1.1.1.5	7.05E+05	2.89E+05	1.77E+05	1468	0.21
COM1.1.2.5	4.18E+03	3.13E+02	1.80E+03	2825	0.33
COM1.1.3.5	3.41E+03	2.33E+02	1.59E+03	2667	0.33
COM1.1.4.5	3.45E+03	2.20E+02	1.63E+03	2721	0.33
COM1.1.5.5	3.72E+03	2.33E+02	1.81E+03	2784	0.33
COM1.1.6.5	3.08E+03	2.80E+02	1.57E+03	2556	0.33
COM1.1.7.5	9.05E+03	1.26E+03	4.14E+03	2241	0.28
COM1.2.1.5	8.42E+03	5.80E+02	3.69E+03	2440	0.29
COM1.2.2.5	3.86E+03	1.87E+02	1.71E+03	2777	0.33
COM1.2.3.5	3.20E+03	1.47E+02	1.60E+03	2604	0.33
COM1.2.4.5	3.26E+03	1.80E+02	1.46E+03	2606	0.34
COM1.2.5.5	3.55E+03	1.40E+02	1.54E+03	2833	0.34
COM1.2.6.5	3.48E+03	1.13E+02	1.65E+03	2895	0.35
COM1.2.7.5	1.47E+04	8.66E+02	5.53E+03	2009	0.24
COM2.1.1.5	3.18E+05	4.03E+04	1.81E+05	1630	0.18
COM2.1.2.5	2.13E+04	4.78E+03	9.52E+03	2229	0.24
COM2.1.3.5	1.59E+04	2.75E+03	7.48E+03	2552	0.25
COM2.1.4.5	1.35E+04	2.50E+03	6.27E+03	2755	0.26
COM2.1.5.5	1.49E+04	1.64E+03	6.93E+03	3102	0.28
COM2.1.6.5	1.25E+04	1.78E+03	5.90E+03	2778	0.27
COM2.1.7.5	1.32E+04	1.90E+03	6.10E+03	2859	0.27
COM2.2.1.5	1.57E+04	3.45E+03	8.30E+03	2341	0.27
COM2.2.2.5	3.66E+03	7.59E+02	1.37E+03	3045	0.34
COM2.2.3.5	3.64E+03	6.86E+02	1.41E+03	3084	0.33
COM2.2.4.5	3.47E+03	6.06E+02	1.31E+03	3092	0.34

COM2.2.5.5	3.51E+03	5.53E+02	1.15E+03	2986	0.33
COM2.2.6.5	2.92E+03	5.93E+02	1.20E+03	3011	0.35
COM2.2.7.5	9.34E+03	4.79E+02	3.97E+03	3103	0.29
COM3.1.1.5	9.16E+05	1.65E+05	4.71E+05	2547	0.23
COM3.1.2.5	2.02E+04	4.15E+03	8.87E+03	2760	0.27
COM3.1.3.5	9.11E+03	2.40E+03	4.48E+03	2814	0.29
COM3.1.4.5	5.62E+03	1.12E+03	2.62E+03	2825	0.30
COM3.1.5.5	5.04E+03	7.60E+02	2.24E+03	2874	0.31
COM3.1.6.5	4.56E+03	5.39E+02	2.00E+03	2828	0.31
COM3.1.7.5	8.42E+03	4.79E+02	3.59E+03	3010	0.29
COM3.2.1.5	1.17E+04	7.45E+02	4.85E+03	2837	0.27
COM3.2.2.5	3.31E+03	4.19E+02	1.44E+03	2702	0.33
COM3.2.3.5	2.79E+03	3.86E+02	1.13E+03	2855	0.35
COM3.2.4.5	3.66E+03	3.86E+02	1.39E+03	2883	0.32
COM3.2.5.5	3.54E+03	4.59E+02	1.54E+03	2894	0.32
COM3.2.6.5	4.09E+03	3.66E+02	1.60E+03	2775	0.31
COM3.2.7.5	7.28E+03	3.26E+02	2.86E+03	3204	0.31
RES1.1.5	4.59E+03	3.60E+02	2.21E+03	2506	0.31
RES1.2.5	4.40E+03	4.86E+02	2.12E+03	3035	0.33
RES1.3.5	6.05E+03	4.46E+02	2.58E+03	2968	0.34
RES1.4.5	5.00E+03	4.33E+02	2.30E+03	2903	0.31
RES1.5.5	4.16E+03	4.19E+02	1.95E+03	2675	0.32
RES1.6.5	6.71E+03	3.26E+02	2.43E+03	3179	0.35
RES1.7.5	4.85E+03	3.60E+02	2.18E+03	2724	0.32
RES2.1.5	2.41E+03	2.47E+02	1.13E+03	2973	0.35
RES2.2.5	3.63E+03	3.20E+02	1.25E+03	2974	0.34
RES2.3.5	2.46E+03	2.80E+02	1.05E+03	2663	0.34
RES2.4.5	2.13E+03	2.53E+02	8.93E+02	2538	0.34

RES2.5.5	2.78E+03	3.73E+02	1.15E+03	2730	0.33
RES2.6.5	2.32E+03	2.33E+02	9.79E+02	2837	0.34
RES2.7.5	2.18E+03	2.20E+02	9.05E+02	2845	0.35
RES3.1.5	4.57E+03	9.33E+02	2.54E+03	2945	0.34
RES3.2.5	3.90E+03	5.39E+02	1.77E+03	2789	0.32
RES3.3.5	3.18E+03	4.00E+02	1.46E+03	2760	0.33
RES3.4.5	3.24E+03	2.67E+02	1.63E+03	2705	0.33
RES3.5.5	3.82E+03	4.27E+02	1.76E+03	2810	0.33
RES3.6.5	2.59E+03	4.80E+02	1.38E+03	2498	0.34
RES3.7.5	3.86E+03	2.93E+02	1.75E+03	2721	0.32
RES4.1.5	3.55E+03	4.99E+02	1.77E+03	2322	0.32
RES4.2.5	3.34E+03	3.59E+02	1.55E+03	2585	0.33
RES4.3.5	3.40E+03	2.67E+02	1.69E+03	2927	0.35
RES4.4.5	3.18E+03	2.87E+02	1.43E+03	2707	0.33
RES4.5.5	3.30E+03	2.73E+02	1.57E+03	2700	0.32
RES4.6.5	3.41E+03	2.20E+02	1.61E+03	2621	0.32
RES4.7.5	2.56E+03	1.47E+02	1.14E+03	2607	0.34
COM1.1.1.4	8.20E+05	3.30E+05	3.83E+05	1769	0.22
COM1.1.2.4	4.37E+04	5.72E+03	2.12E+04	1862	0.22
COM1.1.3.4	1.54E+04	2.26E+03	7.56E+03	2343	0.27
COM1.1.4.4	1.26E+04	1.87E+03	5.71E+03	2497	0.28
COM1.1.5.4	1.19E+04	1.97E+03	5.64E+03	2642	0.28
COM1.1.6.4	1.04E+04	1.71E+03	4.74E+03	2885	0.30
COM1.1.7.4	1.41E+04	2.62E+03	7.07E+03	2520	0.27
COM1.2.1.4	1.70E+04	1.34E+03	7.04E+03	2299	0.25
COM1.2.2.4	1.41E+04	9.47E+02	5.81E+03	2431	0.26
COM1.2.3.4	1.58E+04	1.34E+03	6.57E+03	2176	0.26
COM1.2.4.4	1.68E+04	1.11E+03	7.37E+03	2130	0.26

COM1.2.5.4	1.41E+04	1.12E+03	6.12E+03	2275	0.26
COM1.2.6.4	1.39E+04	1.17E+03	5.95E+03	2322	0.27
COM1.2.7.4	1.96E+04	1.42E+03	7.52E+03	2104	0.26
COM2.1.1.4	2.64E+05	7.60E+04	9.00E+04	1826	0.20
COM2.1.2.4	2.23E+04	4.72E+03	7.42E+03	2252	0.25
COM2.1.3.4	1.81E+04	3.96E+03	7.52E+03	2890	0.28
COM2.1.4.4	1.45E+04	2.55E+03	6.11E+03	3127	0.29
COM2.1.5.4	1.18E+04	1.92E+03	4.68E+03	3011	0.29
COM2.1.6.4	1.48E+04	2.10E+03	6.14E+03	3136	0.29
COM2.1.7.4	1.25E+04	2.58E+03	4.99E+03	3035	0.29
COM2.2.1.4	1.57E+04	3.75E+03	8.52E+03	1928	0.26
COM2.2.2.4	9.74E+03	1.84E+03	3.27E+03	2962	0.29
COM2.2.3.4	8.88E+03	1.69E+03	3.53E+03	3343	0.31
COM2.2.4.4	8.79E+03	1.71E+03	3.10E+03	3269	0.30
COM2.2.5.4	8.66E+03	1.82E+03	3.27E+03	3084	0.30
COM2.2.6.4	9.48E+03	1.54E+03	3.46E+03	3164	0.30
COM2.2.7.4	9.89E+03	1.56E+03	4.10E+03	3190	0.31
COM3.1.1.4	9.57E+05	5.18E+05	3.21E+05	1786	0.20
COM3.1.2.4	5.24E+04	8.57E+03	1.70E+04	2087	0.24
COM3.1.3.4	2.60E+04	6.77E+03	9.55E+03	2351	0.26
COM3.1.4.4	2.15E+04	8.32E+03	7.78E+03	2016	0.25
COM3.1.5.4	2.35E+04	9.75E+03	7.67E+03	2065	0.25
COM3.1.6.4	1.96E+04	8.76E+03	5.96E+03	1777	0.24
COM3.1.7.4	2.17E+04	9.79E+03	6.54E+03	1722	0.23
COM3.2.1.4	8.29E+03	2.12E+03	4.18E+03	3206	0.32
COM3.2.2.4	6.53E+03	1.07E+03	2.31E+03	3130	0.32
COM3.2.3.4	4.68E+03	1.01E+03	1.37E+03	2872	0.34
COM3.2.4.4	5.35E+03	9.38E+02	1.85E+03	3057	0.33

COM3.2.5.4	7.18E+03	9.32E+02	2.62E+03	3045	0.31
COM3.2.6.4	7.32E+03	1.22E+03	2.56E+03	2993	0.31
COM3.2.7.4	7.40E+03	8.85E+02	2.74E+03	3284	0.32
RES1.1.4	6.50E+03	8.06E+02	3.10E+03	3081	0.32
RES1.2.4	3.44E+03	8.13E+02	1.44E+03	3250	0.35
RES1.3.4	4.54E+03	9.05E+02	2.01E+03	3242	0.34
RES1.4.4	3.42E+03	8.64E+02	1.36E+03	2933	0.36
RES1.5.4	3.40E+03	9.53E+02	1.31E+03	3057	0.34
RES1.6.4	3.56E+03	7.87E+02	1.27E+03	3162	0.35
RES1.7.4	6.08E+03	9.52E+02	2.89E+03	3176	0.32
RES2.1.4	4.19E+03	6.52E+02	1.94E+03	3784	0.36
RES2.2.4	2.87E+03	7.05E+02	9.99E+02	2898	0.35
RES2.3.4	2.65E+03	7.62E+02	6.76E+02	2644	0.34
RES2.4.4	2.49E+03	7.89E+02	6.30E+02	2343	0.33
RES2.5.4	3.05E+03	7.45E+02	9.39E+02	2596	0.34
RES2.6.4	2.64E+03	6.83E+02	7.99E+02	2839	0.35
RES2.7.4	3.96E+03	6.12E+02	1.49E+03	3422	0.35
RES3.1.4	1.24E+04	2.26E+03	6.75E+03	3057	0.31
RES3.2.4	5.21E+03	8.13E+02	1.91E+03	3274	0.33
RES3.3.4	5.12E+03	8.80E+02	2.11E+03	3344	0.33
RES3.4.4	4.82E+03	4.07E+02	1.83E+03	2867	0.31
RES3.5.4	5.45E+03	6.46E+02	2.13E+03	2990	0.32
RES3.6.4	5.50E+03	7.73E+02	2.27E+03	3130	0.32
RES3.7.4	5.84E+03	8.18E+02	2.41E+03	3037	0.31
RES4.1.4	7.53E+03	1.05E+03	3.67E+03	3209	0.31
RES4.2.4	6.60E+03	5.93E+02	2.58E+03	3087	0.30
RES4.3.4	8.15E+03	6.71E+02	2.44E+03	2686	0.29
RES4.4.4	6.35E+03	6.28E+02	2.45E+03	3075	0.32

RES4.5.4	5.58E+03	6.19E+02	2.27E+03	3074	0.32
RES4.6.4	5.77E+03	7.17E+02	2.41E+03	3175	0.33
RES4.7.4	7.14E+03	7.45E+02	2.81E+03	3143	0.30

Abbreviation: COM, commercial building; RES, residential household; Jun, June; Jul, July; Aug, August; Sep, September; Oct, October; Nov, November.
*Naming convention encompass (i) site type, (ii) site, (iii) time point, and (iv) month. See Example 1 and Example 2 below.
Example 1: COM3.2.1.6 (i) Commercial building (ii) site 3.2, (iii) time point 1, (iv) month 6
Example 2: RES4.7.6 (i) Residential household (ii) site 4, (iii) time point 7, (iv) month 6

Table S1: Continued

**Sample Id***	**Temperature logger (°C)**	**pH**	**Conductivity (µS.m^{-1})**	**Dissolved oxygen (mg.l^{-1})**	**Total chlorine (mg.l^{-1})**	**Ammonium (mg.l^{-1} as N)**	**Nitrate (mg.l^{-1}as N)**	**Nitrite (mg.l^{-1} as N)**	**Total organic carbon (mg.l^{-1} as TOC)**	**Total dissolved nitrogen (mg.l^{-1} as N)**
COM1.1.1.3	23.50	9.24	219.50	11.41	0.26	0.54	0.77	0.020	3.44	0.79
COM1.1.7.3	24.70	9.32	213.10	10.90	1.17	0.54	0.57	0.020	4.21	0.75
COM1.2.1.3	24.00	9.09	217.00	10.23	0.03	0.59	0.67	0.020	2.34	0.78
COM1.2.7.3	21.80	9.33	210.70	10.04	2.09	0.57	0.67	0.020	2.74	0.71
COM2.1.1.3	22.80	8.80	224.35	10.23	0.95	0.45	0.60	0.050	2.16	0.75
COM2.1.7.3	22.40	9.23	215.20	11.59	2.22	0.57	0.70	0.020	2.21	0.68
COM2.2.1.3	22.70	9.22	214.60	10.11	1.44	0.53	0.37	0.020	5.20	1.09
COM2.2.7.3	22.30	9.37	215.40	10.06	2.21	0.56	0.53	0.020	4.30	0.95
COM3.1.1.3	21.10	8.90	232.00	9.32	0.02	0.26	0.83	0.010	2.67	0.91
COM3.1.7.3	19.90	9.33	215.70	9.61	2.37	0.58	0.37	0.010	3.81	1.03
COM3.2.1.3	21.20	9.34	218.70	9.83	0.85	0.56	0.10	0.010	4.42	1.05
COM3.2.7.3	19.20	9.36	217.60	9.87	2.45	0.58	0.43	0.010	3.78	1.05
RES1.1.3	23.30	9.30	214.40	9.31	1.75	0.59	0.50	0.010	2.84	0.71
RES1.7.3	23.40	9.31	213.50	9.47	2.44	0.57	0.50	0.000	2.71	0.71
RES2.1.3	20.80	9.22	215.60	9.75	2.41	0.63	0.60	0.000	2.80	0.67
RES2.7.3	19.80	9.22	215.70	9.57	2.53	0.65	0.70	0.000	2.50	0.67
RES3.1.3	25.60	9.32	214.00	10.55	2.31	0.61	0.63	0.000	2.74	0.69
RES3.7.3	19.90	9.32	215.40	10.09	2.51	0.44	0.50	0.000	2.59	0.67

RES4.1.3	25.10	9.31	216.90	8.95	2.04	0.60	0.70	0.000	2.52	0.66
RES4.7.3	18.70	9.10	214.60	10.88	2.56	0.61	0.50	0.000	2.60	0.66
COM1.1.1.2	24.60	9.26	195.20	11.88	0.03	0.51	1.07	0.010	3.92	0.79
COM1.1.7.2	25.60	9.23	195.20	11.85	0.02	0.50	0.87	0.010	5.33	0.78
COM1.2.1.2	24.40	9.07	200.60	10.98	0.50	0.52	1.03	0.010	3.98	0.76
COM1.2.7.2	21.50	9.40	191.20	11.26	1.84	0.47	1.23	0.000	6.37	0.68
COM2.1.1.2	21.00	9.30	183.60	12.47	1.00	0.59	0.97	0.030	1.98	0.73
COM2.1.7.2	21.50	9.41	179.30	12.29	1.74	0.60	0.87	0.020	2.18	0.65
COM2.2.1.2	24.00	9.39	181.40	10.00	0.74	0.60	0.50	0.040	5.04	0.74
COM2.2.7.2	26.90	9.36	181.30	10.41	1.53	0.65	0.50	0.050	4.57	0.70
COM3.1.1.2	21.10	8.92	207.30	9.33	0.02	0.32	0.83	0.000	2.25	0.75
COM3.1.7.2	18.70	9.45	177.10	10.52	1.95	0.67	0.70	0.000	6.98	0.66
COM3.2.1.2	20.90	9.85	181.00	9.85	0.81	0.67	0.73	0.010	3.76	0.70
COM3.2.7.2	17.20	9.56	177.50	10.79	2.27	0.31	0.70	0.000	2.32	0.66
RES1.1.2	23.90	9.27	192.60	9.97	1.60	0.46	0.37	0.000	9.15	0.69
RES1.7.2	20.60	9.37	191.10	9.81	2.30	0.48	0.37	0.000	14.71	0.67
RES2.1.2	24.20	9.72	190.30	9.72	2.33	0.50	0.40	0.000	12.69	0.65
RES2.7.2	17.60	9.31	186.30	9.97	2.49	0.49	0.43	0.000	6.34	0.64
RES3.1.2	24.10	9.40	189.40	10.52	1.99	0.48	0.83	0.000	16.86	0.69
RES3.7.2	17.50	9.43	187.40	11.36	2.30	0.49	0.90	0.000	16.50	0.65
RES4.1.2	26.20	9.40	190.30	10.78	1.99	0.46	0.70	0.000	3.33	0.65
RES4.7.2	17.20	9.42	188.00	11.13	2.41	0.50	0.80	0.000	2.45	0.65
COM1.1.1.1	24.00	9.09	229.30	12.56	0.00	0.47	0.50	0.010	1.93	0.80
COM1.1.7.1	23.30	9.18	229.00	13.03	0.15	0.44	0.47	0.010	2.07	0.82
COM1.2.1.1	24.00	8.98	231.50	11.36	0.00	0.43	0.37	0.010	2.11	0.79
COM1.2.7.1	18.20	9.30	228.10	12.00	0.89	0.45	0.50	0.000	2.52	0.73
COM2.1.1.1	23.40	8.76	232.35	12.40	0.15	0.37	0.47	0.030	3.33	0.73
COM2.1.7.1	22.90	9.32	233.30	14.90	1.34	0.39	0.67	0.010	2.21	0.73
COM2.2.1.1	24.40	9.13	233.90	12.47	0.07	0.49	0.47	0.070	2.23	0.71
COM2.2.7.1	27.30	9.20	230.40	13.31	0.39	0.43	0.67	0.090	2.38	0.72
COM3.1.1.1	20.90	8.70	226.80	8.23	0.01	0.41	0.70	0.000	1.27	1.00

COM3.1.7.1	20.40	9.26	228.00	11.75	1.03	0.43	0.50	0.000	2.21	0.67
COM3.2.1.1	20.10	9.31	232.50	10.71	0.02	0.49	0.37	0.010	1.96	0.75
COM3.2.7.1	16.10	9.37	228.80	11.64	2.07	0.47	0.57	0.000	2.53	0.66
RES1.1.1	22.30	9.31	235.20	11.54	1.83	0.44	0.43	0.000	3.01	0.72
RES1.7.1	17.00	9.30	228.00	11.07	2.21	0.49	0.40	0.000	2.51	0.70
RES2.1.1	21.10	9.29	228.70	10.95	2.18	0.51	0.40	0.000	2.75	0.67
RES2.7.1	16.00	9.24	229.20	10.42	2.23	0.50	0.40	0.000	2.66	0.68
RES3.1.1	23.80	9.27	229.80	12.04	0.93	0.45	0.47	0.000	2.64	0.72
RES3.7.1	15.50	9.31	228.50	12.57	1.12	0.47	0.43	0.000	2.49	0.69
RES4.1.1	23.90	9.32	228.80	11.72	0.93	0.44	0.47	0.000	2.62	0.71
RES4.7.1	14.90	9.20	229.00	12.40	1.12	0.47	0.40	0.000	2.88	0.70
COM1.1.1.6	22.60	9.42	179.10	10.07	0.00	0.35	0.63	0.010	1.92	0.76
COM1.1.7.6	22.00	9.62	174.70	10.51	2.03	0.45	1.07	0.020	1.91	0.64
COM1.2.1.6	22.50	9.57	176.10	10.36	1.57	0.26	0.67	0.010	2.04	0.68
COM1.2.7.6	19.60	9.56	175.70	10.63	1.67	0.26	0.67	0.010	1.84	0.68
COM2.1.1.6	24.40	9.50	177.95	10.73	0.99	0.40	0.97	0.010	1.69	0.77
COM2.1.7.6	25.50	9.60	175.85	11.20	1.73	0.68	0.93	0.010	1.74	0.66
COM2.2.1.6	22.20	9.78	176.60	10.46	1.72	0.53	0.63	0.010	4.81	0.67
COM2.2.7.6	15.20	9.87	172.10	9.87	2.75	0.64	0.77	0.000	1.96	0.60
COM3.1.1.6	20.50	9.76	178.20	9.94	0.37	0.22	0.90	0.060	1.82	0.73
COM3.1.7.6	21.50	9.51	179.20	10.39	2.24	0.48	0.77	0.010	7.54	0.69
COM3.2.1.6	20.40	9.73	177.30	9.99	1.73	0.64	0.83	0.010	4.90	0.69
COM3.2.7.6	16.80	9.73	177.30	9.93	2.67	0.66	0.73	0.010	1.92	0.62
RES1.1.6	19.70	9.68	173.00	9.74	2.31	0.46	0.63	0.010	2.20	0.66
RES1.7.6	13.40	9.59	172.60	10.26	2.67	0.95	0.67	0.010	2.32	0.62
RES2.1.6	17.00	9.54	171.90	10.21	2.76	0.94	0.67	0.000	2.06	0.65
RES2.7.6	14.20	9.61	171.50	10.62	2.87	0.95	0.67	0.000	2.13	0.63
RES3.1.6	19.20	9.50	172.20	10.25	2.75	0.54	0.63	0.000	2.54	0.64
RES3.7.6	12.90	9.53	171.30	10.59	2.79	0.90	0.77	0.000	2.23	0.62
RES4.1.6	15.00	9.58	172.20	10.38	2.69	0.41	0.67	0.000	2.68	0.61
RES4.7.6	12.90	9.61	172.10	11.10	2.81	0.28	0.67	0.000	2.21	0.62

COM1.1.1.5	20.60	9.24	216.40	10.75	0.05	0.32	0.80	0.010	1.97	0.80
COM1.1.7.5	23.10	9.04	208.60	10.07	2.39	0.57	0.83	0.040	2.35	0.72
COM1.2.1.5	21.70	9.35	215.80	11.27	1.38	0.63	0.63	0.020	2.61	0.71
COM1.2.7.5	21.70	9.44	215.10	11.24	1.97	0.70	0.60	0.020	3.04	0.75
COM2.1.1.5	20.50	9.52	197.00	11.07	1.63	0.51	0.60	0.050	2.16	0.80
COM2.1.7.5	20.70	9.64	197.10	11.37	2.66	0.59	0.63	0.030	2.01	0.70
COM2.2.1.5	20.90	9.54	198.60	10.08	1.80	0.58	0.57	0.020	2.52	0.74
COM2.2.7.5	18.90	9.52	195.70	10.19	2.69	0.60	0.57	0.020	2.36	0.69
COM3.1.1.5	21.50	9.29	189.70	9.42	0.20	0.48	0.80	0.040	2.28	0.76
COM3.1.7.5	18.60	9.35	197.10	9.73	2.53	0.61	0.73	0.020	2.38	0.72
COM3.2.1.5	23.70	9.54	198.60	9.51	1.77	0.56	0.67	0.020	2.47	0.74
COM3.2.7.5	18.60	9.56	196.00	9.65	2.52	0.62	0.77	0.020	3.21	0.68
RES1.1.5	19.00	9.37	224.90	9.85	2.32	0.53	0.73	0.040	2.90	0.74
RES1.7.5	17.60	9.24	216.50	10.08	2.61	0.65	0.63	0.030	2.45	0.70
RES2.1.5	20.10	9.16	212.90	10.01	2.84	0.76	0.80	0.000	2.85	0.67
RES2.7.5	18.00	9.03	213.00	9.85	2.93	0.86	0.70	0.000	2.60	0.68
RES3.1.5	20.70	9.18	215.40	10.03	2.75	0.70	0.60	0.010	2.33	0.68
RES3.7.5	17.20	9.24	213.90	10.00	2.87	0.77	0.83	0.010	2.62	0.67
RES4.1.5	18.40	9.31	216.20	10.57	2.57	0.78	0.73	0.010	2.58	0.70
RES4.7.5	17.40	9.28	215.40	11.05	2.86	0.71	0.77	0.010	2.90	0.67
COM1.1.1.4	20.90	9.31	192.80	9.68	0.06	0.68	0.12	0.030	5.29	1.21
COM1.1.7.4	22.90	9.15	169.50	9.15	2.41	0.70	0.53	0.030	5.00	1.12
COM1.2.1.4	21.30	9.58	174.10	10.95	1.57	0.68	0.67	0.040	5.76	0.90
COM1.2.7.4	21.50	9.49	169.70	11.18	1.82	0.66	0.70	0.030	4.18	1.22
COM2.1.1.4	23.80	9.40	172.70	11.90	1.21	0.50	1.03	0.230	2.07	0.78
COM2.1.7.4	20.90	9.44	166.70	11.91	2.33	0.48	0.87	0.130	2.12	0.71
COM2.2.1.4	20.30	9.65	172.10	10.07	1.78	0.64	1.13	0.160	2.32	0.77
COM2.2.7.4	20.40	9.34	166.10	10.12	2.50	0.65	0.67	0.160	4.87	0.72
COM3.1.1.4	21.00	8.99	172.60	9.69	0.05	0.21	1.27	0.000	1.48	0.77
COM3.1.7.4	21.40	9.16	174.10	9.02	2.27	0.39	0.77	0.230	2.27	0.72
COM3.2.1.4	22.20	9.60	164.10	9.41	1.43	0.63	0.87	0.250	2.39	0.78

COM3.2.7.4	20.90	9.20	168.60	9.58	2.57	0.61	0.90	0.210	2.32	0.70
RES1.1.4	21.10	9.32	177.00	9.62	2.36	0.63	0.77	0.030	6.61	0.93
RES1.7.4	20.30	9.64	176.60	10.11	2.51	0.63	0.70	0.030	6.65	1.11
RES2.1.4	21.30	9.20	174.20	9.81	2.71	0.69	0.70	0.000	7.34	1.10
RES2.7.4	19.10	9.08	172.70	8.87	2.78	0.71	0.83	0.000	7.04	1.07
RES3.1.4	22.00	9.27	176.50	9.71	2.45	0.67	0.73	0.000	6.93	1.13
RES3.7.4	19.10	9.09	178.50	9.93	1.67	0.68	0.80	0.000	6.44	0.98
RES4.1.4	20.80	9.12	177.70	10.71	2.27	0.73	0.77	0.010	4.94	1.04
RES4.7.4	18.30	9.58	176.90	10.83	2.73	0.75	0.67	0.000	6.15	1.06

*Naming convention encompass (i) site type, (ii) site, (iii) time point, and (iv) month. See Example 1 and Example 2 below.
Example 1: COM3.2.1.6 (i) Commercial building (ii) site 3.2, (iii) time point 1, (iv) month 6
Example 2: RES4.7.6 (i) Residential household (ii) site 4, (iii) time point 7, (iv) month 6

Table S1: Continue

**Sample Id***	**Copper (µg.l^{-1})**	**Iron (µg.l^{-1})**	**Magnesium (µg.l^{-1})**	**Manganese (µg.l^{-1})**	**Lead (µg.l^{-1})**	**Zinc (µg.l^{-1})**	**Raw sequencing counts**	**Processed sequencing counts**	**Observed ASVs**	**Shannon diversity index**	**Pielou's evenness index**
COM1.1.1.3	170.14	23.70	1071.93	4.23	0.00	9.09	22527	13040	170	3.60	0.70
COM1.1.7.3	173.04	46.04	1355.79	5.54	0.00	5.24	25731	14663	271	4.21	0.75
COM1.2.1.3	259.53	33.66	1181.40	4.10	1.22	127.29	38579	21097	44	1.97	0.52
COM1.2.7.3	44.46	18.64	1075.93	4.26	0.00	0.00	33821	17882	566	4.76	0.75
COM2.1.1.3	116.27	24.22	1059.20	4.57	0.00	120.16	30002	16483	121	3.14	0.65
COM2.1.7.3	8.73	25.33	1062.48	4.46	0.00	0.00	34498	17443	885	5.89	0.87
COM2.2.1.3	145.24	10.05	1073.57	3.38	0.00	72.28	22181	11716	300	4.40	0.77
COM2.2.7.3	8.92	22.22	1123.39	4.29	0.00	0.00	25630	12568	710	5.68	0.86
COM3.1.1.3	266.28	42.56	1995.05	5.76	16.40	55.04	36065	21236	89	3.36	0.75
COM3.1.7.3	18.49	0.00	1084.48	4.22	0.64	0.00	34620	18356	549	4.73	0.75
COM3.2.1.3	239.50	10.21	1048.48	5.38	3.24	56.58	400	198	NA	NA	NA
COM3.2.7.3	14.75	0.00	1036.66	4.59	0.00	0.00	30008	15303	725	5.71	0.87
RES1.1.3	63.69	0.00	1185.95	2.82	1.11	0.00	32442	15778	581	5.40	0.85
RES1.7.3	4.86	0.00	1084.84	3.49	0.67	0.00	32248	15613	651	5.26	0.81

RES2.1.3	33.54	0.00	1131.76	3.31	6.58	0.00	26686	15009	649	5.55	0.86
RES2.7.3	0.00	13.74	1201.04	4.15	2.37	0.00	23791	13389	692	5.66	0.87
RES3.1.3	23.77	0.00	1040.84	3.31	0.00	0.00	30265	16454	765	5.65	0.85
RES3.7.3	0.00	0.00	1091.03	3.53	0.00	0.00	34193	16819	844	5.80	0.86
RES4.1.3	33.64	0.00	1068.48	3.14	0.00	0.00	28278	15142	659	5.49	0.85
RES4.7.3	0.00	0.00	1026.11	3.35	0.00	0.00	31358	16775	821	5.76	0.86
COM1.1.1.2	198.33	16.02	1096.12	3.54	0.00	21.43	34930	19677	91	3.20	0.71
COM1.1.7.2	171.42	21.06	1098.12	3.60	0.00	8.99	38848	23876	173	3.43	0.67
COM1.2.1.2	210.36	29.19	1194.31	4.22	1.20	226.59	28615	16913	48	2.38	0.61
COM1.2.7.2	58.84	31.82	1188.49	4.33	0.00	0.00	32207	17720	409	4.47	0.74
COM2.1.1.2	139.02	39.79	1241.77	4.57	0.75	74.42	33131	18532	123	2.38	0.49
COM2.1.7.2	18.33	27.16	1081.57	3.84	0.00	6.84	34550	18642	559	5.12	0.81
COM2.2.1.2	193.09	78.10	1111.21	4.49	0.00	83.11	470	228	NA	NA	NA
COM2.2.7.2	62.27	65.15	1070.11	4.44	0.00	0.00	33124	18248	650	5.42	0.84
COM3.1.1.2	307.99	31.09	1335.43	6.64	5.23	27.00	21829	12849	206	3.80	0.71
COM3.1.7.2	15.41	10.60	1119.39	3.98	0.00	0.00	28927	15844	562	5.11	0.81
COM3.2.1.2	175.68	14.29	1562.02	4.81	11.49	28.88	28261	18489	91	3.44	0.76
COM3.2.7.2	20.26	0.00	1139.40	3.70	0.00	0.00	39341	22043	330	4.02	0.69
RES1.1.2	45.81	37.88	1304.15	3.56	2.91	8.79	24323	12505	429	4.85	0.80
RES1.7.2	0.00	28.46	1214.50	3.89	0.00	0.00	24658	12463	533	5.04	0.80
RES2.1.2	27.00	21.38	1235.77	3.79	4.33	0.00	25246	14711	480	4.88	0.79
RES2.7.2	0.00	0.00	1180.13	3.83	1.57	0.00	23870	13366	481	5.00	0.81
RES3.1.2	40.28	11.38	1175.40	4.02	0.00	0.00	45	6	NA	NA	NA
RES3.7.2	0.00	11.14	1110.85	3.98	0.00	0.00	27241	15022	545	5.03	0.80
RES4.1.2	27.49	16.98	1106.30	4.28	0.00	0.00	30061	16477	482	4.93	0.80
RES4.7.2	0.00	18.93	1126.67	3.91	0.00	0.00	33938	17906	578	5.17	0.81
COM1.1.1.1	158.66	0.00	1075.52	3.34	0.00	17.14	31229	18819	54	2.82	0.71
COM1.1.7.1	130.66	0.00	1103.86	3.31	0.00	0.00	20486	13008	57	2.90	0.72
COM1.2.1.1	157.84	0.00	1119.07	4.12	1.52	455.11	4145	2833	NA	NA	NA
COM1.2.7.1	33.12	0.00	1130.85	3.36	0.00	0.00	28005	15048	338	4.50	0.77
COM2.1.1.1	58.11	0.00	1056.88	5.75	0.63	105.17	32825	19344	183	3.87	0.74

COM2.1.7.1	50.51	18.15	1074.63	6.85	0.00	20.45	34492	19863	446	4.63	0.76
COM2.2.1.1	193.06	65.93	1242.40	5.94	0.00	73.73	19184	11887	76	3.05	0.70
COM2.2.7.1	114.03	118.66	1095.80	6.93	0.75	6.00	19014	10973	284	4.61	0.82
COM3.1.1.1	203.44	0.00	1467.01	7.51	20.68	20.07	21870	14650	77	3.57	0.82
COM3.1.7.1	39.89	0.00	1355.30	7.85	1.54	0.00	32103	18921	157	3.39	0.67
COM3.2.1.1	328.47	61.02	1558.38	12.37	7.29	128.34	20797	12802	50	2.78	0.71
COM3.2.7.1	15.92	24.32	1332.34	5.74	1.16	0.00	24273	12599	467	4.97	0.81
RES1.1.1	33.93	0.00	1236.88	2.91	0.00	0.00	26753	13831	384	4.58	0.77
RES1.7.1	0.00	0.00	1122.79	2.98	0.00	11.15	22718	10295	383	4.76	0.80
RES2.1.1	0.00	0.00	1193.63	2.56	1.15	0.00	29465	15691	512	4.81	0.77
RES2.7.1	11.73	0.00	1166.34	2.59	6.66	0.00	24937	13499	458	4.71	0.77
RES3.1.1	21.30	0.00	1114.59	2.49	0.00	7.97	32342	17810	424	4.42	0.73
RES3.7.1	0.00	0.00	1101.17	2.70	2.17	0.00	29367	15541	506	4.98	0.80
RES4.1.1	18.07	0.00	1077.16	2.58	0.00	9.95	33634	18724	433	4.54	0.75
RES4.7.1	0.00	0.00	1148.89	3.02	0.00	0.00	27430	13496	470	4.91	0.80
COM1.1.1.6	174.52	33.06	938.32	2.82	0.00	0.00	30754	18672	36	2.18	0.61
COM1.1.7.6	174.52	33.06	938.32	2.82	0.00	0.00	22432	13089	415	4.75	0.79
COM1.2.1.6	115.08	56.55	926.10	7.36	0.00	0.00	16695	10726	299	4.74	0.83
COM1.2.7.6	115.08	56.55	926.10	7.36	0.00	0.00	23246	14251	379	4.94	0.83
COM2.1.1.6	98.60	0.00	1001.70	4.03	1.02	98.15	39588	23349	175	3.48	0.67
COM2.1.7.6	98.60	0.00	1001.70	4.03	1.02	98.15	32590	20096	356	4.68	0.80
COM2.2.1.6	69.29	0.00	917.15	6.58	0.00	0.00	1623	803	NA	NA	NA
COM2.2.7.6	69.29	0.00	917.15	6.58	0.00	0.00	21513	12606	639	5.05	0.78
COM3.1.1.6	88.92	0.00	829.61	1.97	3.39	0.00	26016	16922	90	3.30	0.73
COM3.1.7.6	88.92	0.00	829.61	1.97	3.39	0.00	17064	10321	332	4.42	0.76
COM3.2.1.6	129.84	0.00	921.77	10.54	2.82	0.00	2048	1110	NA	NA	NA
COM3.2.7.6	129.84	0.00	921.77	10.54	2.82	0.00	13276	7632	NA	NA	NA
RES1.1.6	40.74	0.00	1188.68	4.40	0.00	0.00	10543	5955	NA	NA	NA
RES1.7.6	0.00	0.00	1016.84	6.07	0.00	0.00	15478	8994	443	4.31	0.71
RES2.1.6	31.28	0.00	1071.93	4.17	3.18	0.00	15970	9289	376	3.98	0.67
RES2.7.6	0.00	0.00	1134.30	5.50	0.85	0.00	12777	7578	NA	NA	NA

RES3.1.6	6.31	0.00	1048.11	5.86	0.00	0.00	19553	11665	411	4.20	0.70
RES3.7.6	0.00	0.00	1087.39	5.62	0.00	0.00	16465	9266	478	4.46	0.72
RES4.1.6	17.08	0.00	1084.66	5.16	0.00	0.00	17093	9993	453	4.23	0.69
RES4.7.6	0.00	0.00	914.61	5.86	0.00	0.00	15324	8906	478	4.47	0.72
COM1.1.1.5	171.62	0.00	896.46	2.88	0.00	117.64	46723	24969	42	2.35	0.63
COM1.1.7.5	31.72	0.00	1022.66	3.44	0.00	0.00	31303	16243	641	5.17	0.80
COM1.2.1.5	93.20	0.00	1000.29	2.71	0.00	0.00	9869	5146	NA	NA	NA
COM1.2.7.5	74.05	0.00	979.38	2.91	1.80	7.52	22279	12523	402	5.04	0.84
COM2.1.1.5	67.78	0.00	990.10	3.80	0.00	96.88	37091	19144	152	3.19	0.63
COM2.1.7.5	9.34	0.00	1005.92	4.53	0.00	0.00	29983	15113	887	5.85	0.86
COM2.2.1.5	167.02	0.00	1031.02	3.13	0.00	15.99	14993	8534	NA	NA	NA
COM2.2.7.5	7.08	0.00	963.37	4.94	0.00	0.00	27323	13325	954	5.95	0.87
COM3.1.1.5	447.99	69.85	984.47	35.19	11.07	16.05	15123	8952	NA	NA	NA
COM3.1.7.5	12.30	0.00	993.01	4.71	0.00	0.00	23666	12708	764	5.67	0.85
COM3.2.1.5	196.77	0.00	979.74	2.72	1.14	47.62	13580	7710	NA	NA	NA
COM3.2.7.5	12.90	0.00	931.55	4.86	0.00	0.00	22245	12111	762	5.62	0.85
RES1.1.5	26.24	0.00	1149.21	4.50	0.65	0.00	27792	14778	856	5.60	0.83
RES1.7.5	0.00	0.00	1011.02	3.54	0.00	0.00	20828	10824	727	5.59	0.85
RES2.1.5	22.69	0.00	1076.48	4.74	1.00	0.00	21370	11704	702	5.45	0.83
RES2.7.5	0.00	0.00	1098.48	5.23	0.74	0.00	24889	13284	930	5.85	0.86
RES3.1.5	12.73	0.00	1047.93	4.08	1.49	0.00	27791	15465	716	5.17	0.79
RES3.7.5	0.00	0.00	1044.11	4.09	0.00	0.00	19157	10354	728	5.56	0.84
RES4.1.5	19.03	0.00	1049.20	3.39	0.00	0.00	25385	14543	755	5.45	0.82
RES4.7.5	5.42	0.00	1042.66	4.68	0.00	0.00	20043	11015	721	5.53	0.84
COM1.1.1.4	190.29	27.42	1052.84	3.31	0.97	131.64	33980	18250	51	2.59	0.66
COM1.1.7.4	33.08	32.79	1086.30	6.28	0.00	0.00	30397	15726	602	5.09	0.79
COM1.2.1.4	120.98	36.35	1000.65	5.33	0.00	0.00	17495	9956	297	4.49	0.79
COM1.2.7.4	85.75	65.88	1046.47	6.35	0.00	0.00	21404	12170	420	5.13	0.85
COM2.1.1.4	189.57	86.77	2211.31	8.79	0.72	302.92	29480	17402	215	3.56	0.66
COM2.1.7.4	16.46	0.00	1019.93	4.69	0.00	0.00	20552	11383	675	5.69	0.87
COM2.2.1.4	183.38	0.00	1091.39	3.53	0.00	26.27	8390	4559	NA	NA	NA

COM2.2.7.4	7.90	0.00	974.10	5.45	0.00	0.00	21458	11508	650	5.56	0.86
COM3.1.1.4	147.69	0.00	1069.75	3.77	6.33	7.37	34314	21630	93	3.40	0.75
COM3.1.7.4	15.51	0.00	923.19	4.24	0.00	0.00	17659	10233	360	3.94	0.67
COM3.2.1.4	186.61	0.00	929.73	2.08	1.40	36.38	8906	4977	NA	NA	NA
COM3.2.7.4	13.97	0.00	927.55	4.50	0.00	0.00	22867	12156	638	5.44	0.84
RES1.1.4	27.77	0.00	944.28	4.50	0.70	0.00	24493	11494	654	5.48	0.85
RES1.7.4	0.00	0.00	935.01	4.34	0.70	0.00	23948	12630	692	5.46	0.84
RES2.1.4	35.34	0.00	1022.11	4.14	5.43	0.00	18214	9998	449	4.79	0.78
RES2.7.4	0.00	0.00	985.19	4.79	1.31	0.00	14175	8045	NA	NA	NA
RES3.1.4	44.36	0.00	1041.02	5.46	0.00	0.00	20364	11282	463	4.98	0.81
RES3.7.4	0.00	0.00	1009.02	4.93	0.00	0.00	20208	10942	644	5.46	0.84
RES4.1.4	22.91	0.00	1048.47	4.84	0.00	0.00	23586	12786	641	5.34	0.83
RES4.7.4	0.00	0.00	1021.20	6.37	0.00	0.00	25842	13172	751	5.61	0.85

*Naming convention encompass (i) site type, (ii) site, (iii) time point, and (iv) month. See Example 1 and Example 2 below.
Example 1: COM3.2.1.6 (i) Commercial building (ii) site 3.2, (iii) time point 1, (iv) month 6
Example 2: RES4.7.6 (i) Residential household (ii) site 4, (iii) time point 7, (iv) month 6

Table S2: Reference 16S rRNA strains and outgroups with associated accession numbers of Nitrosomonas, Nitrospira, Mycobacterium, Legionella, and Pseudomonas that were downloaded from NCBI and used to reconstruct maximum likelihood phylogenetic trees for selected ASVs.

**Genus**	**Reference sequence**	**Accession number**
Legionella	*Legionella geestiana*	NR044957
Legionella	*Legionella pneumophila*	NR074231
Legionella	*Legionella parisiensis*	LC504039
Legionella	*Legionella beliardensis*	NR024968
Legionella	*Legionella israelensis*	NR036894
Legionella	*Legionella massiliensis*	NR109415
Legionella	*Legionella birminghamensis*	LC504038
Legionella outgroup	*Coxiella burnetii*	NR104916
Mycobacterium	*Mycobacterium montefiorense*	NR028808
Mycobacterium	*Mycobacterium lentiflavum*	AF480583
Mycobacterium	*Mycobacterium gordonae*	X52923
Mycobacterium	*Mycobacterium shinjukuense*	AB268503
Mycobacterium	*Mycobacterium intracellulare*	AJ536036
Mycobacterium	*Mycobacterium avium*	AJ536037
Mycobacterium	*Mycobacterium persicum*	NR156953
Mycobacterium	*Mycobacterium abscessus*	AY859681
Mycobacterium	*Mycobacterium chelonae*	AY457072
Mycobacterium	*Mycobacterium neglectum*	MF769621
Mycobacterium	*Mycobacterium tusciae*	AF058299
Mycobacterium	*Mycobacterium fortuitum*	AY457066
Mycobacterium	*Mycobacterium iranicum*	HQ009482
Mycobacterium	*Mycobacterium sediminis*	KC010490
Mycobacterium	*Mycobacterium frederiksbergense*	AJ276274
Mycobacterium outgroup	*Corynebacterium glutamicum*	NR041817
Nitrosomonas	*Nitrosomonas halophila*	FR828475
Nitrosomonas	*Nitrosomonas eutropha*	NR027566
Nitrosomonas	*Nitrosomonas stercoris*	AB900133
Nitrosomonas	*Nitrosomonas mobilis*	LC053403
Nitrosomonas	*Nitrosomonas nitrosa*	FR828477
Nitrosomonas	*Nitrosomonas cryotolerans*	AF272423
Nitrosomonas	*Nitrosomonas marina*	FR828473
Nitrosomonas	*Nitrosomonas aestuarii*	NR104818
Nitrosomonas	*Nitrosomonas ureae*	FR828472
Nitrosomonas	*Nitrosomonas oligotropha*	NR1048200
Nitrosomonas outgroup	*Methyloversatilis thermotolerans*	KC782839
Nitrospira	*Ca. Nitrospira salsa*	KC706459
Nitrospira	*Nitrospira marina*	HQ686084
Nitrospira	*Nitrospira calida*	HM485589

Nitrospira	*Ca. Nitrospira bockiana*	EU084879
Nitrospira	*Ca. Nitrospira defluvii*	DQ059545
Nitrospira	*Ca. Nitrospira Inopinata*	LN885086
Nitrospira	*Nitrospira moscoviensis*	JQ073807
Nitrospira	*Nitrospira lenta*	KF724505
Nitrospira	*Nitrospira japonica*	AB818959
Nitrospira outgroup	*Leptospirillum ferrooxidans*	X86776
Pseudomonas	*Pseudomonas aeruginosa*	NR026078
Pseudomonas	*Pseudomonas peli*	MN752883
Pseudomonas	*Pseudomonas synxantha*	NR043425
Pseudomonas	*Pseudomonas thivervalensis*	KU921578
Pseudomonas	*Pseudomonas fluorescens*	GU198112
Pseudomonas	*Pseudomonas veronii*	NR028706
Pseudomonas	*Pseudomonas psychrophila*	NR028619
Pseudomonas	*Pseudomonas congelans*	MN826588
Pseudomonas	*Pseudomonas monsensis*	CP077087
Pseudomonas	*Pseudomonas neuropathica*	OK513263
Pseudomonas outgroup	*Acinetobacter calcoaceticus*	X81668

Table S3: Water demand in cubic meter per month associated with three commercial buildings and four residential household sites from January 2019 – November 2020.

Month-year	COM1	COM2	COM3	RES1	RES2	RES3	RES4
Jan-19	26,450	15,420	7,970	703	1,535	1,308	1,405
Feb-19	39,350	24,000	9,780	767	1,300	1,207	1,315
Mar-19	26,400	19,060	8,950	911	1,777	1,212	1,431
Apr-19	40,650	19,540	13,630	740	1,221	1,319	1,480
May-19	29,700	19,080	21,780	866	1,151	1,385	1,450
Jun-19	64,800	24,470	47,660	901	1,218	1,208	1,471
Jul-19	102,250	26,170	50,250	1,022	1,006	1,102	1,411
Aug-19	147,950	36,470	67,550	710	970	1,138	1,446
Sep-19	64,650	18,700	26,400	631	827	947	1,300
Oct-19	82,200	31,870	33,980	729	894	1,102	1,310
Nov-19	34,650	18,720	16,700	628	859	1,363	1,280
Dec-19	23,650	19,580	11,540	755	682	1,483	1,090
Jan-20	23,400	13,920	8,510	670	1,059	1,636	1,140
Feb-20	29,400	22,580	11,500	777	1,121	1,854	1,370
Mar-20	25,450	15,680	9,200	743	1,099	1,757	1,460
Apr-20	15,650	7,480	6,300	900	984	1,766	1,500
May-20	17,450	8,230	10,910	974	1,160	1,987	1,510
Jun-20	71,750	15,470	37,840	933	1,058	1,835	1,510
Jul-20	70,950	21,190	91,540	1,081	856	1,693	1,580
Aug-20	76,770	39,620	83,840	885	1,379	1,853	1,680
Sep-20	65,850	45,240	35,910	748	1,402	2,253	1,530
Oct-20	46,000	48,020	37,210	757	1,499	2,230	1,580
Nov-20	25,700	42,710	41,180	810	948	2,072	1,890

Abbreviation: COM1, commercial building 1; COM2, commercial building 2; COM3, commercial building 3; RES1, residential household 1; RES2, residential household 2; RES3, residential household 3; RES4, residential household 4.

Table S4: Descriptive statistics and independent t-test (or Mann-Whitney U test) to compare the means (or medians) of water chemistry, flow cytometric, and 16S rRNA gene sequencing data that were documented the first month of phased-reopening between (A) site types (i.e., commercial building vs. residential household) and (B) first draw (TP_0) and final flushed (TP_{30}) samples of each site type.

A. Site types

**Parameter**	**Commercial building (mean ± SD, median)**	**Residential household (mean ± SD, median)**	**Shapiro–Wilk test**	**Independent t-test or Mann-Whitney U test**
TCC (cells/ml)	295000 ± 367000, 121136	11000 ± 5870, 8968	W = 0.61119, p-value = 2.512e-12	W = 1154, p-value = 3.224e-16
Intact cell proportions (%)	29.74 ± 20.29, 33.86	3.61 ± 7.04, 1.26	W = 0.79027, p-value = 1.329e-08	W = 1106, p-value = 2.042e-12
Phenotypic diversity index (D2)	2126 ± 350, 2168	2905 ± 381, 2835	W = 0.97775, p-value = 0.00247	W = 70, p-value = 2.042e-12
Evenness	0.23 ± 0.02, 0.23	0.29 ± 0.01, 0.29	W = 0.95123, p-value = 0.008417	W = 19, p-value < 2.2e-16
Observed species	199 ± 159, 157	446 ± 49, 445	W = 0.85023, p-value = 0.006766	W = 9, p-value = 0.00254
Shannon diversity index	3.73 ± 0.82, 3.57	4.71 ± 0.19, 4.74	W = 0.83198, p-value = 0.003462	W = 14, p-value = 0.01207
Pielou's evenness index	0.75 ± 0.05, 0.74	0.77 ± 0.03, 0.77	W = 0.95359, p-value = 0.4538	t = -1.2647, p-value = 0.223
Temperature (°C)	22.42 ± 3.32, 23.08	19.09 ± 3.78, 18.15	W = 0.94421, p-value = 0.2876	t = 2.082, p-value = 0.05189
pH	9.13 ± 0.22, 9.19	9.28 ± 0.04, 9.3	W = 0.75943, p-value = 0.0002278	W = 28.5, p-value = 0.1416
Conductivity ($\mu S.m^{-1}$)	230.33 ± 2.33, 229.85	229.65 ± 2.3, 228.9	W = 0.89518, p-value = 0.03353	W = 57.5, p-value = 0.487
Dissolved oxygen ($mg.l^{-1}$)	12.03 ± 1.61, 12.2	11.59 ± 0.75, 11.63	W = 0.93726, p-value = 0.2127	t = 0.72238, p-value = 0.4793
Total chlorine ($mg.l^{-1}$)	0.51 ± 0.68, 0.15	1.57 ± 0.6, 1.48	W = 0.87519, p-value = 0.01451	W = 11, p-value = 0.004798
Ammonium ($mg.l^{-1}$ as N)	0.44 ± 0.04, 0.44	0.47 ± 0.03, 0.47	W = 0.96008, p-value = 0.5454	t = -2.0788, p-value = 0.05221
Nitrate ($mg.l^{-1}$ as N)	0.52 ± 0.11, 0.5	0.43 ± 0.03, 0.42	W = 0.85336, p-value = 0.00607	W = 77, p-value = 0.02574
Nitrite ($mg.l^{-1}$ as N)	0.02 ± 0.03, 0.01	0 ± 0, 0	W = 0.5504, p-value = 9.401e-07	W = 80, p-value = 0.005611

Total organic carbon (mg.l^{-1} as TOC)	2.23 ± 0.48, 122.34	2.69 ± 0.18, 5.87	W = 0.97048, p-value = 0.7649	t = -2.5936, p-value = 0.01834
Total nitrogen (mg.l^{-1} as N)	0.76 ± 0.09, 0	0.7 ± 0.02, 0	W = 0.75441, p-value = 0.0001946	W = 77, p-value = 0.02524
Copper (µg.l^{-1})	123.64 ± 91.52, 1124.96	10.63 ± 12.9, 1135.84	W = 0.82203, p-value = 0.001877	W = 92, p-value = 0.000755
Iron (µg.l^{-1})	24.01 ± 38.32, 5.85	0 ± 0, 2.65	W = 0.54028, p-value = 7.518e-07	W = 68, p-value = 0.04801
Magnesium (µg.l^{-1})	1217.67 ± 171.24, 0.69	1145.18 ± 52.4, 0	W = 0.81041, p-value = 0.00124	W = 50, p-value = 0.9101
Manganese (µg.l^{-1})	6.09 ± 2.57, 18.61	2.73 ± 0.21, 0	W = 0.81081, p-value = 0.001258	W = 96, p-value = 1.588e-05
Lead (µg.l^{-1})	2.8 ± 5.98, 2.21	1.25 ± 2.33, 2.65	W = 0.50686, p-value = 3.669e-07	W = 56.5, p-value = 0.5096
Zinc (µg.l^{-1})	68.83 ± 129.47, 0.73	3.63 ± 5.09, 0.7	W = 0.46308, p-value = 1.499e-07	W = 71, p-value = 0.06877

Abbreviation: TCC, total cell concentrations.

B. First draw (TP_0) and final flushed (TP_{30}) samples of each site type.

Site type	Parameter	TP_0 (mean ± SD, median)	TP_{30} (mean ± SD, median)	Shapiro–Wilk test	Independent t-test or Wilcoxon signed-rank test
COM	TCC (cells/ml)	807135 ± 391315, 916437	100172 ± 94698, 56634	W = 0.8462, p-value = 0.03296	W = 36, p-value = 0.002165
COM	Intact cell proportions (%)	52.98 ± 3.42, 53.28	24.6 ± 14.22, 30.00	W = 0.85865, p-value = 0.04702	W = 36, p-value = 0.002165
COM	Phenotypic diversity index (D2)	1938 ± 163, 1947	2551 ± 391, 2398	W = 0.91391, p-value = 0.2394	t = -3.5431, p-value = 0.00533
COM	Observed species	88 ± 54, 76	292 ± 161, 311	W = 0.84563, p-value = 0.03743	W = 4, p-value = 0.05195
COM	Evenness	0.21 ± 0.02, 0.20	0.24 ± 0.02, 0.23	W = 0.9659, p-value = 0.8634	t = -2.8335, p-value = 0.01774
COM	Shannon diversity index	3.22 ± 0.48, 3.05	4.16 ± 0.82, 4.55	W = 0.89054, p-value = 0.1412	t = -2.2588, p-value = 0.05027
COM	Pielou's evenness index	0.74 ± 0.05, 0.71	0.76 ± 0.06, 0.77	W = 0.92777, p-value = 0.3887	t = -0.60118, p-value = 0.5626
RES	TCC (cells/ml)	20695 ± 12036, 18979	11811 ± 1181, 11936	W = 0.75704, p-value = 0.009768	W = 12, p-value = 0.3429

RES	Intact cell proportions (%)	17.06 ± 12.68, 19.40	2 ± 1.19, 1.61	W = 0.72783, p-value = 0.004631	W = 14, p-value = 0.1143
RES	Phenotypic diversity index (D2)	2931 ± 610, 3002	3521 ± 310, 3564	W = 0.89844, p-value = 0.2797	t = -1.7252, p-value = 0.1352
RES	Observed species	438 ± 54, 428	454 ± 52, 464	W = 0.92778, p-value = 0.4961	t = -0.4298, df = 6, p-value = 0.6823
RES	Evenness	0.28 ± 0.03, 0.28	0.29 ± 0.02, 0.29	W = 0.94844, p-value = 0.6955	t = -1.0586, p-value = 0.3305
RES	Shannon diversity index	4.58 ± 0.16, 4.56	4.84 ± 0.13, 4.84	W = 0.97492, p-value = 0.9336	t = -2.4962, p-value = 0.05677
RES	Pielou's evenness index	0.76 ± 0.06, 0.76	0.79 ± 0.02, 0.880	W = 0.8939, p-value = 0.2543	t = -3.0567, p-value = 0.05232

Abbreviation: COM, commercial building; RES, residential household; TCC, total cell concentrations.

Table S5: Mean relative abundance selected phyla, family, and genera associated with TP_0 and TP_{30} June samples of the residential household and commercial building sites.

Site type	Time point	Taxonomic level	Taxonomic classification	Relative abundance (mean ± SD, median)
COM	TP_0	Phylum	*Actinobacteria*	1.07 ± 0.83
COM	TP_0	Phylum	*Bacteroidetes*	5.48 ± 6.64
COM	TP_0	Phylum	*Cyanobacteria*	1.36 ± 2.07
COM	TP_0	Phylum	*Firmicutes*	0.02 ± 0.04
COM	TP_0	Phylum	*Gemmatimonadetes*	3.21 ± 2.46
COM	TP_0	Phylum	*Nitrospirae*	1.24 ± 2.39
COM	TP_0	Phylum	*Planctomycetes*	1.51 ± 1.84
COM	TP_0	Phylum	*Proteobacteria*	82.62 ± 14.27
COM	TP_0	Phylum	*TM6_(Dependentiae)*	2.63 ± 5.31
COM	TP_0	Phylum	Rare	0.85
COM	TP_0	Phylum	Unclassified	0.01 ± 0.02
COM	TP_{30}	Phylum	*Actinobacteria*	0.74 ± 0.46
COM	TP_{30}	Phylum	*Bacteroidetes*	7.51 ± 4.29
COM	TP_{30}	Phylum	*Cyanobacteria*	0.72 ± 0.7
COM	TP_{30}	Phylum	*Firmicutes*	1.21 ± 1.35
COM	TP_{30}	Phylum	*Gemmatimonadetes*	0.86 ± 0.98
COM	TP_{30}	Phylum	*Nitrospirae*	0.79 ± 0.8
COM	TP_{30}	Phylum	*Planctomycetes*	1.96 ± 1.97
COM	TP_{30}	Phylum	*Proteobacteria*	82.09 ± 8.98
COM	TP_{30}	Phylum	*TM6_(Dependentiae)*	2.93 ± 6.33
COM	TP_{30}	Phylum	Rare	1.18
COM	TP_{30}	Phylum	Unclassified	0.01 ± 0.02
RES	TP_0	Phylum	*Actinobacteria*	4.38 ± 6.28
RES	TP_0	Phylum	*Bacteroidetes*	11.11 ± 3.47
RES	TP_0	Phylum	*Cyanobacteria*	0.68 ± 0.71
RES	TP_0	Phylum	*Firmicutes*	3.21 ± 1.78
RES	TP_0	Phylum	*Gemmatimonadetes*	0.01 ± 0.01
RES	TP_0	Phylum	*Nitrospirae*	0.11 ± 0.03
RES	TP_0	Phylum	*Planctomycetes*	1.81 ± 2.13
RES	TP_0	Phylum	*Proteobacteria*	77.3 ± 5.85
RES	TP_0	Phylum	*TM6_(Dependentiae)*	0.03 ± 0.02
RES	TP_0	Phylum	Rare	1.36
RES	TP_0	Phylum	Unclassified	0.02 ± 0.02
RES	TP_{30}	Phylum	*Actinobacteria*	0.33 ± 0.12
RES	TP_{30}	Phylum	*Bacteroidetes*	16.34 ± 1.5
RES	TP_{30}	Phylum	*Cyanobacteria*	0.31 ± 0.24
RES	TP_{30}	Phylum	*Firmicutes*	4.19 ± 0.37

RES	TP_{30}	Phylum	*Gemmatimonadetes*	0.02 ± 0.03
RES	TP_{30}	Phylum	*Nitrospirae*	0.16 ± 0.06
RES	TP_{30}	Phylum	*Planctomycetes*	0.92 ± 0.43
RES	TP_{30}	Phylum	*Proteobacteria*	75.55 ± 2.11
RES	TP_{30}	Phylum	*TM6_(Dependentiae)*	0.09 ± 0.07
RES	TP_{30}	Phylum	Rare	2.05
RES	TP_{30}	Phylum	Unclassified	0.04 ± 0.03
COM	TP_0	Proteobacteria class	*Alphaproteobacteria*	54.52 ± 16.08
COM	TP_0	Proteobacteria class	*Betaproteobacteria*	27.94 ± 11.41
COM	TP_0	Proteobacteria class	*Gammaproteobacteria*	0.11 ± 0.14
COM	TP_0	Proteobacteria class	Other	14.77
COM	TP_0	Proteobacteria class	Unclassified	2.65 ± 5.3
COM	TP_{30}	Proteobacteria class	*Alphaproteobacteria*	42.47 ± 22.25
COM	TP_{30}	Proteobacteria class	*Betaproteobacteria*	34.57 ± 16.15
COM	TP_{30}	Proteobacteria class	*Gammaproteobacteria*	4.47 ± 4.14
COM	TP_{30}	Proteobacteria class	Other	15.37
COM	TP_{30}	Proteobacteria class	Unclassified	3.12 ± 6.24
RES	TP_0	Proteobacteria class	*Alphaproteobacteria*	23.78 ± 18.09
RES	TP_0	Proteobacteria class	*Betaproteobacteria*	46.19 ± 15.11
RES	TP_0	Proteobacteria class	*Gammaproteobacteria*	5.78 ± 1.35
RES	TP_0	Proteobacteria class	Other	23.78
RES	TP_0	Proteobacteria class	Unclassified	0.49 ± 0.15
RES	TP_{30}	Proteobacteria class	*Alphaproteobacteria*	8.95 ± 4.31
RES	TP_{30}	Proteobacteria class	*Betaproteobacteria*	56.98 ± 5.78
RES	TP_{30}	Proteobacteria class	*Gammaproteobacteria*	7.2 ± 1.34
RES	TP_{30}	Proteobacteria class	Other	26.21
RES	TP_{30}	Proteobacteria class	Unclassified	0.66 ± 0.54
COM	TP_0	Family	*Comamonadaceae*	18.96 ± 13.38
COM	TP_0	Family	*Erythrobacteraceae*	11.29 ± 6.75
COM	TP_0	Family	*Flavobacteriaceae*	0.05 ± 0.06
COM	TP_0	Family	*Methylobacteriaceae*	2.39 ± 2.68
COM	TP_0	Family	*Neisseriaceae*	1.06 ± 0.81
COM	TP_0	Family	*Nitrosomonadaceae*	1.28 ± 1.59
COM	TP_0	Family	*Nitrospiraceae*	1.24 ± 2.39
COM	TP_0	Family	*Oxalobacteraceae*	0.96 ± 1.71
COM	TP_0	Family	*Rhizobiales_Incertae_Sedis*	10.26 ± 7.77
COM	TP_0	Family	*Sphingomonadaceae*	13.95 ± 11.11
COM	TP_0	Family	Rare	29.39
COM	TP_0	Family	Unclassified	9.19 ± 13.28
COM	TP_{30}	Family	*Comamonadaceae*	9.38 ± 6.38
COM	TP_{30}	Family	*Erythrobacteraceae*	3.16 ± 3.5
COM	TP_{30}	Family	*Flavobacteriaceae*	1.7 ± 1.68
COM	TP_{30}	Family	*Methylobacteriaceae*	5.57 ± 2.45

COM	TP_{30}	Family	*Neisseriaceae*	12.63 ± 9.95
COM	TP_{30}	Family	*Nitrosomonadaceae*	1.28 ± 1.72
COM	TP_{30}	Family	*Nitrospiraceae*	0.79 ± 0.8
COM	TP_{30}	Family	*Oxalobacteraceae*	4.78 ± 4.54
COM	TP_{30}	Family	*Rhizobiales_Incertae_Sedis*	6.47 ± 11.55
COM	TP_{30}	Family	*Sphingomonadaceae*	12.56 ± 7.25
COM	TP_{30}	Family	Rare	35.17
COM	TP_{30}	Family	Unclassified	6.51 ± 11.01
RES	TP_0	Family	*Comamonadaceae*	6.58 ± 2.44
RES	TP_0	Family	*Erythrobacteraceae*	0 ± 0
RES	TP_0	Family	*Flavobacteriaceae*	4.98 ± 2.05
RES	TP_0	Family	*Methylobacteriaceae*	6.82 ± 6.97
RES	TP_0	Family	*Neisseriaceae*	26.99 ± 10.19
RES	TP_0	Family	*Nitrosomonadaceae*	0.13 ± 0.18
RES	TP_0	Family	*Nitrospiraceae*	0.11 ± 0.03
RES	TP_0	Family	*Oxalobacteraceae*	9.95 ± 3.21
RES	TP_0	Family	*Rhizobiales_Incertae_Sedis*	0.02 ± 0.02
RES	TP_0	Family	*Sphingomonadaceae*	11.78 ± 9.1
RES	TP_0	Family	Rare	31.06
RES	TP_0	Family	Unclassified	1.58 ± 0.67
RES	TP_{30}	Family	*Comamonadaceae*	8.06 ± 1.29
RES	TP_{30}	Family	*Erythrobacteraceae*	0 ± 0
RES	TP_{30}	Family	*Flavobacteriaceae*	7.32 ± 0.51
RES	TP_{30}	Family	*Methylobacteriaceae*	2.12 ± 1.68
RES	TP_{30}	Family	*Neisseriaceae*	35.13 ± 5.04
RES	TP_{30}	Family	*Nitrosomonadaceae*	0.01 ± 0.02
RES	TP_{30}	Family	*Nitrospiraceae*	0.16 ± 0.06
RES	TP_{30}	Family	*Oxalobacteraceae*	12.32 ± 1.22
RES	TP_{30}	Family	*Rhizobiales_Incertae_Sedis*	0.03 ± 0.04
RES	TP_{30}	Family	*Sphingomonadaceae*	2.33 ± 1.94
RES	TP_{30}	Family	Rare	31.06
RES	TP_{30}	Family	Unclassified	1.47 ± 0.9
COM	TP_0	Genus	*Hydrogenophaga*	3.41 ± 7.63
COM	TP_0	Genus	*Legionella*	0.01 ± 0.01
COM	TP_0	Genus	*Mycobacterium*	0.21 ± 0.2
COM	TP_0	Genus	*Nitrosomonas*	0.12 ± 0.2
COM	TP_0	Genus	*Nitrospira*	1.24 ± 2.39
COM	TP_0	Genus	*Phreatobacter*	10.26 ± 7.77
COM	TP_0	Genus	*Pseudomonas*	0.08 ± 0.13
COM	TP_0	Genus	*Pseudorhodoferax*	11.62 ± 10.43
COM	TP_0	Genus	*Sphingomonas*	10 ± 8.35
COM	TP_0	Genus	Other	48.45
COM	TP_0	Genus	Unclassified	14.59 ± 16.43

COM	TP_{30}	Genus	*Hydrogenophaga*	2.41 ± 3.61
COM	TP_{30}	Genus	*Legionella*	0.24 ± 0.2
COM	TP_{30}	Genus	*Mycobacterium*	0.41 ± 0.48
COM	TP_{30}	Genus	*Nitrosomonas*	0.57 ± 1.22
COM	TP_{30}	Genus	*Nitrospira*	0.79 ± 0.8
COM	TP_{30}	Genus	*Phreatobacter*	6.47 ± 11.55
COM	TP_{30}	Genus	*Pseudomonas*	2.98 ± 2.74
COM	TP_{30}	Genus	*Pseudorhodoferax*	3.2 ± 3.5
COM	TP_{30}	Genus	*Sphingomonas*	11.23 ± 7.06
COM	TP_{30}	Genus	Other	44.97
COM	TP_{30}	Genus	Unclassified	26.73 ± 17.55
RES	TP_0	Genus	*Hydrogenophaga*	0.06 ± 0.03
RES	TP_0	Genus	*Legionella*	0.38 ± 0.22
RES	TP_0	Genus	*Mycobacterium*	3.52 ± 6.59
RES	TP_0	Genus	*Nitrosomonas*	0.13 ± 0.18
RES	TP_0	Genus	*Nitrospira*	0.11 ± 0.03
RES	TP_0	Genus	*Phreatobacter*	0.02 ± 0.02
RES	TP_0	Genus	*Pseudomonas*	3.34 ± 1.21
RES	TP_0	Genus	*Pseudorhodoferax*	0.01 ± 0.02
RES	TP_0	Genus	*Sphingomonas*	5.33 ± 3.61
RES	TP_0	Genus	Other	58.34
RES	TP_0	Genus	Unclassified	28.77 ± 9.75
RES	TP_{30}	Genus	*Hydrogenophaga*	0.06 ± 0.03
RES	TP_{30}	Genus	*Legionella*	0.48 ± 0.1
RES	TP_{30}	Genus	*Mycobacterium*	0.1 ± 0.04
RES	TP_{30}	Genus	*Nitrosomonas*	0.01 ± 0.02
RES	TP_{30}	Genus	*Nitrospira*	0.16 ± 0.06
RES	TP_{30}	Genus	*Phreatobacter*	0.03 ± 0.04
RES	TP_{30}	Genus	*Pseudomonas*	4.76 ± 1.14
RES	TP_{30}	Genus	*Pseudorhodoferax*	0.01 ± 0.02
RES	TP_{30}	Genus	*Sphingomonas*	2.12 ± 1.65
RES	TP_{30}	Genus	Other	55.32
RES	TP_{30}	Genus	Unclassified	36.95 ± 5.13

Abbreviation: COM, commercial building; RES, residential household.

Table S6: Percent sequencing identity shared between reference 16S rRNA strains of Nitrosomonas, Nitrospira, Mycobacterium, Legionella, and Pseudomonas of selected ASVs as determined from NCBI.

**Genus**	**ASV**	**Taxonomy**	**Percent identity**
Nitrosomonas	ASV51	*Nitrosomonas oligotropha*	98.77
Nitrosomonas	ASV295	*Nitrosomonas oligotropha*	98.96
Nitrosomonas	ASV1744	*Nitrosomonas oligotropha*	99.11

Nitrosomonas	ASV165	*Nitrosomonas ureae*	98.9
Nitrosomonas	ASV247	*Nitrosomonas ureae*	98.36
Nitrosomonas	ASV1990	*Nitrosomonas ureae*	99.59
Nitrospira	ASV162	*Nitrospira lenta*	99.86
Nitrospira	ASV82	*Nitrospira lenta*	99.86
Nitrospira	ASV353	*Nitrospira lenta*	99.93
Legionella	ASV789	*Legionella birminghamensis*	94.62
Legionella	ASV1052	*Legionella massiliensis*	99.93
Legionella	ASV1524	*Legionella beliardensis*	98.62
Legionella	ASV1537	*Legionella birminghamensis*	94.68
Legionella	ASV1859	*Legionella birminghamensis*	94.61
Legionella	ASV1941	*Legionella parisiensis*	97.95
Pseudomonas	ASV15	*Pseudomonas neuropathica*	100
Pseudomonas	ASV99	*Pseudomonas neuropathica*	99.93
Pseudomonas	ASV223	*Pseudomonas monsensis*	100
Pseudomonas	ASV233	*Pseudomonas thivervalensis*	100
Pseudomonas	ASV570	*Pseudomonas veronii*	99.93
Pseudomonas	ASV888	*Pseudomonas veronii*	99.93
Pseudomonas	ASV903	*Pseudomonas veronii*	99.86
Pseudomonas	ASV979	*Pseudomonas congelans*	100
Pseudomonas	ASV1068	*Pseudomonas fluorescens*	100
Pseudomonas	ASV1207	*Pseudomonas veronii*	99.93
Pseudomonas	ASV1417	*Pseudomonas neuropathica*	99.93
Pseudomonas	ASV1669	*Pseudomonas monsensis*	99.93
Pseudomonas	ASV1866	*Pseudomonas psychrophila*	99.86
Pseudomonas	ASV1965	*Pseudomonas synxantha*	99.79
Pseudomonas	ASV2035	*Pseudomonas thivervalensis*	99.79
Pseudomonas	ASV121	*Pseudomonas peli*	100
Mycobacterium	ASV455	*Mycobacterium gordonae*	99.86
Mycobacterium	ASV1168	*Mycobacterium gordonae*	99.66
Mycobacterium	ASV465	*Mycobacterium gordonae*	100
Mycobacterium	ASV1418	*Mycobacterium lentiflavum*	100
Mycobacterium	ASV687	*Mycobacterium lentiflavum*	100
Mycobacterium	ASV832	*Mycobacterium persicum*	99.93
Mycobacterium	ASV987	*Mycobacterium iranicum*	100
Mycobacterium	ASV1355	*Mycobacterium neglectum*	99.79
Mycobacterium	ASV340	*Mycobacterium tusciae*	99.52
Mycobacterium	ASV201	*Mycobacterium frederiksbergense*	99.79
Mycobacterium	ASV58	*Mycobacterium frederiksbergense*	99.93
Mycobacterium	ASV63	*Mycobacterium frederiksbergense*	99.86

Table S7: Absolute abundances of Mycobacterium ASVs that were detected in the TP_0 and TP_{30} samples of the commercial buildings and residential households the first month of building reopening. Absolute abundances were estimated by multiplying the relative abundance of ASVs with the total cell count obtained using flow cytometry.

**Site type**	**Site***	**Time point**	**ASV**	**Absolute abundance (cells.mL^{-1})**	**Related *Mycobacterium* species**	**Slow or fast grower**
RES	RES1	TP_0	ASV58	1.65E+03	*Mycobacterium frederiksbergense*	Rapid grower
RES	RES3	TP_0	ASV58	1.01E+04	*Mycobacterium frederiksbergense*	Rapid grower
RES	RES3	TP_{30}	ASV58	3.45E+02	*Mycobacterium frederiksbergense*	Rapid grower
RES	RES4	TP_0	ASV58	1.62E+05	*Mycobacterium frederiksbergense*	Rapid grower
COM	COM1.1	TP_{30}	ASV58	4.20E+04	*Mycobacterium frederiksbergense*	Rapid grower
COM	COM1.2	TP_{30}	ASV58	9.90E+03	*Mycobacterium frederiksbergense*	Rapid grower
COM	COM2.1	TP_{30}	ASV58	3.07E+03	*Mycobacterium frederiksbergense*	Rapid grower
COM	COM2.2	TP_0	ASV58	5.94E+04	*Mycobacterium frederiksbergense*	Rapid grower
COM	COM2.2	TP_{30}	ASV58	1.30E+04	*Mycobacterium frederiksbergense*	Rapid grower
RES	RES4	TP_0	ASV63	1.58E+05	*Mycobacterium frederiksbergense*	Rapid grower
COM	COM2.2	TP_{30}	ASV63	1.14E+04	*Mycobacterium frederiksbergense*	Rapid grower
RES	RES1	TP_0	ASV201	4.13E+02	*Mycobacterium frederiksbergense*	Rapid grower
RES	RES1	TP_{30}	ASV201	1.11E+03	*Mycobacterium frederiksbergense*	Rapid grower
COM	COM2.1	TP_{30}	ASV201	1.84E+03	*Mycobacterium frederiksbergense*	Rapid grower
COM	COM2.2	TP_{30}	ASV201	1.09E+04	*Mycobacterium frederiksbergense*	Rapid grower
RES	RES4	TP_0	ASV340	2.18E+03	*Mycobacterium tusciae*	Slow grower
COM	COM3.2	TP_0	ASV340	9.86E+04	*Mycobacterium tusciae*	Slow grower
COM	COM3.2	TP_{30}	ASV340	4.89E+02	*Mycobacterium tusciae*	Slow grower
COM	COM2.2	TP_0	ASV455	2.70E+04	*Mycobacterium gordonae*	Slow grower
COM	COM2.2	TP_{30}	ASV455	2.38E+04	*Mycobacterium gordonae*	Slow grower
RES	RES3	TP_0	ASV465	3.60E+02	*Mycobacterium gordonae*	Slow grower
COM	COM2.1	TP_0	ASV465	1.37E+05	*Mycobacterium gordonae*	Slow grower
COM	COM2.1	TP_{30}	ASV465	4.30E+03	*Mycobacterium gordonae*	Slow grower
RES	RES1	TP_0	ASV623	8.27E+02	Unclassified	Unknown

RES	RES2	TP_0	ASV623	2.65E+02	Unclassified	Unknown
RES	RES2	TP_{30}	ASV623	6.19E+02	Unclassified	Unknown
RES	RES4	TP_0	ASV623	1.45E+03	Unclassified	Unknown
RES	RES4	TP_{30}	ASV623	7.83E+02	Unclassified	Unknown
COM	COM1.2	TP_{30}	ASV623	1.29E+03	Unclassified	Unknown
COM	COM2.1	TP_{30}	ASV623	1.84E+03	Unclassified	Unknown
COM	COM3.2	TP_{30}	ASV623	9.79E+02	Unclassified	Unknown
RES	RES3	TP_0	ASV666	7.20E+02	Unclassified	Unknown
RES	RES2	TP_0	ASV673	2.65E+02	Unclassified	Unknown
RES	RES3	TP_0	ASV673	1.08E+03	Unclassified	Unknown
RES	RES3	TP_{30}	ASV673	5.76E+02	Unclassified	Unknown
COM	COM1.1	TP_{30}	ASV673	5.25E+03	Unclassified	Unknown
COM	COM3.1	TP_{30}	ASV673	1.66E+03	Unclassified	Unknown
COM	COM2.1	TP_0	ASV687	2.93E+04	*Mycobacterium lentiflavum*	Slow grower
COM	COM2.1	TP_{30}	ASV832	1.11E+04	*Mycobacterium persicum*	Slow grower
COM	COM2.2	TP_{30}	ASV987	9.85E+03	*Mycobacterium iranicum*	Rapid grower
COM	COM2.1	TP_{30}	ASV1168	1.84E+03	*Mycobacterium gordonae*	Slow grower
RES	RES2	TP_{30}	ASV1355	2.06E+02	*Mycobacterium neglectum*	Rapid grower
RES	RES1	TP_{30}	ASV1407	4.94E+02	Unclassified	Unknown
COM	COM2.1	TP_0	ASV1418	1.95E+04	*Mycobacterium lentiflavum*	Slow grower
COM	COM2.1	TP_{30}	ASV1418	1.84E+03	*Mycobacterium lentiflavum*	Slow grower
COM	COM2.1	TP_0	ASV1492	3.90E+04	Unclassified	Unknown
COM	COM1.2	TP_{30}	ASV1649	8.61E+02	Unclassified	Unknown

Abbreviation: COM, commercial building; RES, residential household.
*Naming convention encompass (i) site type, (ii) site, (iii) time point, and (iv) month. See Example 1 and Example 2 below.
Example 1: COM3.2.1.6 (i) Commercial building (ii) site 3.2, (iii) time point 1, (iv) month 6
Example 2: RES4.7.6 (i) Residential household (ii) site 4, (iii) time point 7, (iv) month 6

Table S8: Distance-based redundancy analysis and variation partitioning analysis testing the relationship between water chemistry parameters and microbial community composition associated with TP0 and TP30 samples of the commercial building and residential household sites that were collected the first month of building reopening using Bray-Curtis distances on flow cytometric fingerprinting data and 16S rRNA sequencing data.

A. Colinear water chemistry parameters identified prior to dbRDA, with "POS" and "NEG" denoting positive and negative correlations at Pearson's correlation coefficients of > 0.70 and < 0.70, respectively.

**Colinear water chemistry parameter**	**Cluster**	**Selected water chemistry parameter**	**Pearson R2**	**p-value code**
Copper vs. Total chlorine	Total chlorine [Copper, NEG]	Total chlorine	-0.760	***
Manganese vs Copper	Manganese [Copper, POS] [Magnesium, POS]	Manganese	0.704	***
Manganese vs Magnesium	Manganese [Copper, POS] [Magnesium, POS]	Manganese	0.711	***
Iron vs Nitrite	Iron [nitrite, POS]	Iron	0.855	***
TN vs. Lead	TN [pH, POS] [TOC, POS] [Lead, NEG]	TN	0.707	***
TN vs pH	TN [pH, POS] [TOC, POS] [Lead, NEG]	TN	-0.707	***
TN vs TOC	TN [pH, POS] [TOC, POS] [Lead, NEG]	TN	-0.720	***

Abbreviation: TN, total nitrogen; TOC, total organic carbon; DO, dissolved oxygen
Significance codes: '***' 0.001 '**' 0.01 '*' 0.05 '.' 0.1 '' 1

B. Distance-based redundancy analysis and variation partitioning analysis using flow cytometric fingerprinting data.

Permutation test for dbrda under reduced model
Terms added sequentially (first to last)
Permutation: free
Number of permutations: 999
Model: dbrda(formula = fbasis.m1.tp1.tp7.mat ~ Temperature + Conductivity + Total chlorine + Ammonium + Nitrate + Iron + Manganese + Zinc + TN, data = meta.data.m1.tp1.tp7, distance = "bray")

**Water chemistry parameter**	**Degrees of freedom**	**Sum of squares**	**F statistic**	**p-value**	**p-value code**
Temperature	1	0.104	2.661	0.020	*
Conductivity	1	0.055	1.404	0.195	
Total chlorine [Copper, NEG]	1	0.107	2.748	0.011	*
Ammonium	1	0.069	1.759	0.103	
Nitrate	1	0.031	0.803	0.567	
Iron [nitrite, POS]	1	0.033	0.840	0.535	
Manganese [Copper, POS] [Magnesium, POS]	1	0.099	2.532	0.023	*

Zinc	1	0.043	1.116	0.339	
TN [pH, POS] [TOC, POS] [Lead, NEG]	1	0.039	1.006	0.448	
Residual	10	0.389			

Abbreviation: TN, total nitrogen; TOC, total organic carbon; DO, dissolved oxygen

Significance codes: ‘***’ 0.001 ‘**’ 0.01 ‘*’ 0.05 ‘.’ 0.1 ‘’ 1
p-values based on more than 999 permutations

Variance partition analysis:

Call: varpart(Y = fbasis.m1.tp1.tp7.mat, X = ~Site type, ~Total chlorine, ~Temperature, ~Manganese, data= meta.data.m1.tp1.tp7)
Explanatory tables:
X1: ~Site type
X2: ~Total chlorine [Copper, NEG]
X3: ~Temperature
X4: ~Manganese [Copper, POS] [Magnesium, POS]

No. of explanatory tables: 4
Total variation (SS): 10503873
Variance: 552835
No. of observations: 20

**Partition table**	**Degrees of freedom**	**R2**	**Adjusted R2**	**Testable**
[aeghklno] = X1	1	0.159	0.112	TRUE
[befiklmo] = X2	1	0.163	0.116	TRUE
[cfgjlmno] = X3	1	0.084	0.033	TRUE
[dhijkmno] = X4	1	0.105	0.055	TRUE
[abefghiklmno] = X1+X2	2	0.217	0.125	TRUE
[acefghjklmno] = X1+X3	2	0.197	0.102	TRUE
[adeghijklmno] = X1+X4	2	0.195	0.100	TRUE
[bcefgijklmno] = X2+X3	2	0.191	0.096	TRUE
[bdefhijklmno] = X2+X4	2	0.212	0.120	TRUE
[cdfghijklmno] = X3+X4	2	0.171	0.074	TRUE
[abcefghijklmno] = X1+X2+X3	3	0.245	0.104	TRUE
[abdefghijklmno] = X1+X2+X4	3	0.253	0.113	TRUE
[acdefghijklmno] = X1+X3+X4	3	0.231	0.087	TRUE
[bcdefghijklmno] = X2+X3+X4	3	0.238	0.096	TRUE
[abcdefghijklmno] = All	4	0.278	0.085	TRUE

**Individual fractions**	**Degrees of freedom**	**R2**	**Adjusted R2**	**Testable**
[a] = X1 | X2+X3+X4	1		-0.010	TRUE
[b] = X2 | X1+X3+X4	1		-0.002	TRUE
[c] = X3 | X1+X2+X4	1		-0.027	TRUE

[d] = X4 | X1+X2+X3	1		-0.019	TRUE
[e]	0		0.024	FALSE
[f]	0		0.014	FALSE
[g]	0		0.003	FALSE
[h]	0		0.019	FALSE
[i]	0		0.003	FALSE
[j]	0		0.006	FALSE
[k]	0		0.038	FALSE
[l]	0		0.028	FALSE
[m]	0		-0.003	FALSE
[n]	0		-0.003	FALSE
[o]	0		0.014	FALSE
[p] = Residuals	0		0.915	FALSE

C. Distance-based redundancy analysis and variation partitioning analysis using 16S rRNA sequencing data.

Permutation test for dbrda under reduced model
Terms added sequentially (first to last)
Permutation: free
Number of permutations: 999
Model: dbrda(formula = fbasis.m1.tp1.tp7.mat ~ Temperature + Conductivity + Total chlorine + Ammonium + Nitrate + Iron + Manganese + Zinc + TN, data = meta.data.m1.tp1.tp7, distance = "bray")

Water chemistry parameter	Degrees of freedom	Sum of squares	F statistic	p-value	p-value code
Temperature	1	0.104	2.661	0.020	*
Conductivity	1	0.055	1.404	0.195	
Total chlorine [Copper, NEG]	1	0.107	2.748	0.011	*
Ammonium	1	0.069	1.759	0.103	
Nitrate	1	0.031	0.803	0.567	
Iron [nitrite, POS]	1	0.033	0.840	0.535	
Manganese [Copper, POS] [Magnesium, POS]	1	0.099	2.532	0.023	*
Zinc	1	0.043	1.116	0.339	
TN [pH, POS] [TOC, POS] [Lead, NEG]	1	0.039	1.006	0.448	
Residual	10	0.389	NA	NA	

Abbreviation: TN, total nitrogen; TOC, total organic carbon; DO, dissolved oxygen
Significance codes: ‘***’ 0.001 ‘**’ 0.01 ‘*’ 0.05 ‘.’ 0.1 ‘’ 1
p-values based on more than 999 permutations

Variance partition analysis:

Call: varpart(Y = fbasis.m1.tp1.tp7.mat, X = ~Site type, ~Total chlorine, ~Temperature, ~Manganese, data= meta.data.m1.tp1.tp7)
Explanatory tables:
X1: ~Site type
X2: ~Total chlorine [Copper, NEG]
X3: ~Temperature
X4: ~Manganese [Copper, POS] [Magnesium, POS]

No. of explanatory tables: 4
Total variation (SS): 10503873
Variance: 552835
No. of observations: 20

**Partition table**	**Degrees of freedom**	**R2**	**Adjusted R2**	**Testable**
[aeghklno] = X1	1	0.176	0.127	TRUE
[befiklmo] = X2	1	0.190	0.143	TRUE
[cfgjlmno] = X3	1	0.108	0.056	TRUE
[dhijkmno] = X4	1	0.160	0.110	TRUE
[abefghiklmno] = X1+X2	2	0.244	0.149	TRUE
[acefghjklmno] = X1+X3	2	0.226	0.129	TRUE
[adeghijklmno] = X1+X4	2	0.321	0.236	TRUE
[bcefgijklmno] = X2+X3	2	0.229	0.132	TRUE
[bdefhijklmno] = X2+X4	2	0.308	0.221	TRUE
[cdfghijklmno] = X3+X4	2	0.253	0.160	TRUE
[abcefghijklmno] = X1+X2+X3	3	0.282	0.138	TRUE
[abdefghijklmno] = X1+X2+X4	3	0.390	0.268	TRUE
[acdefghijklmno] = X1+X3+X4	3	0.367	0.241	TRUE
[bcdefghijklmno] = X2+X3+X4	3	0.344	0.212	TRUE
[abcdefghijklmno] = All	4	0.423	0.258	TRUE

**Individual fractions**	**Degrees of freedom**	**R2**	**Adjusted R2**	**Testable**
[a] = X1 | X2+X3+X4	1		0.046	TRUE
[b] = X2 | X1+X3+X4	1		0.018	TRUE
[c] = X3 | X1+X2+X4	1		-0.010	TRUE
[d] = X4 | X1+X2+X3	1		0.120	TRUE
[e]	0		0.035	FALSE
[f]	0		0.014	FALSE
[g]	0		0.001	FALSE
[h]	0		-0.040	FALSE
[i]	0		-0.008	FALSE
[j]	0		-0.001	FALSE
[k]	0		0.032	FALSE

[l]	0		0.044	FALSE
[m]	0		-0.002	FALSE
[n]	0		0.000	FALSE
[o]	0		0.009	FALSE
[p] = Residuals	0		0.915	FALSE

Table S9: Descriptive statistics and independent t-test (Mann-Whitney U test) to compare the means (or medians) of cytometric measures associated with first draw (TP_0) and all flushed samples (TP_{5-30}) of commercial building and residential household sites grouped by month. Measures include total cell concentrations, intact cell proportions, phenotypic diversity index and evenness.

Site type	Month	Parameter	First draw sample (TP_0, M ± SD, median)	All flushed samples (TP_{5-30}, M ± SD, median)	Shapiro–Wilk test	Independent t-test or Mann-Whitney U test
COM	Jun	TCC (cells.mL^{-1})	807135 ± 391315, 916437	209698 ± 288664, 67395	W = 0.75898, p-value = 6.586e-07	W = 196, p-value = 0.0005768
COM	Jul	TCC (cells.mL^{-1})	604449 ± 663578, 366157	148664 ± 276955, 45363	W = 0.54314, p-value = 3.069e-10	W = 178, p-value = 0.009697
COM	Aug	TCC (cells.mL^{-1})	704643 ± 910133, 390815	48077 ± 41241, 33177	W = 0.32217, p-value = 1.113e-12	W = 162, p-value = 0.05288
COM	Sep	TCC (cells.mL^{-1})	346849 ± 432540, 140386	15414 ± 9723, 13956	W = 0.30171, p-value = 7.09e-13	W = 164, p-value = 0.04401
COM	Oct	TCC (cells.mL^{-1})	329077 ± 396745, 166979	7061 ± 5250, 3975	W = 0.3002, p-value = 6.861e-13	W = 194, p-value = 0.0008521
COM	Nov	TCC (cells.mL^{-1})	280311 ± 432973, 49897	18905 ± 13473, 17777	W = 0.27563, p-value = 4.043e-13	W = 166, p-value = 0.03636
COM	Jun	Intact cell proportion (%)	52.98 ± 3.42, 53.28	25.86 ± 19.31, 26.67	W = 0.86617, p-value = 0.0001619	W = 196, p-value = 0.0005768
COM	Jul	Intact cell proportion (%)	35.07 ± 13.98, 36.44	17.03 ± 14.03, 10.43	W = 0.85439, p-value = 8.051e-05	W = 182, p-value = 0.005765
COM	Aug	Intact cell proportion (%)	28.6 ± 18.63, 27.48	12.45 ± 10.54, 8.4	W = 0.82131, p-value = 1.302e-05	W = 167, p-value = 0.03295
COM	Sep	Intact cell proportion (%)	30.09 ± 15.72, 27.18	18.64 ± 9.76, 16.46	W = 0.85364, p-value = 7.705e-05	W = 166, p-value = 0.03636
COM	Oct	Intact cell proportion (%)	17.82 ± 12.92, 15.31	12.14 ± 6.29, 12.26	W = 0.89671, p-value = 0.001149	W = 138, p-value = 0.2972
COM	Nov	Intact cell proportion (%)	21.8 ± 14.37, 23.69	9.37 ± 5.67, 7.98	W = 0.8254, p-value = 1.614e-05	W = 156, p-value = 0.08807
COM	Jun	Phenotypic diversity index	1938 ± 163, 1947	2157 ± 365, 2198	W = 0.96609, p-value = 0.2427	t = -1.4319, df = 40, p-value = 0.16
COM	Jul	Phenotypic diversity index	2068 ± 506, 2060	2423 ± 459, 2546	W = 0.94495, p-value = 0.04267	W = 68, p-value = 0.1595
COM	Aug	Phenotypic diversity index	1987 ± 644, 1846	2612 ± 302, 2702	W = 0.87222, p-value = 0.0002346	W = 47, p-value = 0.02691
COM	Sep	Phenotypic diversity index	2136 ± 560, 1877	2640 ± 491, 2757	W = 0.90914, p-value = 0.002735	W = 52, p-value = 0.04401

COM	Oct	Phenotypic diversity index	2211 ± 541, 2391	2793 ± 253, 2825	W = 0.83837, p-value = 3.251e-05	W = 30, p-value = 0.003259
COM	Nov	Phenotypic diversity index	2219 ± 489, 2484	2707 ± 312, 2621	W = 0.92099, p-value = 0.006512	W = 53, p-value = 0.04828
COM	Jun	Evenness	0.21 ± 0.02, 0.2	0.24 ± 0.02, 0.24	W = 0.97149, p-value = 0.3701	t = -3.261, df = 40, p-value = 0.002273
COM	Jul	Evenness	0.22 ± 0.03, 0.22	0.23 ± 0.02, 0.23	W = 0.96988, p-value = 0.3272	t = -1.4886, df = 40, p-value = 0.1444
COM	Aug	Evenness	0.22 ± 0.03, 0.22	0.24 ± 0.02, 0.25	W = 0.86424, p-value = 0.0001441	W = 50, p-value = 0.03636
COM	Sep	Evenness	0.24 ± 0.05, 0.24	0.28 ± 0.03, 0.28	W = 0.9809, p-value = 0.6963	t = -2.5538, df = 40, p-value = 0.01457
COM	Oct	Evenness	0.24 ± 0.04, 0.25	0.31 ± 0.03, 0.32	W = 0.90246, p-value = 0.001707	W = 20, p-value = 0.0005768
COM	Nov	Evenness	0.24 ± 0.04, 0.24	0.27 ± 0.03, 0.27	W = 0.98733, p-value = 0.9161	W = 53, p-value = 0.04828
RES	Jun	TCC (cells.mL^{-1})	20695 ± 12036, 18979	9437 ± 1625, 8718	W = 0.55056, p-value = 3.948e-08	W = 85, p-value = 0.01182
RES	Jul	TCC (cells.mL^{-1})	19049 ± 4608, 19423	16600 ± 1359, 16284	W = 0.81014, p-value = 0.0001619	W = 64, p-value = 0.3224
RES	Aug	TCC (cells.mL^{-1})	17627 ± 2465, 17373	18581 ± 1912, 18672	W = 0.94813, p-value = 0.1778	t = -0.73823, df = 3.6274, p-value = 0.5053
RES	Sep	TCC (cells.mL^{-1})	7644 ± 3442, 7013	4735 ± 1577, 4972	W = 0.89216, p-value = 0.007534	W = 78, p-value = 0.05031
RES	Oct	TCC (cells.mL^{-1})	3777 ± 1033, 4058	3603 ± 1161, 3372	W = 0.92964, p-value = 0.06038	t = 0.30736, df = 4.3705, p-value = 0.7727
RES	Nov	TCC (cells.mL^{-1})	4835 ± 1194, 4478	4951 ± 681, 4844	W = 0.94646, p-value = 0.1613	t = -0.18825, df = 3.3334, p-value = 0.8616
RES	Jun	Intact cell proportion (%)	17.06 ± 12.68, 19.4	1.37 ± 0.76, 1.18	W = 0.43754, p-value = 2.749e-09	W = 90, p-value = 0.002637
RES	Jul	Intact cell proportion (%)	9.05 ± 6.83, 8.07	2.98 ± 1.56, 2.73	W = 0.67674, p-value = 1.359e-06	W = 78, p-value = 0.05031
RES	Aug	Intact cell proportion (%)	3.17 ± 1.25, 3.22	3.56 ± 1.43, 3.6	W = 0.94115, p-value = 0.1182	t = -0.565, df = 4.4162, p-value = 0.5996
RES	Sep	Intact cell proportion (%)	15.05 ± 2.5, 14.76	17.82 ± 7.27, 15.62	W = 0.9322, p-value = 0.07006	W = 40, p-value = 0.635
RES	Oct	Intact cell proportion (%)	13.15 ± 5.49, 12.16	9.79 ± 2.98, 9.54	W = 0.91359, p-value = 0.02415	W = 69, p-value = 0.1857

RES	Nov	Intact cell proportion (%)	4.03 ± 3.31, 3.27	2.67 ± 1.03, 2.57	W = 0.85836, p-value = 0.001385	W = 54, p-value = 0.7279
RES	Jun	Phenotypic diversity index	2931 ± 610, 3002	2900 ± 348, 2801	W = 0.93556, p-value = 0.08525	t = 0.096676, df = 3.3339, p-value = 0.9285
RES	Jul	Phenotypic diversity index	2982 ± 225, 3015	2936 ± 176, 2945	W = 0.96968, p-value = 0.572	t = 0.38793, df = 3.6347, p-value = 0.7197
RES	Aug	Phenotypic diversity index	2819 ± 83, 2827	2796 ± 78, 2794	W = 0.989, p-value = 0.9884	t = 0.52923, df = 3.9535, p-value = 0.625
RES	Sep	Phenotypic diversity index	3283 ± 341, 3145	3019 ± 256, 3074	W = 0.95267, p-value = 0.231	t = 1.4804, df = 3.5877, p-value = 0.2207
RES	Oct	Phenotypic diversity index	2687 ± 323, 2725	2771 ± 165, 2727	W = 0.9885, p-value = 0.9853	t = -0.51052, df = 3.2634, p-value = 0.6422
RES	Nov	Phenotypic diversity index	3194 ± 208, 3111	3211 ± 195, 3199	W = 0.97533, p-value = 0.7277	t = -0.15141, df = 3.9382, p-value = 0.8871
RES	Jun	Evenness	0.28 ± 0.03, 0.28	0.29 ± 0.01, 0.29	W = 0.93022, p-value = 0.06243	t = -0.78645, df = 3.1197, p-value = 0.487
RES	Jul	Evenness	0.27 ± 0.01, 0.27	0.27 ± 0.01, 0.28	W = 0.98699, p-value = 0.9728	t = -1.5597, df = 3.85, p-value = 0.1966
RES	Aug	Evenness	0.26 ± 0.01, 0.26	0.26 ± 0.01, 0.26	W = 0.96352, p-value = 0.421	t = 0.15937, df = 3.1628, p-value = 0.883
RES	Sep	Evenness	0.32 ± 0.03, 0.31	0.33 ± 0.02, 0.33	W = 0.96031, p-value = 0.3546	t = -0.349, df = 3.5541, p-value = 0.7468
RES	Oct	Evenness	0.33 ± 0.01, 0.33	0.33 ± 0.01, 0.33	W = 0.94901, p-value = 0.187	t = -0.46313, df = 3.5512, p-value = 0.6702
RES	Nov	Evenness	0.34 ± 0.02, 0.34	0.32 ± 0.01, 0.33	W = 0.90979, p-value = 0.01954	W = 80, p-value = 0.03507

Abbreviation: COM, commercial building; RES, residential household; Jun, June; Jul, July; Aug, August; Sep, September; Oct, October; Nov, November.

Table S10: Descriptive statistics (mean and standard deviation) of total cell concentration and temperature measures that were used to construct flush profiles of the commercial building and residential household sites. The measures were averaged for individual time points (TP_0, 0 min; TP_5, 5 min; TP_{10}, 10 min; TP_{15}, 15 min; TP_{20}, 20 min; TP_{25}, 25 min; and TP_{30}, 30 min) within each month across all commercial building and residential household sites, respectively.

**Site**	**Month**	**Time point**	**TCC (cells.ml^{-1}, M ± SD)**	**Temperature (°C, M ± SD)**	**Flushed volume (L, M ± SD)**
COM	Jun	TP_0	807000 ± 391000	22.55 ± 1.18	0 ± 0
COM	Jun	TP_5	334000 ± 439000	22.67 ± 1.84	17.61 ± 5.24

COM	Jun	TP_{10}	252543 ± 342000	22.8 ± 1.83	35.22 ± 10.47
COM	Jun	TP_{15}	273458 ± 373000	22.1 ± 1.49	52.83 ± 15.71
COM	Jun	TP_{20}	174752 ± 238000	21.48 ± 1.19	70.43 ± 20.95
COM	Jun	TP_{25}	123722 ± 135000	21.58 ± 1.25	88.04 ± 26.19
COM	Jun	TP_{30}	100172 ± 94700	22.57 ± 1.57	105.65 ± 31.42
COM	Jul	TP_{0}	604000 ± 664000	22.95 ± 2.79	0 ± 0
COM	Jul	TP_{5}	305000 ± 524000	23.35 ± 3.1	19.19 ± 6.89
COM	Jul	TP_{10}	246643 ± 409000	21.32 ± 4.25	38.37 ± 13.79
COM	Jul	TP_{15}	126377 ± 136000	21.58 ± 1.59	57.56 ± 20.68
COM	Jul	TP_{20}	80520 ± 71400	22.23 ± 1.11	76.74 ± 27.58
COM	Jul	TP_{25}	70321 ± 62900	22.7 ± 1.94	95.93 ± 34.47
COM	Jul	TP_{30}	63291 ± 64300	22.72 ± 3.46	115.12 ± 41.37
COM	Aug	TP_{0}	705000 ± 910000	22.32 ± 4.11	0 ± 0
COM	Aug	TP_{5}	81600 ± 84700	20.87 ± 4.13	19.5 ± 8.87
COM	Aug	TP_{10}	49693 ± 32900	21.5 ± 2.45	39 ± 17.74
COM	Aug	TP_{15}	37378 ± 16100	21.87 ± 1.46	58.5 ± 26.62
COM	Aug	TP_{20}	39826 ± 23700	22.73 ± 1.8	78 ± 35.49
COM	Aug	TP_{25}	41934 ± 27100	22.93 ± 3.52	97.5 ± 44.36
COM	Aug	TP_{30}	38012 ± 18800	22.23 ± 4.21	117 ± 53.23
COM	Sep	TP_{0}	347000 ± 433000	20.68 ± 4.04	0 ± 0
COM	Sep	TP_{5}	24800 ± 19000	21.22 ± 2.12	19.68 ± 7.24
COM	Sep	TP_{10}	14793 ± 7410	21.97 ± 1.39	39.35 ± 14.48
COM	Sep	TP_{15}	13272 ± 5760	22.27 ± 1.61	59.03 ± 21.72
COM	Sep	TP_{20}	12855 ± 5770	22.93 ± 3.33	78.7 ± 28.96
COM	Sep	TP_{25}	12569 ± 4420	22.3 ± 4.19	98.38 ± 36.2
COM	Sep	TP_{30}	14204 ± 5540	20.7 ± 4.01	118.05 ± 43.44
COM	Oct	TP_{0}	329000 ± 397000	20.55 ± 1.68	0 ± 0
COM	Oct	TP_{5}	9410 ± 8780	21.97 ± 1.47	26.04 ± 10.27
COM	Oct	TP_{10}	6334 ± 5220	21.9 ± 1.81	52.07 ± 20.55
COM	Oct	TP_{15}	5500 ± 4040	22.37 ± 3.38	78.11 ± 30.82
COM	Oct	TP_{20}	5707 ± 4540	22.02 ± 4.14	104.15 ± 41.1

COM	Oct	TP_{25}	5096 ± 3660	20.45 ± 3.91	130.18 ± 51.37
COM	Oct	TP_{30}	10317 ± 2910	20.27 ± 1.69	156.22 ± 61.65
COM	Nov	TP_{0}	280000 ± 433000	21.55 ± 0.98	0 ± 0
COM	Nov	TP_{5}	23400 ± 18600	21.72 ± 1.97	23.8 ± 5.42
COM	Nov	TP_{10}	20308 ± 14100	21.9 ± 3.78	47.6 ± 10.84
COM	Nov	TP_{15}	19489 ± 14900	21.37 ± 4	71.39 ± 16.26
COM	Nov	TP_{20}	19156 ± 16500	20.1 ± 3.74	95.19 ± 21.68
COM	Nov	TP_{25}	16442 ± 9590	20.27 ± 1.88	118.99 ± 27.1
COM	Nov	TP_{30}	14629 ± 9030	21.33 ± 0.86	142.79 ± 32.51
RES	Jun	TP_{0}	20700 ± 12000	23.7 ± 2.17	0 ± 0
RES	Jun	TP_{5}	9410 ± 1130	24.6 ± 1.07	15.01 ± 4.94
RES	Jun	TP_{10}	8870 ± 1140	22.78 ± 1.34	30.03 ± 9.88
RES	Jun	TP_{15}	8760 ± 1850	17.73 ± 2.16	45.04 ± 14.81
RES	Jun	TP_{20}	9240 ± 1760	19.55 ± 1.04	60.05 ± 19.75
RES	Jun	TP_{25}	8530 ± 221	21.3 ± 0.51	75.06 ± 24.69
RES	Jun	TP_{30}	11800 ± 1180	21.13 ± 1.88	90.08 ± 29.63
RES	Jul	TP_{0}	19000 ± 4610	18.38 ± 1.49	0 ± 0
RES	Jul	TP_{5}	17000 ± 2160	15.68 ± 1.29	29.02 ± 12.05
RES	Jul	TP_{10}	16000 ± 867	12.83 ± 0.3	58.05 ± 24.1
RES	Jul	TP_{15}	16700 ± 675	17.38 ± 0.41	87.07 ± 36.14
RES	Jul	TP_{20}	15900 ± 1140	19.58 ± 0.75	116.09 ± 48.19
RES	Jul	TP_{25}	17500 ± 2030	20.75 ± 1.93	145.12 ± 60.24
RES	Jul	TP_{30}	16500 ± 680	18.18 ± 1.67	174.14 ± 72.29
RES	Aug	TP_{0}	17600 ± 2460	15.9 ± 1.01	0 ± 0
RES	Aug	TP_{5}	19900 ± 1510	13.23 ± 0.62	17.99 ± 10.42
RES	Aug	TP_{10}	17900 ± 2590	17.5 ± 0.42	35.98 ± 20.84
RES	Aug	TP_{15}	18400 ± 1510	19.35 ± 0.81	53.97 ± 31.26
RES	Aug	TP_{20}	18400 ± 1820	20.58 ± 1.96	71.96 ± 41.69
RES	Aug	TP_{25}	19500 ± 2310	18.1 ± 1.73	89.94 ± 52.11
RES	Aug	TP_{30}	17400 ± 1470	15.9 ± 1.04	107.93 ± 62.53
RES	Sep	TP_{0}	7640 ± 3440	13.23 ± 0.69	0 ± 0

RES	Sep	TP_5	4530 ± 1700	17.53 ± 0.39	12.15 ± 2.28
RES	Sep	TP_{10}	5110 ± 2280	19.3 ± 0.88	24.3 ± ± 4.57
RES	Sep	TP_{15}	4270 ± 1680	20.55 ± 1.99	36.45 ± 6.85
RES	Sep	TP_{20}	4370 ± 1330	18.15 ± 1.68	48.61 ± 9.13
RES	Sep	TP_{25}	4370 ± 1520	15.9 ± 0.96	60.76 ± 11.41
RES	Sep	TP_{30}	5760 ± 1320	13.28 ± 0.67	72.91 ± 13.7
RES	Oct	TP_0	3780 ± 1030	17.55 ± 0.37	0 ± 0
RES	Oct	TP_5	3820 ± 450	19.25 ± 0.89	23.59 ± 12.39
RES	Oct	TP_{10}	3780 ± 1570	20.48 ± 2.06	47.18 ± 24.77
RES	Oct	TP_{15}	3390 ± 1190	18.15 ± 1.66	70.76 ± 37.16
RES	Oct	TP_{20}	3520 ± 604	15.85 ± 0.89	94.35 ± 49.54
RES	Oct	TP_{25}	3760 ± 2020	13.25 ± 0.59	117.94 ± 61.93
RES	Oct	TP_{30}	3360 ± 1230	17.58 ± 0.35	141.53 ± 74.31
RES	Nov	TP_0	4840 ± 1190	19.28 ± 0.88	0 ± 0
RES	Nov	TP_5	5610 ± 815	20.45 ± 2.04	21.53 ± 13.4
RES	Nov	TP_{10}	4940 ± 559	18.23 ± 1.59	43.07 ± 26.8
RES	Nov	TP_{15}	4680 ± 633	15.85 ± 0.89	64.6 ± 40.2
RES	Nov	TP_{20}	4910 ± 497	13.35 ± 0.61	86.14 ± 53.61
RES	Nov	TP_{25}	4950 ± 893	17.55 ± 0.34	107.67 ± 67.01
RES	Nov	TP_{30}	4620 ± 566	19.2 ± 0.82	129.2 ± 80.41

COM, commercial building; RES, residential household; Jun, June; Jul, July; Aug, August; Sep, September; Oct, October; Nov, November

Table S11: Average Bray-Curtis dissimilarity distances as determined using flow cytometric fingerprint data between first draw samples (TP_0) and all flushed samples ($TP_{5\text{-}30}$) of the commercial building and residential household sites.

**Site type**	**Month**	**Bray-Curtis dissimilarity distance (M ± SD)**
COM	Jun	0.37 ± 0.08
COM	Jul	0.31 ± 0.09
COM	Aug	0.31 ± 0.08
COM	Sep	0.32 ± 0.09
COM	Oct	0.36 ± 0.08

COM	Nov	0.3 ± 0.07
RES	Jun	0.27 ± 0.05
RES	Jul	0.25 ± 0.06
RES	Aug	0.23 ± 0.06
RES	Sep	0.29 ± 0.06
RES	Oct	0.26 ± 0.05
RES	Nov	0.26 ± 0.05

Abbreviation: COM, commercial building; RES, residential household; Jun, June; Jul, July; Aug, August; Sep, September; Oct, October; Nov, November

Table S12: Average Bray-Curtis dissimilarity distances as determined using 16S rRNA gene sequencing data between first draw samples (TP_0) and final first draw samples (TP_{30}) of the commercial building and residential household sites. The explanatory power of sample type (first draw sample vs. final first draw sample) with respect to microbial community composition each month for every site type were assessed using PERMANOVA.

**Site type**	**Month**	**Bray-Curtis dissimilarity distance (M ± SD)**	**PERMANOVA**
COM	Jun	0.62 ± 0.21	F(1,9) = 1.48, R2 = 0.14 p = 0.14
COM	Jul	0.6 ± 0.17	F(1,9) = 1.92, R2 = 0.18 p = 0.08
COM	Aug	0.66 ± 0.18	F(1,9) = 2.63, R2 = 0.23 p = 0.02
COM	Sep	0.61 ± 0.18	F(1,8) = 3.01, R2 = 0.27 p = 0.02
COM	Oct	0.79 ± 0.06	F(1,7) = 5.76, R2 = 0.45 p = 0.01
COM	Nov	0.61 ± 0.2	F(1,7) = 7.35, R2 = 0.39, p = 0.02
RES	Jun	0.41 ± 0.17	F(1,6) = 1.14, R2 = 0.16 p = 0.26
RES	Jul	0.31 ± 0.03	F(1,5) = 0.68, R2 = 0.12 p = 0.69
RES	Aug	0.33 ± 0.02	F(1,6) = 0.60, R2 = 0.09 p = 0.96
RES	Sep	0.38 ± 0.11	F(1,5) = 0.85, R2 = 0.15 p = 0.73
RES	Oct	0.39 ± 0.07	F(1,6) = 1.10, R2 = 0.15 p = 0.37
RES	Nov	0.35 ± 0.14	F(1,4) = 0.90, R2 = 0.18 p = 0.60

Abbreviation: COM, commercial building; RES, residential household; Jun, June; Jul, July; Aug, August; Sep, September; Oct, October; Nov, November

Table S13: Time-decay relationships of commercial building and residential household microbial communities with relation to flush duration. Bray-Curtis dissimilarity distances was calculated on flow cytometric data and used to assess relationships with flush duration expressed in minutes. Regression analysis was performed monthly for each site, resulting in 60 linear regressions. Adjusted R^2 value, slope and significance level are displayed for each linear regression.

Duration (min)	Site type	Site*	Month	Bray-Curtis dissimilarity distance (M ± SD)	Linear regression
5	COM	COM1.1	Jun	0.24 ± 0.11	Adjusted R^2 = 0.18057, Slope = 0.0062863, p = 6.5932e−10
10	COM	COM1.1	Jun	0.27 ± 0.09	
15	COM	COM1.1	Jun	0.31 ± 0.1	
20	COM	COM1.1	Jun	0.33 ± 0.09	
25	COM	COM1.1	Jun	0.37 ± 0.1	
30	COM	COM1.1	Jun	0.38 ± 0.09	
5	COM	COM1.1	Jul	0.2 ± 0.1	Adjusted R^2 = 0.040242, Slope = 0.0026773, p = 0.0032615
10	COM	COM1.1	Jul	0.19 ± 0.09	
15	COM	COM1.1	Jul	0.19 ± 0.08	
20	COM	COM1.1	Jul	0.24 ± 0.09	
25	COM	COM1.1	Jul	0.24 ± 0.09	
30	COM	COM1.1	Jul	0.27 ± 0.06	
5	COM	COM1.1	Aug	0.27 ± 0.09	Adjusted R^2 = −0.0053402, Slope = 3.7883e−05, p = 0.97038
10	COM	COM1.1	Aug	0.27 ± 0.13	
15	COM	COM1.1	Aug	0.28 ± 0.1	
20	COM	COM1.1	Aug	0.29 ± 0.1	
25	COM	COM1.1	Aug	0.28 ± 0.11	
30	COM	COM1.1	Aug	0.24 ± 0.02	
5	COM	COM1.1	Sep	0.31 ± 0.08	Adjusted R^2 = 0.0071517, Slope = 0.0011277, p = 0.12664
10	COM	COM1.1	Sep	0.31 ± 0.07	
15	COM	COM1.1	Sep	0.33 ± 0.08	
20	COM	COM1.1	Sep	0.32 ± 0.08	
25	COM	COM1.1	Sep	0.37 ± 0.08	
30	COM	COM1.1	Sep	0.28 ± 0.03	
5	COM	COM1.1	Oct	0.33 ± 0.04	Adjusted R^2 = −0.0036961, Slope = 0.0002591, p = 0.57976
10	COM	COM1.1	Oct	0.32 ± 0.03	
15	COM	COM1.1	Oct	0.32 ± 0.05	
20	COM	COM1.1	Oct	0.35 ± 0.06	
25	COM	COM1.1	Oct	0.36 ± 0.07	
30	COM	COM1.1	Oct	0.27 ± 0.01	
5	COM	COM1.1	Nov	0.3 ± 0.09	Adjusted R^2 = 0.049868, Slope = 0.0027457, p = 0.0011713
10	COM	COM1.1	Nov	0.3 ± 0.08	
15	COM	COM1.1	Nov	0.33 ± 0.11	
20	COM	COM1.1	Nov	0.31 ± 0.05	

25	COM	COM1.1	Nov	0.39 ± 0.05	
30	COM	COM1.1	Nov	0.33 ± 0.12	
5	COM	COM1.2	Jun	0.25 ± 0.13	Adjusted R^2 = 0.090741, Slope = 0.0047202, p = 1.5013e−05
10	COM	COM1.2	Jun	0.24 ± 0.1	
15	COM	COM1.2	Jun	0.29 ± 0.1	
20	COM	COM1.2	Jun	0.31 ± 0.09	
25	COM	COM1.2	Jun	0.38 ± 0.09	
30	COM	COM1.2	Jun	0.3 ± 0.05	
5	COM	COM1.2	Jul	0.22 ± 0.07	Adjusted R^2 = 0.13504, Slope = 0.0034607, p = 1.1807e−07
10	COM	COM1.2	Jul	0.25 ± 0.06	
15	COM	COM1.2	Jul	0.26 ± 0.07	
20	COM	COM1.2	Jul	0.29 ± 0.07	
25	COM	COM1.2	Jul	0.3 ± 0.06	
30	COM	COM1.2	Jul	0.27 ± 0.04	
5	COM	COM1.2	Aug	0.3 ± 0.11	Adjusted R^2 = 0.046179, Slope = 0.0034017, p = 0.0017334
10	COM	COM1.2	Aug	0.29 ± 0.12	
15	COM	COM1.2	Aug	0.32 ± 0.11	
20	COM	COM1.2	Aug	0.34 ± 0.11	
25	COM	COM1.2	Aug	0.36 ± 0.1	
30	COM	COM1.2	Aug	0.38 ± 0.06	
5	COM	COM1.2	Sep	0.26 ± 0.1	Adjusted R^2 = 0.0029002, Slope = 0.0012089, p = 0.21516
10	COM	COM1.2	Sep	0.26 ± 0.09	
15	COM	COM1.2	Sep	0.25 ± 0.1	
20	COM	COM1.2	Sep	0.28 ± 0.11	
25	COM	COM1.2	Sep	0.28 ± 0.11	
30	COM	COM1.2	Sep	0.3 ± 0.07	
5	COM	COM1.2	Oct	0.32 ± 0.05	Adjusted R^2 = 0.042788, Slope = −0.001594,, p = 0.0024864
10	COM	COM1.2	Oct	0.32 ± 0.05	
15	COM	COM1.2	Oct	0.31 ± 0.04	
20	COM	COM1.2	Oct	0.34 ± 0.05	
25	COM	COM1.2	Oct	0.32 ± 0.03	
30	COM	COM1.2	Oct	0.21 ± 0.02	
5	COM	COM1.2	Nov	0.3 ± 0.1	Adjusted R^2 = −0.0019544, Slope = −0.00079315, p = 0.42716
10	COM	COM1.2	Nov	0.29 ± 0.11	
15	COM	COM1.2	Nov	0.3 ± 0.12	
20	COM	COM1.2	Nov	0.3 ± 0.08	
25	COM	COM1.2	Nov	0.28 ± 0.12	
30	COM	COM1.2	Nov	0.26 ± 0.07	
5	COM	COM2.1	Jun	0.28 ± 0.14	Adjusted R^2 = 0.095697, Slope = 0.0059484, p = 8.8011e−06
10	COM	COM2.1	Jun	0.3 ± 0.14	
15	COM	COM2.1	Jun	0.34 ± 0.14	
20	COM	COM2.1	Jun	0.34 ± 0.13	
25	COM	COM2.1	Jun	0.35 ± 0.12	

30	COM	COM2.1	Jun	0.5 ± 0.07	
5	COM	COM2.1	Jul	0.27 ± 0.1	Adjusted R^2 = 0.054941, Slope = 0.0031094, p = 0.00068347
10	COM	COM2.1	Jul	0.29 ± 0.1	
15	COM	COM2.1	Jul	0.29 ± 0.09	
20	COM	COM2.1	Jul	0.32 ± 0.09	
25	COM	COM2.1	Jul	0.35 ± 0.07	
30	COM	COM2.1	Jul	0.34 ± 0.03	
5	COM	COM2.1	Aug	0.25 ± 0.1	Adjusted R^2 = 0.16264, Slope = 0.0064597, p = 5.2375e−09
10	COM	COM2.1	Aug	0.27 ± 0.11	
15	COM	COM2.1	Aug	0.29 ± 0.12	
20	COM	COM2.1	Aug	0.33 ± 0.12	
25	COM	COM2.1	Aug	0.36 ± 0.11	
30	COM	COM2.1	Aug	0.46 ± 0.06	
5	COM	COM2.1	Sep	0.3 ± 0.08	Adjusted R^2 = 0.14176, Slope = 0.004596, p = 5.5704e−08
10	COM	COM2.1	Sep	0.32 ± 0.09	
15	COM	COM2.1	Sep	0.34 ± 0.09	
20	COM	COM2.1	Sep	0.36 ± 0.09	
25	COM	COM2.1	Sep	0.39 ± 0.07	
30	COM	COM2.1	Sep	0.4 ± 0.09	
5	COM	COM2.1	Oct	0.32 ± 0.11	Adjusted R^2 = 0.054605, Slope = 0.0037181, p = 0.00070834
10	COM	COM2.1	Oct	0.32 ± 0.1	
15	COM	COM2.1	Oct	0.34 ± 0.11	
20	COM	COM2.1	Oct	0.38 ± 0.12	
25	COM	COM2.1	Oct	0.38 ± 0.14	
30	COM	COM2.1	Oct	0.41 ± 0.12	
5	COM	COM2.1	Nov	0.24 ± 0.13	Adjusted R^2 = −0.0038514, Slope = 0.00058569, p = 0.59817
10	COM	COM2.1	Nov	0.22 ± 0.11	
15	COM	COM2.1	Nov	0.25 ± 0.11	
20	COM	COM2.1	Nov	0.23 ± 0.09	
25	COM	COM2.1	Nov	0.29 ± 0.14	
30	COM	COM2.1	Nov	0.19 ± 0.04	
5	COM	COM2.2	Jun	0.23 ± 0.08	Adjusted R^2 = 0.12932, Slope = 0.0045294, p = 2.2308e−07
10	COM	COM2.2	Jun	0.25 ± 0.09	
15	COM	COM2.2	Jun	0.26 ± 0.1	
20	COM	COM2.2	Jun	0.3 ± 0.08	
25	COM	COM2.2	Jun	0.34 ± 0.06	
30	COM	COM2.2	Jun	0.31 ± 0.06	
5	COM	COM2.2	Jul	0.31 ± 0.09	Adjusted R^2 = 0.020593, Slope = 0.0022251, p = 0.027241
10	COM	COM2.2	Jul	0.32 ± 0.13	
15	COM	COM2.2	Jul	0.34 ± 0.09	
20	COM	COM2.2	Jul	0.3 ± 0.09	
25	COM	COM2.2	Jul	0.35 ± 0.07	
30	COM	COM2.2	Jul	0.41 ± 0.1	

5	COM	COM2.2	Aug	0.3 ± 0.09	Adjusted R^2 = 0.010006, Slope = 0.0014964, p = 0.090231
10	COM	COM2.2	Aug	0.3 ± 0.09	
15	COM	COM2.2	Aug	0.3 ± 0.09	
20	COM	COM2.2	Aug	0.31 ± 0.1	
25	COM	COM2.2	Aug	0.34 ± 0.09	
30	COM	COM2.2	Aug	0.33 ± 0.07	
5	COM	COM2.2	Sep	0.34 ± 0.11	Adjusted R^2 = 0.081503, Slope = 0.0045081, p = 4.0438e−05
10	COM	COM2.2	Sep	0.34 ± 0.1	
15	COM	COM2.2	Sep	0.35 ± 0.12	
20	COM	COM2.2	Sep	0.38 ± 0.11	
25	COM	COM2.2	Sep	0.42 ± 0.13	
30	COM	COM2.2	Sep	0.5 ± 0.05	
5	COM	COM2.2	Oct	0.39 ± 0.09	Adjusted R^2 = 0.10714, Slope = 0.0042844, p = 2.5481e−06
10	COM	COM2.2	Oct	0.41 ± 0.11	
15	COM	COM2.2	Oct	0.42 ± 0.09	
20	COM	COM2.2	Oct	0.45 ± 0.1	
25	COM	COM2.2	Oct	0.48 ± 0.06	
30	COM	COM2.2	Oct	0.5 ± 0.05	
5	COM	COM2.2	Nov	0.36 ± 0.09	Adjusted R^2 = 0.0093858, Slope = 0.0013916, p = 0.097047
10	COM	COM2.2	Nov	0.37 ± 0.08	
15	COM	COM2.2	Nov	0.38 ± 0.08	
20	COM	COM2.2	Nov	0.37 ± 0.1	
25	COM	COM2.2	Nov	0.37 ± 0.1	
30	COM	COM2.2	Nov	0.43 ± 0.08	
5	COM	COM3.1	Jun	0.21 ± 0.1	Adjusted R^2 = 0.20104, Slope = 0.0062805, p = 5.8898e−11
10	COM	COM3.1	Jun	0.24 ± 0.1	
15	COM	COM3.1	Jun	0.27 ± 0.09	
20	COM	COM3.1	Jun	0.32 ± 0.09	
25	COM	COM3.1	Jun	0.34 ± 0.07	
30	COM	COM3.1	Jun	0.34 ± 0.09	
5	COM	COM3.1	Jul	0.31 ± 0.08	Adjusted R^2 = 0.0047314, Slope = 0.0013471, p = 0.17043
10	COM	COM3.1	Jul	0.35 ± 0.11	
15	COM	COM3.1	Jul	0.33 ± 0.11	
20	COM	COM3.1	Jul	0.38 ± 0.12	
25	COM	COM3.1	Jul	0.34 ± 0.09	
30	COM	COM3.1	Jul	0.29 ± 0.02	
5	COM	COM3.1	Aug	0.3 ± 0.12	Adjusted R^2 = 0.090641, Slope = 0.0043752, p = 1.5175e−05
10	COM	COM3.1	Aug	0.34 ± 0.08	
15	COM	COM3.1	Aug	0.36 ± 0.09	
20	COM	COM3.1	Aug	0.36 ± 0.1	
25	COM	COM3.1	Aug	0.35 ± 0.11	
30	COM	COM3.1	Aug	0.46 ± 0.08	
5	COM	COM3.1	Sep	0.25 ± 0.06	Adjusted R^2 = 0.18421,

10	COM	COM3.1	Sep	0.26 ± 0.05	Slope = 0.0047492, p = 4.3097e−10
15	COM	COM3.1	Sep	0.3 ± 0.08	
20	COM	COM3.1	Sep	0.33 ± 0.12	
25	COM	COM3.1	Sep	0.37 ± 0.07	
30	COM	COM3.1	Sep	0.31 ± 0.07	
5	COM	COM3.1	Oct	0.36 ± 0.08	Adjusted R^2 = 0.1254 , Slope = 0.0040766, p = 3.4431e−07
10	COM	COM3.1	Oct	0.38 ± 0.08	
15	COM	COM3.1	Oct	0.38 ± 0.08	
20	COM	COM3.1	Oct	0.42 ± 0.08	
25	COM	COM3.1	Oct	0.47 ± 0.06	
30	COM	COM3.1	Oct	0.42 ± 0.08	
5	COM	COM3.1	Nov	0.33 ± 0.11	Adjusted R^2 = 0.048227, Slope = 0.0036676, p = 0.0013944
10	COM	COM3.1	Nov	0.35 ± 0.11	
15	COM	COM3.1	Nov	0.36 ± 0.12	
20	COM	COM3.1	Nov	0.39 ± 0.12	
25	COM	COM3.1	Nov	0.4 ± 0.11	
30	COM	COM3.1	Nov	0.41 ± 0.16	
5	COM	COM3.2	Jun	0.28 ± 0.08	Adjusted R^2 = 0.086836, Slope = 0.0035382, p = 2.2837e−05
10	COM	COM3.2	Jun	0.3 ± 0.09	
15	COM	COM3.2	Jun	0.27 ± 0.07	
20	COM	COM3.2	Jun	0.32 ± 0.09	
25	COM	COM3.2	Jun	0.37 ± 0.09	
30	COM	COM3.2	Jun	0.38 ± 0.07	
5	COM	COM3.2	Jul	0.23 ± 0.09	Adjusted R^2 = 0.20567, Slope = 0.0064761, p = 3.3867e−11
10	COM	COM3.2	Jul	0.28 ± 0.08	
15	COM	COM3.2	Jul	0.32 ± 0.1	
20	COM	COM3.2	Jul	0.34 ± 0.09	
25	COM	COM3.2	Jul	0.36 ± 0.12	
30	COM	COM3.2	Jul	0.39 ± 0.12	
5	COM	COM3.2	Aug	0.27 ± 0.11	Adjusted R^2 = −0.0045358, Slope = 0.00038781, p = 0.6979
10	COM	COM3.2	Aug	0.26 ± 0.1	
15	COM	COM3.2	Aug	0.26 ± 0.1	
20	COM	COM3.2	Aug	0.28 ± 0.11	
25	COM	COM3.2	Aug	0.29 ± 0.11	
30	COM	COM3.2	Aug	0.26 ± 0.1	
5	COM	COM3.2	Sep	0.38 ± 0.1	Adjusted R^2 = −0.0052945, Slope = 9.6779e−05, p = 0.92094
10	COM	COM3.2	Sep	0.4 ± 0.11	
15	COM	COM3.2	Sep	0.38 ± 0.09	
20	COM	COM3.2	Sep	0.38 ± 0.1	
25	COM	COM3.2	Sep	0.39 ± 0.1	
30	COM	COM3.2	Sep	0.4 ± 0.08	
5	COM	COM3.2	Oct	0.38 ± 0.07	Adjusted R^2 = −0.0021766, Slope =
10	COM	COM3.2	Oct	0.38 ± 0.06	

15	COM	COM3.2	Oct	0.4 ± 0.07	0.00052037, p = 0.44274
20	COM	COM3.2	Oct	0.41 ± 0.06	
25	COM	COM3.2	Oct	0.45 ± 0.05	
30	COM	COM3.2	Oct	0.29 ± 0.05	
5	COM	COM3.2	Nov	0.38 ± 0.08	Adjusted R^2 = −0.0051311, Slope = 0.0001532, p = 0.84117
10	COM	COM3.2	Nov	0.38 ± 0.07	
15	COM	COM3.2	Nov	0.39 ± 0.07	
20	COM	COM3.2	Nov	0.37 ± 0.09	
25	COM	COM3.2	Nov	0.35 ± 0.06	
30	COM	COM3.2	Nov	0.44 ± 0.08	
5	RES	RES1	Jun	0.24 ± 0.06	Adjusted R^2 = 0.015972, Slope = 0.0011288, p = 0.045567
10	RES	RES1	Jun	0.24 ± 0.05	
15	RES	RES1	Jun	0.25 ± 0.05	
20	RES	RES1	Jun	0.25 ± 0.06	
25	RES	RES1	Jun	0.28 ± 0.07	
30	RES	RES1	Jun	0.25 ± 0.02	
5	RES	RES1	Jul	0.31 ± 0.09	Adjusted R^2 = 0.002998, Slope = 0.0010545, p = 0.21245
10	RES	RES1	Jul	0.3 ± 0.08	
15	RES	RES1	Jul	0.32 ± 0.09	
20	RES	RES1	Jul	0.32 ± 0.09	
25	RES	RES1	Jul	0.3 ± 0.1	
30	RES	RES1	Jul	0.36 ± 0.09	
5	RES	RES1	Aug	0.25 ± 0.12	Adjusted R^2 = −0.0035844, Slope = 0.0006176, p = 0.56721
10	RES	RES1	Aug	0.26 ± 0.11	
15	RES	RES1	Aug	0.23 ± 0.1	
20	RES	RES1	Aug	0.27 ± 0.12	
25	RES	RES1	Aug	0.27 ± 0.08	
30	RES	RES1	Aug	0.26 ± 0.05	
5	RES	RES1	Sep	0.39 ± 0.04	Adjusted R^2 = 0.027958, Slope = −0.00090685, p = 0.012189
10	RES	RES1	Sep	0.38 ± 0.04	
15	RES	RES1	Sep	0.38 ± 0.03	
20	RES	RES1	Sep	0.38 ± 0.03	
25	RES	RES1	Sep	0.39 ± 0.03	
30	RES	RES1	Sep	0.32 ± 0.04	
5	RES	RES1	Oct	0.35 ± 0.05	Adjusted R^2 = 0.012645, Slope = −0.0009997, p = 0.066472
10	RES	RES1	Oct	0.35 ± 0.06	
15	RES	RES1	Oct	0.33 ± 0.06	
20	RES	RES1	Oct	0.35 ± 0.05	
25	RES	RES1	Oct	0.34 ± 0.06	
30	RES	RES1	Oct	0.31 ± 0.05	
5	RES	RES1	Nov	0.39 ± 0.09	Adjusted R^2 = 0.0020623, Slope = −0.00097411, p =
10	RES	RES1	Nov	0.38 ± 0.08	
15	RES	RES1	Nov	0.38 ± 0.08	

20	RES	RES1	Nov	0.37 ± 0.09	0.24015
25	RES	RES1	Nov	0.4 ± 0.09	
30	RES	RES1	Nov	0.32 ± 0.03	
5	RES	RES2	Jun	0.28 ± 0.06	Adjusted R^2 = 0.02799, Slope = 0.0015152, p = 0.012147
10	RES	RES2	Jun	0.29 ± 0.07	
15	RES	RES2	Jun	0.3 ± 0.06	
20	RES	RES2	Jun	0.31 ± 0.06	
25	RES	RES2	Jun	0.31 ± 0.04	
30	RES	RES2	Jun	0.31 ± 0.03	
5	RES	RES2	Jul	0.32 ± 0.07	Adjusted R^2 = −0.00021809, Slope = −0.00071204, p = 0.3287
10	RES	RES2	Jul	0.32 ± 0.08	
15	RES	RES2	Jul	0.31 ± 0.08	
20	RES	RES2	Jul	0.31 ± 0.08	
25	RES	RES2	Jul	0.32 ± 0.07	
30	RES	RES2	Jul	0.27 ± 0.05	
5	RES	RES2	Aug	0.31 ± 0.1	Adjusted R^2 = −0.0023611, Slope = 0.0007302, p = 0.45634
10	RES	RES2	Aug	0.28 ± 0.09	
15	RES	RES2	Aug	0.29 ± 0.1	
20	RES	RES2	Aug	0.33 ± 0.1	
25	RES	RES2	Aug	0.27 ± 0.1	
30	RES	RES2	Aug	0.36 ± 0.1	
5	RES	RES2	Sep	0.43 ± 0.04	Adjusted R^2 = −0.00078003, Slope = 0.00040446, p = 0.35676
10	RES	RES2	Sep	0.43 ± 0.04	
15	RES	RES2	Sep	0.44 ± 0.05	
20	RES	RES2	Sep	0.45 ± 0.04	
25	RES	RES2	Sep	0.45 ± 0.04	
30	RES	RES2	Sep	0.41 ± 0.04	
5	RES	RES2	Oct	0.42 ± 0.07	Adjusted R^2 = 0.080014, Slope = 0.002784, p = 4.7421e−05
10	RES	RES2	Oct	0.43 ± 0.07	
15	RES	RES2	Oct	0.44 ± 0.08	
20	RES	RES2	Oct	0.46 ± 0.06	
25	RES	RES2	Oct	0.48 ± 0.06	
30	RES	RES2	Oct	0.47 ± 0.05	
5	RES	RES2	Nov	0.37 ± 0.05	Adjusted R^2 = −0.0051827, Slope = 8.8182e−05, p = 0.86116
10	RES	RES2	Nov	0.37 ± 0.05	
15	RES	RES2	Nov	0.36 ± 0.05	
20	RES	RES2	Nov	0.37 ± 0.06	
25	RES	RES2	Nov	0.39 ± 0.05	
30	RES	RES2	Nov	0.35 ± 0.06	
5	RES	RES3	Jun	0.37 ± 0.11	Adjusted R^2 = 0.037518, Slope = 0.0029185, p = 0.0043622
10	RES	RES3	Jun	0.38 ± 0.1	
15	RES	RES3	Jun	0.4 ± 0.12	
20	RES	RES3	Jun	0.39 ± 0.1	

25	RES	RES3	Jun	0.42 ± 0.09	
30	RES	RES3	Jun	0.48 ± 0.04	
5	RES	RES3	Jul	0.32 ± 0.11	Adjusted R^2 = −0.0032358, Slope = −0.00058387, p = 0.53117
10	RES	RES3	Jul	0.3 ± 0.09	
15	RES	RES3	Jul	0.33 ± 0.1	
20	RES	RES3	Jul	0.28 ± 0.08	
25	RES	RES3	Jul	0.33 ± 0.09	
30	RES	RES3	Jul	0.3 ± 0.06	
5	RES	RES3	Aug	0.34 ± 0.12	Adjusted R^2 = 0.02191, Slope = −0.0024024, p = 0.023566
10	RES	RES3	Aug	0.28 ± 0.09	
15	RES	RES3	Aug	0.32 ± 0.12	
20	RES	RES3	Aug	0.28 ± 0.1	
25	RES	RES3	Aug	0.3 ± 0.1	
30	RES	RES3	Aug	0.24 ± 0.07	
5	RES	RES3	Sep	0.39 ± 0.06	Adjusted R^2 = −0.00071872, Slope = −0.00052978, p = 0.35355
10	RES	RES3	Sep	0.38 ± 0.06	
15	RES	RES3	Sep	0.38 ± 0.06	
20	RES	RES3	Sep	0.37 ± 0.07	
25	RES	RES3	Sep	0.38 ± 0.05	
30	RES	RES3	Sep	0.38 ± 0.06	
5	RES	RES3	Oct	0.39 ± 0.07	Adjusted R^2 = −0.0049884, Slope = −0.00019625, p = 0.79629
10	RES	RES3	Oct	0.39 ± 0.08	
15	RES	RES3	Oct	0.37 ± 0.07	
20	RES	RES3	Oct	0.37 ± 0.08	
25	RES	RES3	Oct	0.42 ± 0.06	
30	RES	RES3	Oct	0.37 ± 0.11	
5	RES	RES3	Nov	0.39 ± 0.07	Adjusted R^2 = −0.0052096, Slope = 0.00010686, p = 0.87289
10	RES	RES3	Nov	0.36 ± 0.07	
15	RES	RES3	Nov	0.37 ± 0.06	
20	RES	RES3	Nov	0.37 ± 0.08	
25	RES	RES3	Nov	0.36 ± 0.04	
30	RES	RES3	Nov	0.43 ± 0.07	
5	RES	RES4	Jun	0.29 ± 0.06	Adjusted R^2 = 0.010945, Slope = 0.00096089, p = 0.080878
10	RES	RES4	Jun	0.3 ± 0.06	
15	RES	RES4	Jun	0.3 ± 0.06	
20	RES	RES4	Jun	0.3 ± 0.06	
25	RES	RES4	Jun	0.29 ± 0.03	
30	RES	RES4	Jun	0.34 ± 0.03	
5	RES	RES4	Jul	0.34 ± 0.09	Adjusted R^2 = 0.01824, Slope = 0.0019966, p = 0.035355
10	RES	RES4	Jul	0.33 ± 0.09	
15	RES	RES4	Jul	0.32 ± 0.09	
20	RES	RES4	Jul	0.37 ± 0.11	
25	RES	RES4	Jul	0.39 ± 0.1	

30	RES	RES4	Jul	0.38 ± 0.11	
5	RES	RES4	Aug	0.29 ± 0.12	Adjusted R^2 = 0.057194, Slope = 0.0041351, p = 0.00053811
10	RES	RES4	Aug	0.31 ± 0.13	
15	RES	RES4	Aug	0.31 ± 0.12	
20	RES	RES4	Aug	0.33 ± 0.13	
25	RES	RES4	Aug	0.38 ± 0.12	
30	RES	RES4	Aug	0.42 ± 0.08	
5	RES	RES4	Sep	0.39 ± 0.08	Adjusted R^2 = −0.0024249, Slope = −0.00058546, p = 0.4612
10	RES	RES4	Sep	0.38 ± 0.08	
15	RES	RES4	Sep	0.38 ± 0.08	
20	RES	RES4	Sep	0.39 ± 0.09	
25	RES	RES4	Sep	0.37 ± 0.08	
30	RES	RES4	Sep	0.37 ± 0.09	
5	RES	RES4	Oct	0.35 ± 0.05	Adjusted R^2 = −0.002954, Slope = 0.00031515, p = 0.50493
10	RES	RES4	Oct	0.36 ± 0.05	
15	RES	RES4	Oct	0.36 ± 0.05	
20	RES	RES4	Oct	0.36 ± 0.04	
25	RES	RES4	Oct	0.36 ± 0.03	
30	RES	RES4	Oct	0.36 ± 0.03	
5	RES	RES4	Nov	0.37 ± 0.06	Adjusted R^2 = −0.0042914, Slope = 0.00027254, p = 0.65794
10	RES	RES4	Nov	0.38 ± 0.06	
15	RES	RES4	Nov	0.36 ± 0.06	
20	RES	RES4	Nov	0.37 ± 0.07	
25	RES	RES4	Nov	0.39 ± 0.07	
30	RES	RES4	Nov	0.38 ± 0.08	

Abbreviation: COM, commercial building; RES, residential household; Jun, June; Jul, July; Aug, August; Sep, September; Oct, October; Nov, November.
*Naming convention encompass (i) site type, (ii) site, (iii) time point, and (iv) month. See Example 1 and Example 2 below.
Example 1: COM3.2.1.6 (i) Commercial building (ii) site 3.2, (iii) time point 1, (iv) month 6
Example 2: RES4.7.6 (i) Residential household (ii) site 4, (iii) time point 7, (iv) month 6

Table S14: Bray-Curtis distance based PERMANOVA, ANOVA, and Tukey HSD test using flow cytometric fingerprinting data of commercial building and residential household $TP_{5\text{-}30}$ samples.

A. Mean pairwise Bray-Curtis dissimilarity distances between monthly $TP_{5\text{-}30}$ samples of commercial building and residential household sites. Bray-Curtis distances was determined using flow cytometric parameters and the explanatory power of site type (commercial building vs. residential household) with respect to microbial community structure of each month were assessed using PERMANOVA.

**Month**	**Bray-Curtis dissimilarity distance (M ± SD)**	**PERMANOVA based on site type**
Jun	0.33 ± 0.07	F(1,58) = 22.41,R2 =0.28 p =0.001
Jul	0.31 ± 0.09	F(1,58) = 15.39,R2 =0.21 p =0.001
Aug	0.31 ± 0.1	F(1,58) = 10.73,R2 =0.16 p =0.001

Sep	0.32 ± 0.09	F(1,58) = 11.82,R2 =0.17 p =0.001
Oct	0.27 ± 0.06	F(1,58) = 2.61,R2 =0.04 p =0.003
Nov	0.28 ± 0.05	F(1,58) = 7.8,R2 =0.12 p =0.001

Abbreviation: Jun, June; Jul, July; Aug, August; Sep, September; Oct, October; Nov, November.

B. ANOVA and Tukey HSD test for differences in Bray-Curtis dissimilarity distances between monthly $TP_{5\text{-}30}$ samples of commercial building and residential household sites.

	**Df**	**Sum Sq**	**Mean Sq**	**F value**	**Pr(>F)**
Month	5	1.01	0.20	32.29	7.839E-32
Residuals	2154	13.44	0.01		

**Comparison**	**diff**	**lwr**	**upr**	**p adj**
Aug-Jul	-0.026	-0.043	-0.009	1.73E-04
Aug-Jun	-0.047	-0.064	-0.030	6.62E-11
Jul-Jun	-0.021	-0.038	-0.004	4.27E-03
Nov-Aug	0.001	-0.015	0.018	1.00E+00
Nov-Jul	-0.024	-0.041	-0.008	5.13E-04
Nov-Jun	-0.046	-0.062	-0.029	6.63E-11
Nov-Oct	0.012	-0.005	0.029	2.97E-01
Nov-Sep	-0.038	-0.055	-0.021	1.75E-09
Oct-Aug	-0.011	-0.028	0.006	4.44E-01
Oct-Jul	-0.037	-0.053	-0.020	8.77E-09
Oct-Jun	-0.058	-0.075	-0.041	6.61E-11
Oct-Sep	-0.050	-0.067	-0.034	6.61E-11
Sep-Aug	0.040	0.023	0.056	3.91E-10
Sep-Jul	0.014	-0.003	0.031	1.78E-01
Sep-Jun	-0.007	-0.024	0.009	8.03E-01

Abbreviation: Jun, June; Jul, July; Aug, August; Sep, September; Oct, October; Nov, November.

Table S15: Mean pairwise Bray-Curtis dissimilarity distances between monthly TP_{30} samples of commercial building and residential household sites. Bray-Curtis distances was determined using 16S rRNA gene sequencing data and the explanatory power of site type (commercial building vs. residential household) with respect to microbial community structure of each month were assessed using PERMANOVA.

**Month**	**Bray-Curtis dissimilarity distance (M ± SD)**	**PERMANOVA based on site type**
Jun	0.72 ± 0.2	F(1, 8) = 3.08 R2 = 0.28 , p = 0.014
Jul	0.63 ± 0.17	F(1, 8) = 2.71 R2 = 0.25 , p = 0.014
Aug	0.58 ± 0.14	F(1, 8) = 2.17 R2 = 0.21 , p = 0.038
Sep	0.54 ± 0.11	F(1, 8) = 2.25 R2 = 0.22 , p = 0.025
Oct	0.56 ± 0.1	F(1, 8) = 2.62 R2 = 0.25 , p = 0.015
Nov	0.66 ± 0.19	F(1, 8) = 3.33 R2 = 0.25 , p = 0.017

Abbreviation: Jun, June; Jul, July; Aug, August; Sep, September; Oct, October; Nov, November.

Table S16: Betadispersivity, ANOVA, and Tukey HSD test for TP_{30} samples grouped by commercial building and residential household sites within each month using 16S rRNA gene sequencing data.

A. Betadispersivity calculated as the average Bray-Curtis dissimilarity distance between TP_{30} samples grouped by commercial building and residential household sites within each month.

**Site type**	**Month**	**Distance to centroid**	**SD**
COM	Aug	0.39	0.08
COM	Jul	0.43	0.08
COM	Jun	0.50	0.09
COM	Nov	0.44	0.07
COM	Oct	0.36	0.04
COM	Sep	0.36	0.06
RES	Aug	0.25	0.06
RES	Jul	0.21	0.05
RES	Jun	0.20	0.03
RES	Nov	0.19	0.02
RES	Oct	0.23	0.03
RES	Sep	0.23	0.04

Abbreviation: COM, commercial building; RES, residential household; Jun, June; Jul, July; Aug, August; Sep, September; Oct, October; Nov, November.

B. ANOVA and Tukey HSD test for differences in Bray-Curtis betadispersivity between monthly TP_{30} samples of commercial building and residential household sites.

	**Degrees of freedom**	**Sum of squares**	**R2**	**F**	**Pr(>F)**
**Site type_month**	11	0.65	0.06	16.08	1.43E-12
**Residual**	48	0.18	0.00		

**Comparison**	**diff**	**lwr**	**upr**	**p adj**
RES_Nov-COM_Jun	-0.31	-0.44	-0.17	0.000
RES_Jun-COM_Jun	-0.30	-0.44	-0.17	0.000
RES_Jul-COM_Jun	-0.29	-0.42	-0.15	0.000
RES_Sep-COM_Jun	-0.27	-0.40	-0.14	0.000
RES_Oct-COM_Jun	-0.26	-0.40	-0.13	0.000
RES_Nov-COM_Nov	-0.25	-0.39	-0.12	0.000
RES_Jun-COM_Nov	-0.25	-0.38	-0.11	0.000
RES_Aug-COM_Jun	-0.24	-0.38	-0.11	0.000
RES_Nov-COM_Jul	-0.24	-0.38	-0.11	0.000
RES_Jun-COM_Jul	-0.24	-0.37	-0.10	0.000
RES_Jul-COM_Nov	-0.23	-0.36	-0.10	0.000

RES_Jul-COM_Jul	-0.22	-0.35	-0.09	0.000
RES_Sep-COM_Nov	-0.21	-0.35	-0.08	0.000
RES_Oct-COM_Nov	-0.21	-0.34	-0.07	0.000
RES_Sep-COM_Jul	-0.20	-0.34	-0.07	0.000
RES_Nov-COM_Aug	-0.20	-0.34	-0.07	0.000
RES_Oct-COM_Jul	-0.20	-0.33	-0.06	0.000
RES_Jun-COM_Aug	-0.20	-0.33	-0.06	0.000
RES_Aug-COM_Nov	-0.19	-0.32	-0.05	0.001
RES_Jul-COM_Aug	-0.18	-0.31	-0.05	0.002
RES_Aug-COM_Jul	-0.18	-0.31	-0.04	0.002
RES_Nov-COM_Oct	-0.17	-0.31	-0.04	0.003
RES_Nov-COM_Sep	-0.17	-0.31	-0.04	0.003
RES_Jun-COM_Oct	-0.17	-0.30	-0.03	0.005
RES_Jun-COM_Sep	-0.17	-0.30	-0.03	0.005
RES_Sep-COM_Aug	-0.16	-0.30	-0.03	0.006
RES_Oct-COM_Aug	-0.16	-0.29	-0.02	0.010
RES_Jul-COM_Oct	-0.15	-0.28	-0.02	0.015
COM_Sep-COM_Jun	-0.13	-0.25	-0.02	0.015
RES_Jul-COM_Sep	-0.15	-0.28	-0.02	0.016
COM_Oct-COM_Jun	-0.13	-0.25	-0.01	0.016
RES_Aug-COM_Aug	-0.14	-0.27	0.00	0.037
RES_Sep-COM_Oct	-0.13	-0.27	0.00	0.048
RES_Sep-COM_Sep	-0.13	-0.27	0.00	0.048
RES_Oct-COM_Oct	-0.13	-0.26	0.01	0.071
RES_Oct-COM_Sep	-0.13	-0.26	0.01	0.072
COM_Jun-COM_Aug	0.11	-0.01	0.23	0.127
RES_Aug-COM_Oct	-0.11	-0.24	0.02	0.208
RES_Aug-COM_Sep	-0.11	-0.24	0.02	0.209
COM_Sep-COM_Nov	-0.08	-0.20	0.04	0.514
COM_Oct-COM_Nov	-0.08	-0.20	0.04	0.518
COM_Sep-COM_Jul	-0.07	-0.19	0.05	0.701
COM_Oct-COM_Jul	-0.07	-0.19	0.05	0.704
COM_Jun-COM_Jul	0.07	-0.05	0.19	0.763
COM_Nov-COM_Jun	-0.06	-0.18	0.06	0.900
RES_Nov-RES_Aug	-0.06	-0.21	0.08	0.940
COM_Nov-COM_Aug	0.05	-0.07	0.17	0.949
RES_Jun-RES_Aug	-0.06	-0.20	0.09	0.970
COM_Jul-COM_Aug	0.04	-0.08	0.16	0.990
RES_Oct-RES_Nov	0.04	-0.10	0.19	0.996
RES_Jul-RES_Aug	-0.04	-0.19	0.11	0.998
RES_Oct-RES_Jun	0.04	-0.11	0.18	0.999
RES_Sep-RES_Nov	0.04	-0.11	0.18	0.999
COM_Sep-COM_Aug	-0.03	-0.15	0.09	0.999

COM_Oct-COM_Aug	-0.03	-0.15	0.09	1.000
RES_Sep-RES_Jun	0.03	-0.11	0.18	1.000
RES_Sep-RES_Aug	-0.02	-0.17	0.12	1.000
RES_Oct-RES_Jul	0.02	-0.12	0.17	1.000
RES_Nov-RES_Jul	-0.02	-0.17	0.12	1.000
RES_Oct-RES_Aug	-0.02	-0.17	0.13	1.000
RES_Jun-RES_Jul	-0.02	-0.16	0.13	1.000
RES_Sep-RES_Jul	0.02	-0.13	0.16	1.000
COM_Nov-COM_Jul	0.01	-0.11	0.13	1.000
COM_Sep-COM_Oct	0.00	-0.12	0.12	1.000
RES_Nov-RES_Jun	-0.01	-0.15	0.14	1.000
RES_Sep-RES_Oct	-0.01	-0.15	0.14	1.000

Abbreviation: COM, commercial building; RES, residential household; Jun, June; Jul, July; Aug, August; Sep, September; Oct, October; Nov, November.
